# Supplementary material for: Analysis of PPARγ Signaling Activity in Psoriasis
Source: Int J Mol Sci. 2021 Aug 10;22(16):8603. doi: 10.3390/ijms22168603 (PMC8395241; doi:10.3390/ijms22168603)
Supplement: Supplementary file 1 [file ijms-22-08603-s001.zip › Supplemental materials_Analysis of PPARg signaling activity in psoriasis/Pathway models/Models images and html files/Interleukin-17 and interleukin-22 signaling in psoriasis/11570.html]

TP53


# Protein TP53

|  |  |
| --- | --- |
| URN | urn:agi-llid:7157 |
| Total Entities | 0 |
| Connectivity | 19232 |
| Name | TP53 |
| Description | tumor protein p53 |
| Notes | This gene encodes a tumor suppressor protein containing transcriptional activation, DNA binding, and oligomerization domains. The encoded protein responds to diverse cellular stresses to regulate expression of target genes, thereby inducing cell cycle arrest, apoptosis, senescence, DNA repair, or changes in metabolism. Mutations in this gene are associated with a variety of human cancers, including hereditary cancers such as Li-Fraumeni syndrome. Alternative splicing of this gene and the use of alternate promoters result in multiple transcript variants and isoforms. Additional isoforms have also been shown to result from the use of alternate translation initiation codons from identical transcript variants (PMIDs: 12032546, 20937277). [provided by RefSeq, Dec 2016] |
| Primary Cell Localization | Nucleus |
| Class | Transcription factor |
| ObjectType | Protein |

---

|  |  |
| --- | --- |
| Pathway | Proteins Involved in Stem Cell Exhaustion in Aging |
|  | Alzheimer's Disease |
|  | Apoptosis of Cochlear Hair Cells (Noise-Induced, Mouse Model) |
|  | Oncogene Induced Cellular Senescence |
|  | DNA Persistent Repair Inhibits mTOR Signaling |
|  | mTOR Signaling |
|  | SIRT2 Signaling in Aging |
|  | SIRT3 Signaling in Aging |
|  | SIRT6 Signaling in Aging |
|  | SIRT7 Signaling in Aging |
|  | SIRT1 Signaling in Aging |
|  | Telomer Attrition Triggers Genomic Instability |
|  | MTOR/TP53 Regulates Cell Metabolism |
|  | Thyroid Hormones Common Genomic Effects in Hyperthyroidism |
|  | Thyroid Hormones Common non-Genomic Effects in Hyperthyroidism |
|  | Somatostatin Receptors Signaling |
|  | Cortisol in Resolving Inflammation |
|  | T-Cell Maturation (Hypothesis) |
|  | Neutrophil Recruitment and Priming |
|  | Apoptosis |
|  | G1/S Damage Checkpoint |
|  | Cell Cycle Overiew |
|  | G2/M Damage Checkpoint |
|  | SCF/FBXO11 Complex |
|  | SCF/BTRC Complex |
|  | SCF/SKP2 Complex |
|  | Telomere Maintenance |
|  | Histone Sumoylation |
|  | Proteins Involved in Dilated Cardiomyopathy |
|  | Proteins Involved in Myocardial Ischemia |
|  | Hair Follicle Keratinocyte Apoptosis |
|  | Androgens in Sebocyte Maturation |
|  | Melanoma |
|  | Apoptosis Block in Melanoma Cell |
|  | Genes with Mutations Associated with Melanoma |
|  | Proteins Involved in Melanoma |
|  | IL22 Induces Keratinocyte Proliferation in Psoriasis |
|  | Proteins Involved in Photoaging |
|  | Melanocyte Role in Skin Aging |
|  | POMC Activation in Keratinocytes |
|  | Proteins Involved in Diabetes Mellitus Type 1 |
|  | Proteins Involved in Diabetic Nephropathy |
|  | Proteins Involved in non-Alcoholic Fatty Liver Disease |
|  | beta-Cell Destruction in Diabetes Mellitus Type 1 |
|  | Apoptosis of Thyroid Cell in Hashimoto's Thyroiditis |
|  | Proteins Involved in Obesity |
|  | Proteins Involved in Colorectal Neoplasms |
|  | Cetuximab Resistance in Colorectal Cancer |
|  | Metastatic Colorectal Cancer |
|  | GUCY2C Signaling in Colorectal Cancer |
|  | Peutz-Jeghers Syndrome |
|  | Proteins Involved in Peutz-Jeghers Syndrome |
|  | Proteins Involved in Ulcerative Colitis |
|  | Proteins Involved in Acute Myeloid Leukemia |
|  | Apoptosis Block in Acute Myeloid Leukemia Cell |
|  | Acute Myeloid Leukemia |
|  | B-Cell Acute Lymphoblastic Leukemia |
|  | Proteins Involved in B-Cell Chronic Lymphocytic Leukemia |
|  | B-Cell Chronic Lymphocytic Leukemia |
|  | Chronic Myeloid Leukemia |
|  | Proteins Involved in Chronic Myeloid Leukemia |
|  | Diffuse Large-B-Cell Lymphoma |
|  | Diffuse Large-B-Cell Lymphoma ABC Subtype |
|  | Proteins Involved in Diffuse Large-B-Cell Lymphoma |
|  | Lymphoma, GCB Subtype |
|  | Primary Mediastinal B-cell Lymphoma (PMBL) Subtype |
|  | Follicular Lymphoma |
|  | Proteins Involved in Follicular Lymphoma |
|  | Proteins Involved in Hodgkin Lymphoma |
|  | Hodgkin Lymphoma |
|  | Proteins Involved in Mantle Cell Lymphoma |
|  | Mantle Cell Lymphoma |
|  | Cell Cycle Dysregulation in Mantle Cell Lymphoma |
|  | Apoptosis of Mantle Cell Deregulation |
|  | mTOR/NF-kB/BCR Signaling Disregulation in Mantle Cell Lymphoma |
|  | Mucosa-Associated Lymphoid Tissue (MALT) Lymphoma |
|  | Splenic Marginal Zone Lymphoma |
|  | Proteins Involved in Marginal Zone Lymphoma |
|  | IL6/IGF1/VEGFA Signaling in Multiple Myeloma |
|  | Multiple Myeloma |
|  | Proteins Involved in Multiple Myeloma |
|  | Proteins Involved in T-Cell Acute Lymphoblastic Leukemia |
|  | T-Cell Acute Lymphoblastic Leukemia |
|  | Proteins Involved in Hepatocellular Carcinoma |
|  | Hepatocellular Carcinoma |
|  | TP53 Signaling in Hepatocellular Carcinoma |
|  | Growth Factor Signaling in Hepatocellular Carcinoma |
|  | Proteins Involved in Pancreatic Cancer |
|  | Pancreatic Ductal Carcinoma |
|  | Growth Factor Signaling in Pancreatic Neoplasms |
|  | TP53 Signaling in Pancreatic Neoplasms |
|  | STK11 Signaling in Pancreatic Neoplasms |
|  | Proteins Involved in Psoriatic Arthritis |
|  | Proteins Involved in Rheumatoid Arthritis |
|  | FXN Role in Friedreich Ataxia Progression |
|  | Li-Fraumeni Syndrome |
|  | Hereditary Breast and Ovarian Cancer Syndrome |
|  | Cowden Syndrome |
|  | Huntingtin Gene Mutation in Striatal Neuron |
|  | Proteins Involved in Huntington Disease |
|  | Angelman Syndrome: Effects of Mutation of Maternal Copy of UBE3A Gene |
|  | Proteins Involved in Angelman Syndrome |
|  | beta-Casomorphins/Opioid Receptors Affect Brain Function (Hypothesis) |
|  | Werner Syndrome (Adult Progeria) |
|  | Proteins Involved in Werner Syndrome (Adult Progeria) |
|  | Proteins Involved in Hutchinson-Gilford Progeria Sindrome |
|  | Muscular Dystrophy, Facioscapulohumeral |
|  | Proteins Involved in Alzheimer's Disease |
|  | Proteins Involved in Epilepsy |
|  | Apoptosis and Immediate Early Gene Activation in Epileptiform Disorders |
|  | Glioblastoma, Primary |
|  | Proteins Involved in Glioblastoma |
|  | Glioblastoma, Secondary |
|  | Astrocytoma |
|  | Proteins Involved in Astrocytoma |
|  | Proteins Involved in Glioma |
|  | Ependymoma |
|  | Medulloblastoma |
|  | Proteins Involved in Medulloblastoma |
|  | Neuroblastoma |
|  | non-Hereditary Genetic Rearrangements in Neuroblastoma |
|  | Proteins Involved in Neuroblastoma |
|  | PARK7 and LRRK2 in Young Onset Parkinson's Disease |
|  | Neurotoxin Effects in Parkinson's Disease |
|  | SOD1 Mutation |
|  | Proteins with Altered Expression in Amyotrophic Lateral Sclerosis |
|  | Proteins Involved in Amyotrophic Lateral Sclerosis |
|  | SPP1 Induced Survival of Myelin Specific T-Cells in Multiple Sclerosis |
|  | Proteins Involved in Breast Cancer Related to ESR1 Signaling Pathway |
|  | Proteins Involved in Breast Cancer Related to ERBB2/VEGFR/Akt Signaling Pathway |
|  | ERBB/VEGFR/Akt Signaling in Breast Cancer |
|  | Genes with Mutations Associated with Breast Cancer |
|  | IGF1R/AKT Signaling in Breast Cancer |
|  | ESR1/ERBB Positive Luminal Breast Cancer |
|  | Proteins Involved in Breast Cancer Related to IGF1R/Akt Signaling Pathway |
|  | Breast Cancer |
|  | ESR1 Signaling in Breast Cancer |
|  | Endometrial Cancer |
|  | Endometrioid Endometrial Cancer |
|  | Proteins Involved in Endometrial Cancer |
|  | Clear Cell Endometrial Cancer and Papillary Serous Endometrial Cancer |
|  | Genes with Mutations Associated with Endometrial Cancer |
|  | Proteins Involved in Endometriosis |
|  | Proteins Involved in Spontaneous Abortion |
|  | Genes with Mutations Associated with Hereditary Breast and/or Ovarian Cancer Syndrome |
|  | Proteins Involved in High-Grade Serous Ovarian Carcinoma |
|  | Endometrioid Ovarian Carcinoma |
|  | Proteins Involved in Endometrioid Ovarian Carcinoma |
|  | Genes with Mutations Associated with Ovarian Cancer |
|  | Clear Cell Ovarian Carcinoma |
|  | Proteins Involved in Mucinous Ovarian Carcinoma |
|  | High-Grade Serous Ovarian Carcinoma |
|  | Low-Grade Serous Ovarian Carcinoma |
|  | Proteins Involved in Low-Grade Serous Ovarian Carcinoma |
|  | Ovarian Cancer |
|  | Proteins Involved in Clear Cell Ovarian Carcinoma |
|  | Mucinous Ovarian Carcinoma |
|  | HPV E6 and E7 Induced Block of Apoptosis in Keratinocytes |
|  | Proteins Involved in HPV Infection |
|  | HPV Infection and Cancer |
|  | HPV E6 and E7 Induced Disruption of TNFR/NF-kB Signaling in Keratinocytes |
|  | Androgen Receptor/Akt Signaling |
|  | Androgen Receptor/NKX3-1 Signaling |
|  | Prostate Cancer |
|  | RNase L Signaling in Prostate Cancer |
|  | Proteins Involved in Prostate Cancer |
|  | Proteins Involved in Age-Related Macular Degeneration |
|  | CAV1 Dependent Senescence of Lung Fibroblasts |
|  | Alveolar Epithelial Cell Dysfunction in COPD |
|  | Ammonia Effects on Brain Cells |
|  | Dopamine/Gs Expression Targets |
|  | Dopamine/Gi Expression Targets |
|  | BMP2/BMPR2 Expression Targets |
|  | BMP4/BMPR2 Expression Targets |
|  | BMP7/BMPR2/ACVR2 Expression Targets |
|  | Sialophorin -> CTNNB/MYC/TP53 Signaling |
|  | Elevated Receptors -> Expression Targets in Aorta |
|  | CCL3 Expression Targets |
|  | CCL7 Expression Target |
|  | IL8 Expression Targets |
|  | CSF2 -> STAT Expression Targets |
|  | CNTF Expression Targets |
|  | Tamoxifen Induced Endometrial Cancer |
|  | Dexamethasone Induced Neurotoxicity |
|  | Dexamethasone Induced Diabetes |
|  | Dexamethasone Induced Osteoporosis |
|  | EGF -> CTNN Expression Targets |
|  | EGF -> AP-1/ATF Expression Targets |
|  | EGF -> CREB/CREBBP/ELK/SRF/MYC Expression Targets |
|  | EGF -> MEF/MYOD/NFATC Expression Targets |
|  | EGF -> TP53 Expression Targets |
|  | EGF -> STAT Expression Targets |
|  | EGFR/ERBB2 -> TP53 Signaling |
|  | F2 -> AP-1/CREB/ELK/SRF/SP1 Expression Targets |
|  | F2 -> STAT1/NF-kB Expression Targets |
|  | Glutamate/Gq Expression Targets |
|  | GH1/GHR -> NF-kB/ELK/SRF/MYC Expression Targets |
|  | GH1/GHR -> STAT Expression Targets |
|  | GH1/PRLR Expression Targets |
|  | HBEGF -> TP53 Expression Targets |
|  | TAM Receptor Signaling Activation in Cancer |
|  | PI3K/AKT/MTOR Signaling Activation in Cancer |
|  | DREAM Complex and FOXM1/MYBL2 Promote Cell Cycle Progression in Cancer |
|  | Proteins with Altered Expression in Cancer-Associated Sustaining of Proliferative Signaling |
|  | Genes with Mutations in Cancer-Associated Sustaining of Proliferative Signaling |
|  | RAS/RAF/MAPK Signaling Activation in Cancer |
|  | DNA Damage Checkpoint Impairment in Cancer |
|  | RAS/RAF/MAPK Signaling Activation by Blocking of Tumor Suppressors |
|  | PI3K/AKT/MTOR Signaling Activation by Blocking of Tumor Suppressors |
|  | Apoptosis Block by Inhibitor of Apoptosis Proteins (IAPs) in Cancer |
|  | Proteins with Altered Expression in Cancer-Associated Resisting to Cell Death |
|  | Genes with Mutations in Cancer-Associated Resisting to Cell Death |
|  | Suppression of Caspase Activity in Cancer |
|  | Apoptosis Evasion in Cancer: Overview |
|  | Hypoxia-Induced Cell Death Evasion in Cancer |
|  | Apoptosis Block by BCL2 Family Proteins in Cancer |
|  | TERT Activation in Cancer |
|  | Alternative Lengthening of Telomeres in Cancer |
|  | Genes with Mutations in Cancer-Associated Enabling of Replicative Immortality |
|  | Proteins with Altered Expression in Cancer-Associated Enabling of Replicative Immortality |
|  | VEGFA Dependent Angiogenesis in Cancer |
|  | Inhibition of THBS1 Activates Angiogenesis in Cancer |
|  | Metabolic Reprogramming in Cancer: Overview |
|  | Respiratory Chain Impairment in Cancer |
|  | Metabolic Effects of Oncogenes and Tumor Suppressor in Cancer Cells |
|  | Glutamine in Cancer Metabolism |
|  | Glycolysis Activation in Cancer (Warburg Effect) |
|  | Genes with Mutations in Cancer Metabolic Reprogramming |
|  | Proteins with Altered Expression in Cancer Metabolic Reprogramming |
|  | IGF1 -> MEF/MYOD/MYOG Expression Targets |
|  | IGF1 -> STAT Expression Targets |
|  | IGF1 -> ELK/SRF/HIF1A/MYC/SREBF Expression Targets |
|  | INHBA/ACVR2/BMPR Expression Targets |
|  | Fibronectin Expression Targets |
|  | IFNG/IFNR Expression Targets |
|  | IL11 Expression Targets |
|  | IL4 Expression Targets |
|  | Leptin -> STAT Expression Targets |
|  | Leptin -> ELK/SRF Expression Targets |
|  | Morphine Expression Targets |
|  | CD40 -> Expression Targets in Thymus |
|  | CD40 -> Expression Targets in Lymph Node |
|  | NRG1 -> CREB/CREBBP/ELK/SRF/MYC Expression Targets |
|  | NRG1 -> TP53 Expression Target |
|  | NRG1/Catenin Expression Targets |
|  | NRG1 -> AP-1/ATF Expression Targets |
|  | NGF -> AP-1/TP53/MYC Expression Targets |
|  | BDNF Expression Targets |
|  | NTF3 Expression Targets |
|  | OSM/OSMR Expression Targets |
|  | PDGF -> AP-1/CREB/CREBBP/MYC Expression Targets |
|  | PDGF -> STAT Expression Targets |
|  | AGT -> TP53 Expression Targets |
|  | SST Expression Targets |
|  | IFNA1/Gq Expression Targets |
|  | GUCYC2 Signaling |
|  | NTRK -> AP-1/CREB/ELK/SRF/MYC/SMAD3/TP53 Signaling |
|  | TGFBR -> ATF/GADD/MAX/TP53 Signaling |
|  | MSTN/ACVR2/BMPR Expression Targets |
|  | JNK/MAPK Signaling |
|  | P38 MAPK/MAPK14 Signaling |
|  | ERK/MAPK Canonical Signaling |
|  | TGFA -> TP53 Expression Targets |
|  | TGFB3-TGFBR2 Expression Targets |
|  | TGFB2-TGFBR2 Expression Targets |
|  | TGFB1-TGFBR2 Expression Targets |
|  | TGFB1-TGFBR1/AP-1 Expression Targets |
|  | TGFB1-ACVRL1 Expression Targets |
|  | FASLG Expression Targets |
|  | TNFSF10 Expression Targets |
|  | LTA Expression Targets |
|  | TNFSF14 Expression Targets |
|  | CD72 -> AP-1 Expression Targets |
|  | CD40LG -> STAT Expression Targets |
|  | TNFSF13 Expression Targets |
|  | TNFSF13B Expression Targets |
|  | TNF -> TP53/ATF Expression Targets |
|  | TNFR -> AP-1/ATF/TP53 Signaling |
|  | NGFR -> AP-1/CEBPB/CREB/ELK/SRF/TP53 Signaling |
|  | TNFRSF1A -> AP-1/ATF/TP53 Signaling |
|  | TLR4 -> AP-1/EGR1/HIF1A Expression Targets |
|  | Elevated Receptors -> Expression Targets in Kidney |
|  | PLAU -> STAT1 Expression Targets |
|  | PLAU -> ELK-SRF/AP-1 Expression Targets |
|  | WNT5A Expression Targets |
|  | sars-cov no binding |
|  | New Pathway (1) |
|  | H2A-K119 and H3-K27 |
|  | BCL6-TP53-BCL2 |
|  | New Pathway |
|  | Possible Interplay between SOX11, CCND1 and EZH2 in Mantle Cell Lymphoma |
|  | Figure 3\_Mixed network of dexamethasone targets |
|  | Figure 3\_Mixed network of dexamethasone targets |
|  | Overview. Diseases of the digestive system (chapter 10) |
|  | Overview. Diseases of the genitourinary system (chapter 13) |
|  | 2\_Interleukin-17 and interleukin-22 signaling in psoriasis |
|  | 4\_1\_Polymorphisms associated with inflammatory bowel diseases |
|  | 6\_p12I Viral protein promotes T-cell activation in HTLV-1 infection |
|  | 4\_Human papilloma virus E6 and E7 proteins promote epithelial cell survival |
|  | 2\_Mutations in SOD1 and associated proteins cause motor neuron death in familial ALS |
|  | DIPG non histone mutations |
|  | New Pathway (1) |
|  | DIPG overview\_2 |
|  | mapk1 |
|  | New Pathway |
|  | Interleukin-17 and interleukin-22 signaling in psoriasis |

---

|  |  |
| --- | --- |
| Group | Genes with Mutations Associated with Ageing (Mammalian Animal Model) |
|  | Genes with Mutations Associated with Melanoma |
|  | Genes with Mutations Associated with Psoriasis |
|  | Genes Associated with Systemic Lupus Erythematosus |
|  | Genes with Mutations Associated with Colorectal Cancer |
|  | Genes Associated with Ulcerative Colitis |
|  | Genes with Mutations Associated with Acute Myeloid Leukemia |
|  | Genes with Mutations Associated with B-Cell Acute Lymphoblastic Leukemia |
|  | Proteins Involved in B-Cell Acute Lymphoblastic Leukemia |
|  | Genes with Mutations Associated with B-Cell Chronic Lymphocytic Leukemia |
|  | Genes with Mutations Associated with Chronic Myeloid Leukemia |
|  | Genes with Mutations Associated with Diffuse Large-B-Cell Lymphoma, GCB Subtype |
|  | Genes with Mutations Associated with Follicular Lymphoma |
|  | Genes with Mutations Associated with Hodgkin Lymphoma |
|  | Genes with Mutations Associated with Mantle Cell Lymphoma |
|  | Genes with Mutations Associated with Mucosa Lymphoid Tissue (MALT) Lymphoma |
|  | Genes with Mutations Associated with Splenic Marginal Zone Lymphoma |
|  | Genes with Mutations Associated with Multiple Myeloma |
|  | Genes with Mutations Associated with Hepatocellular Carcinoma |
|  | Genes with Mutations Associated with Pancreatic Cancer |
|  | Proteins Involved in Hereditary Breast and Ovarian Cancer Syndrome |
|  | Proteins Involved in Cowden Syndrome |
|  | Genes with Mutations Associated with Li-Fraumeni Syndrome |
|  | Proteins Involved in Li-Fraumeni Syndrome |
|  | Genes with Mutations Associated with Primary Glioblastoma |
|  | Genes with Mutations Associated with Secondary Glioblastoma |
|  | Genes with Mutations Associated with Astrocytoma |
|  | Genes with Mutations Associated with Glioma |
|  | Genes with Mutations Associated with Medulloblastoma |
|  | Genes with Mutations Associated with Neuroblastoma |
|  | Genes with Mutations Associated with Breast Cancer |
|  | Genes with Mutations Associated with Endometrial Cancer |
|  | Genes with Mutations Associated with Endometriosis |
|  | Genes with Mutations Associated with Ovarian Cancer |
|  | Genes with Mutations Associated with Prostate Cancer |
|  | Proteins Related to Alternative Lengthening of Telomeres in Cancer |
|  | other partners for SARS |

---

|  |  |
| --- | --- |
| Source | curated |
|  | ResNet |
|  | GO component |

---

|  |  |
| --- | --- |
| MedScan ID | 7157 |

---

|  |  |
| --- | --- |
| LocusLink ID | 7157 |
|  | 22059 |
|  | 24842 |
|  | 281542 |
|  | 281540 |
|  | 30590 |
|  | 30535 |
|  | 321993 |
|  | 301300 |
|  | 224883 |
|  | 289761 |

---

|  |  |
| --- | --- |
| Alias | FLJ92943 |
|  | P53 |
|  | antigen NY-CO-13 |
|  | TP53 |
|  | tumor protein p53 (Li-Fraumeni syndrome) |
|  | p53 antigen |
|  | cys51stop |
|  | OTTMUSP00000006194 |
|  | tumor suppressor p53 |
|  | tumor protein p53 |
|  | MGC112612 |
|  | LOC146749 |
|  | p53 alternative splice isoform p35/HAS |
|  | mutant tumor protein 53 |
|  | transformation related protein 53 |
|  | RP23-56I20.1 |
|  | p53 transformation suppressor |
|  | tumor protein 53 |
|  | LOC201237 |
|  | p53 tumor suppressor |
|  | P53\_HUMAN |
|  | lfs1 |
|  | TRP53 |
|  | BCC7 |
|  | p53 cellular tumor antigen |
|  | cellular tumor antigen p53 |
|  | BMFS5 |
|  | phosphoprotein p53 |
|  | transformation-related protein 53 |
|  | tumor supressor p53 |
|  | bbl |
|  | bfy |
|  | bhy |
|  | p44 |
|  | mutant p53 |
|  | TRP53 gene |
|  | tumour protein 53 |
|  | p53 tumour suppressor |
|  | p53 cellular tumour antigen |
|  | cellular tumour antigen p53 |
|  | tumour protein p53 (Li-Fraumeni syndrome) |
|  | tumour suppressor p53 |
|  | tumour supressor p53 |
|  | tumour protein p53 |
|  | mutant tumour protein 53 |
|  | TRP53 protein |
|  | OTTHUMP00000221340 |
|  | tumor suppressor (p53) |
|  | TRP53 (p53) |
|  | P-53 |
|  | tumor suppressor |
|  | similar to transformation related protein 53 |
|  | scaffold |
|  | Tp53 protein |
|  | chromosome |
|  | similar to transformation related protein 53 [Mus musculus] |
|  | drp53 |
|  | zgc:111919 |
|  | similar to p53 alternative splice isoform p35/HAS [Rattus norvegicus] |
|  | tumor suppressor homolog p53 |
|  | similar to Cellular tumor antigen p53 (Tumor suppressor p53) |
|  | etID22686.5 |
|  | (P53) |
|  | brp53 |
|  | fb40d06 |
|  | wu:fb40d06 |
|  | similar to p53 alternative splice isoform p35/HAS |
|  | ME-delta123p53 |

---

|  |  |
| --- | --- |
| GO ID | 0005524 |
|  | 0003677 |
|  | 0001228 |
|  | 0003700 |
|  | 0000981 |
|  | 0097371 |
|  | 0000980 |
|  | 0000978 |
|  | 0001085 |
|  | 0001094 |
|  | 0051087 |
|  | 0003682 |
|  | 0005507 |
|  | 0001046 |
|  | 0097718 |
|  | 0019899 |
|  | 0035035 |
|  | 0042826 |
|  | 0035033 |
|  | 0003730 |
|  | 0002039 |
|  | 1990841 |
|  | 0002020 |
|  | 0047485 |
|  | 0046982 |
|  | 0042803 |
|  | 0019901 |
|  | 0051721 |
|  | 0019903 |
|  | 0043621 |
|  | 0030971 |
|  | 0008134 |
|  | 0044212 |
|  | 0031625 |
|  | 0008270 |
|  | 0002326 |
|  | 0030330 |
|  | 0006977 |
|  | 0006978 |
|  | 0000733 |
|  | 0006983 |
|  | 0051123 |
|  | 0007265 |
|  | 0033077 |
|  | 0002360 |
|  | 0002309 |
|  | 0006914 |
|  | 0006284 |
|  | 0048539 |
|  | 0060411 |
|  | 0007569 |
|  | 0007050 |
|  | 0030154 |
|  | 0008283 |
|  | 0006974 |
|  | 0034644 |
|  | 0071494 |
|  | 0072717 |
|  | 0035690 |
|  | 0071480 |
|  | 0042149 |
|  | 0071456 |
|  | 0071479 |
|  | 0021549 |
|  | 0031497 |
|  | 0048512 |
|  | 0019221 |
|  | 0008340 |
|  | 0006302 |
|  | 0048568 |
|  | 0043153 |
|  | 0007369 |
|  | 0002244 |
|  | 0060218 |
|  | 0001701 |
|  | 0060333 |
|  | 0097193 |
|  | 0072332 |
|  | 0042771 |
|  | 0070059 |
|  | 1990144 |
|  | 0009299 |
|  | 0043504 |
|  | 0031571 |
|  | 0071850 |
|  | 0007275 |
|  | 0035264 |
|  | 0070266 |
|  | 0008156 |
|  | 0043066 |
|  | 0030308 |
|  | 0008285 |
|  | 0048147 |
|  | 1904024 |
|  | 0051097 |
|  | 1901525 |
|  | 0007406 |
|  | 1903799 |
|  | 0045861 |
|  | 2000378 |
|  | 0051974 |
|  | 0000122 |
|  | 0045892 |
|  | 0030512 |
|  | 0051402 |
|  | 0006289 |
|  | 0097252 |
|  | 0090403 |
|  | 0045899 |
|  | 0043065 |
|  | 0010666 |
|  | 0090343 |
|  | 0071158 |
|  | 1900119 |
|  | 0010628 |
|  | 0031065 |
|  | 2001244 |
|  | 0035794 |
|  | 0043525 |
|  | 0050731 |
|  | 1902895 |
|  | 1903800 |
|  | 0046827 |
|  | 1900740 |
|  | 0032461 |
|  | 2000379 |
|  | 0090200 |
|  | 0070245 |
|  | 0045944 |
|  | 1990440 |
|  | 0061419 |
|  | 0045893 |
|  | 0016579 |
|  | 0051289 |
|  | 0006606 |
|  | 0008104 |
|  | 0050821 |
|  | 0051262 |
|  | 0065003 |
|  | 0009303 |
|  | 0043516 |
|  | 0042981 |
|  | 1902749 |
|  | 2000772 |
|  | 2000269 |
|  | 0072363 |
|  | 1902253 |
|  | 0046902 |
|  | 1902108 |
|  | 1901796 |
|  | 0034103 |
|  | 0006355 |
|  | 0001836 |
|  | 0090399 |
|  | 0010165 |
|  | 0046677 |
|  | 0010332 |
|  | 0002931 |
|  | 0009651 |
|  | 0072331 |
|  | 0001756 |
|  | 0007179 |
|  | 0016032 |
|  | 0016605 |
|  | 0005737 |
|  | 0005829 |
|  | 0005783 |
|  | 0005759 |
|  | 0005739 |
|  | 0016604 |
|  | 0000790 |
|  | 0016363 |
|  | 0005730 |
|  | 0005654 |
|  | 0005634 |
|  | 0032991 |
|  | 0005657 |
|  | 0035861 |
|  | 0005669 |
|  | 0005667 |
|  | 0000977 |
|  | 0046872 |
|  | 0008022 |
|  | 0043565 |
|  | 0001221 |
|  | 0006915 |
|  | 0007049 |
|  | 0034613 |
|  | 0034614 |
|  | 0007417 |
|  | 0051276 |
|  | 0007623 |
|  | 0009792 |
|  | 0007507 |
|  | 2000279 |
|  | 0010629 |
|  | 0045930 |
|  | 0048662 |
|  | 0045787 |
|  | 0002687 |
|  | 0036003 |
|  | 0012501 |
|  | 0051726 |
|  | 0042127 |
|  | 1903205 |
|  | 0051453 |
|  | 0043523 |
|  | 0070243 |
|  | 0006357 |
|  | 0009411 |
|  | 0042493 |
|  | 0006979 |
|  | 0048511 |
|  | 0000785 |
|  | 0007568 |
|  | 0034605 |
|  | 0071417 |
|  | 2000871 |
|  | 1905587 |
|  | 0090276 |
|  | 0032306 |
|  | 0010224 |
|  | 0043200 |
|  | 0031000 |
|  | 0034097 |
|  | 0045471 |
|  | 0055093 |
|  | 0010035 |
|  | 0010038 |
|  | 0014070 |
|  | 0010243 |
|  | 0032526 |
|  | 0033552 |
|  | 0042060 |
|  | 0016925 |
|  | 0031052 |
|  | 0019048 |
|  | 0001077 |
|  | 0001074 |
|  | 0005678 |
|  | 0042221 |
|  | 0000075 |
|  | 0033554 |
|  | 0000990 |
|  | 0042802 |
|  | 0005622 |
|  | 0050790 |
|  | 0000979 |
|  | 0003684 |
|  | 0000060 |
|  | 0005515 |
|  | 0003690 |
|  | 0043234 |
|  | 0007596 |
|  | 0006917 |
|  | 0006351 |
|  | 0007219 |
|  | 0006366 |
|  | 0043161 |
|  | 0006461 |
|  | 0000739 |
|  | 0010843 |
|  | 0016566 |
|  | 0016563 |
|  | 0016564 |
|  | 0008635 |
|  | 0008629 |
|  | 0044419 |
|  | 0010553 |
|  | 0010552 |
|  | 0005626 |
|  | 0005625 |
|  | 0003702 |
|  | 0070215 |
|  | 0002347 |
|  | 0016481 |
|  | 0045941 |
|  | 0045449 |
|  | 0006350 |
|  | 0004518 |
|  | 0018144 |
|  | 0045786 |
|  | 0008628 |
|  | 0008630 |
|  | 0000074 |
|  | 0006310 |
|  | 0000077 |
|  | 0006353 |
|  | 0006829 |
|  | 0030528 |
|  | 0031564 |
|  | 0043280 |
|  | 0051597 |
|  | 0000232 |
|  | 0008151 |
|  | 0007048 |
|  | 0045182 |
|  | 0008181 |
|  | 0006281 |
|  | 1990248 |
|  | 0031965 |
|  | 0033209 |
|  | 0090575 |
|  | 0000976 |
|  | 0005856 |
|  | 0001102 |
|  | 1905856 |
|  | 0005813 |

---

|  |  |
| --- | --- |
| KEGG ID | mmu:22059 |
|  | rno:24842 |
|  | hsa:7157 |

---

|  |  |
| --- | --- |
| MedLine Reference | 10684946 |
|  | 11502571 |
|  | 11606748 |
|  | 11773703 |
|  | 11827957 |
|  | 11958430 |
|  | 11973333 |
|  | 12143041 |
|  | 12163498 |
|  | 12215673 |
|  | 12464630 |
|  | 12477932 |
|  | 12503078 |
|  | 12506145 |
|  | 12527914 |
|  | 12534344 |
|  | 12538352 |
|  | 12549819 |
|  | 12654005 |
|  | 12835324 |
|  | 12850271 |
|  | 12869365 |
|  | 14514686 |
|  | 14576152 |
|  | 14685795 |
|  | 14695767 |
|  | 14739302 |
|  | 14740296 |
|  | 14962081 |
|  | 15002037 |
|  | 15033826 |
|  | 15122989 |
|  | 15199126 |
|  | 15219885 |
|  | 15272143 |
|  | 15315938 |
|  | 15735701 |
|  | 15829407 |
|  | 15872011 |
|  | 15922606 |
|  | 15990448 |
|  | 16000635 |
|  | 16005638 |
|  | 16118209 |
|  | 16207712 |
|  | 16282427 |
|  | 16392041 |
|  | 16427076 |
|  | 16491117 |
|  | 16614397 |
|  | 16688778 |
|  | 16690610 |
|  | 16729028 |
|  | 16760664 |
|  | 16870742 |
|  | 16914427 |
|  | 16937528 |
|  | 17060322 |
|  | 3004941 |
|  | 3060862 |
|  | 8441680 |
|  | 9278438 |
|  | 10027415 |
|  | 10029060 |
|  | 10029411 |
|  | 10049357 |
|  | 10065150 |
|  | 10065151 |
|  | 10074901 |
|  | 10075839 |
|  | 10077639 |
|  | 10077642 |
|  | 10082576 |
|  | 10082662 |
|  | 10086392 |
|  | 10094833 |
|  | 10101799 |
|  | 10191063 |
|  | 10195427 |
|  | 10200460 |
|  | 10200522 |
|  | 10200544 |
|  | 10203277 |
|  | 10204804 |
|  | 10205143 |
|  | 10210642 |
|  | 10319859 |
|  | 10329616 |
|  | 10329696 |
|  | 10338216 |
|  | 10341706 |
|  | 10341708 |
|  | 10341712 |
|  | 10346812 |
|  | 10349636 |
|  | 10354500 |
|  | 10362261 |
|  | 10367167 |
|  | 10374394 |
|  | 10380877 |
|  | 10393064 |
|  | 10403253 |
|  | 10419603 |
|  | 10428027 |
|  | 10431243 |
|  | 10435582 |
|  | 10435642 |
|  | 10436023 |
|  | 10436056 |
|  | 10438933 |
|  | 10458762 |
|  | 10464378 |
|  | 10465341 |
|  | 10478963 |
|  | 10481009 |
|  | 10484066 |
|  | 10490643 |
|  | 10498884 |
|  | 10498887 |
|  | 10501974 |
|  | 10502405 |
|  | 10508513 |
|  | 10508527 |
|  | 10514395 |
|  | 10517501 |
|  | 10517975 |
|  | 10523829 |
|  | 10525351 |
|  | 10528631 |
|  | 10528916 |
|  | 10529736 |
|  | 10541552 |
|  | 10541553 |
|  | 10541808 |
|  | 10559790 |
|  | 10567591 |
|  | 10590213 |
|  | 10591652 |
|  | 10591653 |
|  | 10597255 |
|  | 10602475 |
|  | 10602497 |
|  | 10611241 |
|  | 10611243 |
|  | 10619357 |
|  | 10620525 |
|  | 10620618 |
|  | 10623800 |
|  | 10644554 |
|  | 10646856 |
|  | 10660095 |
|  | 10687094 |
|  | 10694747 |
|  | 10696769 |
|  | 10706103 |
|  | 10713689 |
|  | 10716720 |
|  | 10728678 |
|  | 10739657 |
|  | 10749125 |
|  | 10749126 |
|  | 10754563 |
|  | 10757812 |
|  | 10760284 |
|  | 10760784 |
|  | 10761921 |
|  | 10779360 |
|  | 10783170 |
|  | 10786799 |
|  | 10802220 |
|  | 10804183 |
|  | 10805721 |
|  | 10805794 |
|  | 10805805 |
|  | 10815637 |
|  | 10823907 |
|  | 10828268 |
|  | 10834928 |
|  | 10845423 |
|  | 10861008 |
|  | 10863098 |
|  | 10900466 |
|  | 10910287 |
|  | 10911993 |
|  | 10919675 |
|  | 10930038 |
|  | 10931486 |
|  | 10934169 |
|  | 10945472 |
|  | 10949306 |
|  | 10949934 |
|  | 10956433 |
|  | 10964497 |
|  | 10973245 |
|  | 10973261 |
|  | 10987303 |
|  | 10987662 |
|  | 10993916 |
|  | 10993919 |
|  | 10995391 |
|  | 11003672 |
|  | 11005771 |
|  | 11016618 |
|  | 11023548 |
|  | 11024475 |
|  | 11035798 |
|  | 11040211 |
|  | 11042159 |
|  | 11050010 |
|  | 11076674 |
|  | 11076861 |
|  | 11086988 |
|  | 11092819 |
|  | 11095619 |
|  | 11103934 |
|  | 11106258 |
|  | 11106570 |
|  | 11106587 |
|  | 11106750 |
|  | 11146559 |
|  | 11150333 |
|  | 11159746 |
|  | 11159747 |
|  | 11159909 |
|  | 11160722 |
|  | 11169961 |
|  | 11170239 |
|  | 11172041 |
|  | 11196181 |
|  | 11212243 |
|  | 11212265 |
|  | 11217851 |
|  | 11238917 |
|  | 11238948 |
|  | 11246230 |
|  | 11280729 |
|  | 11285190 |
|  | 11289103 |
|  | 11295231 |
|  | 11313959 |
|  | 11313961 |
|  | 11319615 |
|  | 11323179 |
|  | 11323676 |
|  | 11326422 |
|  | 11356029 |
|  | 11358863 |
|  | 11359911 |
|  | 11373269 |
|  | 11376694 |
|  | 11385732 |
|  | 11387238 |
|  | 11395365 |
|  | 11415935 |
|  | 11423996 |
|  | 11431698 |
|  | 11432840 |
|  | 11438662 |
|  | 11439219 |
|  | 11481437 |
|  | 11486039 |
|  | 11517258 |
|  | 11522642 |
|  | 11522657 |
|  | 11528400 |
|  | 11532341 |
|  | 11532857 |
|  | 11536039 |
|  | 11544182 |
|  | 11559812 |
|  | 11567994 |
|  | 11571646 |
|  | 11581300 |
|  | 11599922 |
|  | 11600127 |
|  | 11675005 |
|  | 11684435 |
|  | 11694875 |
|  | 11695547 |
|  | 11695551 |
|  | 11695559 |
|  | 11695560 |
|  | 11696456 |
|  | 11697899 |
|  | 11708657 |
|  | 11709723 |
|  | 11718223 |
|  | 11719445 |
|  | 11726500 |
|  | 11739563 |
|  | 11749692 |
|  | 11756558 |
|  | 11771658 |
|  | 11779501 |
|  | 11780111 |
|  | 11780924 |
|  | 11781307 |
|  | 11781827 |
|  | 11786406 |
|  | 11788578 |
|  | 11790307 |
|  | 11799106 |
|  | 11810634 |
|  | 11815254 |
|  | 11818961 |
|  | 11825686 |
|  | 11832348 |
|  | 11837328 |
|  | 11850820 |
|  | 11856728 |
|  | 11864976 |
|  | 11865195 |
|  | 11867524 |
|  | 11869471 |
|  | 11884140 |
|  | 11884608 |
|  | 11888671 |
|  | 11890468 |
|  | 11891301 |
|  | 11895858 |
|  | 11896287 |
|  | 11896625 |
|  | 11900416 |
|  | 11900485 |
|  | 11904448 |
|  | 11906209 |
|  | 11909939 |
|  | 11914272 |
|  | 11914273 |
|  | 11921187 |
|  | 11924798 |
|  | 11931974 |
|  | 11932750 |
|  | 11940651 |
|  | 11943780 |
|  | 11948180 |
|  | 11953423 |
|  | 11956084 |
|  | 11956239 |
|  | 11959917 |
|  | 11960905 |
|  | 11967151 |
|  | 11967210 |
|  | 11970883 |
|  | 11971958 |
|  | 11971968 |
|  | 11971980 |
|  | 11980714 |
|  | 11983168 |
|  | 11996974 |
|  | 12000789 |
|  | 12006490 |
|  | 12015983 |
|  | 12019151 |
|  | 12032820 |
|  | 12032821 |
|  | 12032825 |
|  | 12034505 |
|  | 12034755 |
|  | 12036908 |
|  | 12036937 |
|  | 12036945 |
|  | 12037687 |
|  | 12065413 |
|  | 12065679 |
|  | 12067213 |
|  | 12071805 |
|  | 12082025 |
|  | 12082535 |
|  | 12082536 |
|  | 12086865 |
|  | 12086888 |
|  | 12094020 |
|  | 12094075 |
|  | 12096336 |
|  | 12096340 |
|  | 12110179 |
|  | 12135983 |
|  | 12140760 |
|  | 12140762 |
|  | 12141446 |
|  | 12145208 |
|  | 12151310 |
|  | 12162806 |
|  | 12167164 |
|  | 12185594 |
|  | 12186934 |
|  | 12189199 |
|  | 12191858 |
|  | 12192050 |
|  | 12202832 |
|  | 12208847 |
|  | 12214254 |
|  | 12214270 |
|  | 12217864 |
|  | 12218099 |
|  | 12232053 |
|  | 12232794 |
|  | 12235107 |
|  | 12242304 |
|  | 12243750 |
|  | 12356735 |
|  | 12376467 |
|  | 12386807 |
|  | 12388812 |
|  | 12393587 |
|  | 12393879 |
|  | 12397361 |
|  | 12397362 |
|  | 12402042 |
|  | 12413886 |
|  | 12414647 |
|  | 12417719 |
|  | 12426394 |
|  | 12432262 |
|  | 12438222 |
|  | 12438257 |
|  | 12466851 |
|  | 12467136 |
|  | 12486224 |
|  | 12492400 |
|  | 12493444 |
|  | 12503075 |
|  | 12505991 |
|  | 12507917 |
|  | 12509446 |
|  | 12512872 |
|  | 12517772 |
|  | 12517796 |
|  | 12517797 |
|  | 12519204 |
|  | 12524542 |
|  | 12533509 |
|  | 12540173 |
|  | 12540848 |
|  | 12547516 |
|  | 12547998 |
|  | 12551891 |
|  | 12553907 |
|  | 12554679 |
|  | 12555066 |
|  | 12555069 |
|  | 12565905 |
|  | 12567186 |
|  | 12578823 |
|  | 12588846 |
|  | 12591728 |
|  | 12592396 |
|  | 12600942 |
|  | 12606380 |
|  | 12606552 |
|  | 12628936 |
|  | 12629205 |
|  | 12642502 |
|  | 12667443 |
|  | 12670909 |
|  | 12674199 |
|  | 12684687 |
|  | 12702868 |
|  | 12706858 |
|  | 12716304 |
|  | 12717433 |
|  | 12719721 |
|  | 12725522 |
|  | 12727849 |
|  | 12734185 |
|  | 12743811 |
|  | 12750252 |
|  | 12750388 |
|  | 12752260 |
|  | 12756225 |
|  | 12760067 |
|  | 12761499 |
|  | 12761582 |
|  | 12771941 |
|  | 12773518 |
|  | 12776191 |
|  | 12782018 |
|  | 12782574 |
|  | 12786980 |
|  | 12793759 |
|  | 12793760 |
|  | 12810724 |
|  | 12813031 |
|  | 12817992 |
|  | 12826669 |
|  | 12829028 |
|  | 12832489 |
|  | 12833065 |
|  | 12839925 |
|  | 12842843 |
|  | 12855557 |
|  | 12872134 |
|  | 12878730 |
|  | 12881708 |
|  | 12883670 |
|  | 12891704 |
|  | 12893686 |
|  | 12897130 |
|  | 12907595 |
|  | 12907637 |
|  | 12909629 |
|  | 12909720 |
|  | 12914700 |
|  | 12914701 |
|  | 12915712 |
|  | 12944468 |
|  | 12944479 |
|  | 12952892 |
|  | 12955074 |
|  | 12955085 |
|  | 12972599 |
|  | 1347472 |
|  | 1370652 |
|  | 1377062 |
|  | 1409609 |
|  | 1448088 |
|  | 14500365 |
|  | 14500377 |
|  | 14500378 |
|  | 14500851 |
|  | 14505568 |
|  | 14506698 |
|  | 14512017 |
|  | 14516774 |
|  | 14517281 |
|  | 14522252 |
|  | 14550500 |
|  | 14555661 |
|  | 14559792 |
|  | 14562055 |
|  | 14569010 |
|  | 14570892 |
|  | 14578172 |
|  | 14586409 |
|  | 14599776 |
|  | 14602726 |
|  | 14607778 |
|  | 14610316 |
|  | 14614825 |
|  | 14627978 |
|  | 14633666 |
|  | 14634213 |
|  | 14635196 |
|  | 14645510 |
|  | 14645705 |
|  | 14646229 |
|  | 14647411 |
|  | 14647424 |
|  | 14654789 |
|  | 14662893 |
|  | 14673154 |
|  | 14690604 |
|  | 14695178 |
|  | 14702042 |
|  | 14706332 |
|  | 14706337 |
|  | 14706339 |
|  | 14706459 |
|  | 14707049 |
|  | 14707288 |
|  | 14713961 |
|  | 14719112 |
|  | 14729462 |
|  | 14729946 |
|  | 14732024 |
|  | 14737103 |
|  | 14737108 |
|  | 14741382 |
|  | 14744772 |
|  | 14744934 |
|  | 14747529 |
|  | 14749764 |
|  | 14752107 |
|  | 14755249 |
|  | 14764457 |
|  | 14767071 |
|  | 14871929 |
|  | 14963330 |
|  | 14966529 |
|  | 14976551 |
|  | 14981542 |
|  | 14983023 |
|  | 14985507 |
|  | 14990790 |
|  | 14991602 |
|  | 14998493 |
|  | 15010310 |
|  | 15010313 |
|  | 15013777 |
|  | 15020477 |
|  | 15026124 |
|  | 15026330 |
|  | 15029243 |
|  | 15044451 |
|  | 15051493 |
|  | 15064735 |
|  | 15067031 |
|  | 15072823 |
|  | 15073170 |
|  | 15081359 |
|  | 15082782 |
|  | 15105378 |
|  | 15105421 |
|  | 15108799 |
|  | 15123655 |
|  | 15131084 |
|  | 15133494 |
|  | 15138611 |
|  | 15140942 |
|  | 15142950 |
|  | 15145929 |
|  | 15150107 |
|  | 15155458 |
|  | 15155568 |
|  | 15156563 |
|  | 15161027 |
|  | 15162501 |
|  | 15175242 |
|  | 15175260 |
|  | 15181534 |
|  | 15190215 |
|  | 15198801 |
|  | 15202010 |
|  | 15204965 |
|  | 15208669 |
|  | 15210712 |
|  | 15215324 |
|  | 15221010 |
|  | 15222780 |
|  | 15231717 |
|  | 15232580 |
|  | 15247009 |
|  | 15254240 |
|  | 15254344 |
|  | 15256442 |
|  | 15256477 |
|  | 15261144 |
|  | 15265983 |
|  | 15273287 |
|  | 15282321 |
|  | 15287091 |
|  | 15289314 |
|  | 15289317 |
|  | 15299027 |
|  | 15305061 |
|  | 15314658 |
|  | 15319471 |
|  | 15326486 |
|  | 15333746 |
|  | 15334065 |
|  | 15339983 |
|  | 15343266 |
|  | 1535557 |
|  | 15361844 |
|  | 15361846 |
|  | 15361884 |
|  | 15364958 |
|  | 15367607 |
|  | 15371270 |
|  | 15371510 |
|  | 15377654 |
|  | 15380514 |
|  | 15382083 |
|  | 15383401 |
|  | 15383658 |
|  | 15386426 |
|  | 15388331 |
|  | 15455033 |
|  | 15456863 |
|  | 15466171 |
|  | 15467370 |
|  | 15470641 |
|  | 15480417 |
|  | 15480423 |
|  | 15483136 |
|  | 15485914 |
|  | 15488761 |
|  | 15489287 |
|  | 15509522 |
|  | 15509711 |
|  | 15514675 |
|  | 15522206 |
|  | 15525786 |
|  | 15526030 |
|  | 1552940 |
|  | 15531911 |
|  | 15543560 |
|  | 15548714 |
|  | 15557088 |
|  | 15584024 |
|  | 15588938 |
|  | 15592418 |
|  | 15598651 |
|  | 15603759 |
|  | 15604409 |
|  | 15607980 |
|  | 15607981 |
|  | 15611068 |
|  | 15615595 |
|  | 15616590 |
|  | 15617832 |
|  | 15618518 |
|  | 15619621 |
|  | 15626733 |
|  | 15632067 |
|  | 15640352 |
|  | 15642165 |
|  | 15643668 |
|  | 15647757 |
|  | 15652750 |
|  | 15654339 |
|  | 15658891 |
|  | 15661885 |
|  | 15664192 |
|  | 15665282 |
|  | 15665293 |
|  | 15668238 |
|  | 15678128 |
|  | 15678149 |
|  | 15684377 |
|  | 15694382 |
|  | 15700305 |
|  | 15723042 |
|  | 1572638 |
|  | 15729694 |
|  | 15735665 |
|  | 15735681 |
|  | 15735683 |
|  | 15735727 |
|  | 15735758 |
|  | 15735763 |
|  | 15743813 |
|  | 15744335 |
|  | 15746445 |
|  | 15748640 |
|  | 15753076 |
|  | 15753354 |
|  | 15761494 |
|  | 15765918 |
|  | 15778864 |
|  | 15781618 |
|  | 15782199 |
|  | 15789337 |
|  | 15790496 |
|  | 15792350 |
|  | 1579500 |
|  | 15806156 |
|  | 15811878 |
|  | 15817608 |
|  | 15817611 |
|  | 15821748 |
|  | 15824740 |
|  | 15831478 |
|  | 15833886 |
|  | 15837625 |
|  | 15840768 |
|  | 15849741 |
|  | 15855329 |
|  | 15864314 |
|  | 15865931 |
|  | 15865938 |
|  | 15865944 |
|  | 15866171 |
|  | 15867343 |
|  | 15867355 |
|  | 15870277 |
|  | 15870706 |
|  | 15875769 |
|  | 15878778 |
|  | 15888345 |
|  | 15894267 |
|  | 15897901 |
|  | 15898111 |
|  | 15902970 |
|  | 15917304 |
|  | 15917654 |
|  | 15919904 |
|  | 15921859 |
|  | 15924142 |
|  | 15924572 |
|  | 15928081 |
|  | 15940247 |
|  | 15940268 |
|  | 15947793 |
|  | 15958535 |
|  | 15958557 |
|  | 15964796 |
|  | 15964995 |
|  | 15968270 |
|  | 15975959 |
|  | 15977174 |
|  | 15978002 |
|  | 15983031 |
|  | 15990362 |
|  | 15996546 |
|  | 16004968 |
|  | 16006521 |
|  | 16007073 |
|  | 16007135 |
|  | 16007165 |
|  | 16018998 |
|  | 16023597 |
|  | 16027727 |
|  | 16038709 |
|  | 16041388 |
|  | 16044147 |
|  | 16055552 |
|  | 16055726 |
|  | 16061649 |
|  | 16079796 |
|  | 16079837 |
|  | 16079851 |
|  | 16086332 |
|  | 16087272 |
|  | 16098148 |
|  | 16098465 |
|  | 16103190 |
|  | 16103874 |
|  | 16107615 |
|  | 16107691 |
|  | 16107692 |
|  | 16107724 |
|  | 16107883 |
|  | 16110744 |
|  | 16116187 |
|  | 16123778 |
|  | 16140739 |
|  | 16141072 |
|  | 16141073 |
|  | 16150539 |
|  | 16151013 |
|  | 16166291 |
|  | 16166651 |
|  | 16169021 |
|  | 16169464 |
|  | 16176931 |
|  | 16177209 |
|  | 16178126 |
|  | 16183742 |
|  | 16199867 |
|  | 16204064 |
|  | 16205640 |
|  | 16207734 |
|  | 16211273 |
|  | 16219768 |
|  | 16222300 |
|  | 16222716 |
|  | 16227443 |
|  | 16237043 |
|  | 16251190 |
|  | 16255829 |
|  | 16257849 |
|  | 16257854 |
|  | 16260494 |
|  | 16260620 |
|  | 16260621 |
|  | 16261166 |
|  | 16267022 |
|  | 16267035 |
|  | 16278673 |
|  | 16278683 |
|  | 16288012 |
|  | 16288016 |
|  | 1628822 |
|  | 16293626 |
|  | 16301525 |
|  | 16314418 |
|  | 16337934 |
|  | 1634762 |
|  | 16354690 |
|  | 16357136 |
|  | 16360031 |
|  | 16363065 |
|  | 16368933 |
|  | 16373391 |
|  | 16380437 |
|  | 16384933 |
|  | 1638548 |
|  | 16391561 |
|  | 16397217 |
|  | 16397225 |
|  | 16397237 |
|  | 16397267 |
|  | 16397624 |
|  | 16400328 |
|  | 16401638 |
|  | 16407291 |
|  | 16416089 |
|  | 16418478 |
|  | 1643308 |
|  | 16434961 |
|  | 16436596 |
|  | 16446310 |
|  | 16449526 |
|  | 16449974 |
|  | 16456545 |
|  | 16461747 |
|  | 16464479 |
|  | 16465443 |
|  | 16473278 |
|  | 16479172 |
|  | 16480340 |
|  | 16489021 |
|  | 16492743 |
|  | 16492744 |
|  | 16492765 |
|  | 16501611 |
|  | 16503212 |
|  | 16510568 |
|  | 16513254 |
|  | 16514420 |
|  | 16518872 |
|  | 16528385 |
|  | 16532027 |
|  | 16533502 |
|  | 16533949 |
|  | 16537910 |
|  | 16537918 |
|  | 16538233 |
|  | 16540640 |
|  | 16543248 |
|  | 16546138 |
|  | 16546942 |
|  | 16552004 |
|  | 16557586 |
|  | 16570054 |
|  | 16571598 |
|  | 16581768 |
|  | 16582589 |
|  | 16585167 |
|  | 16585505 |
|  | 16586494 |
|  | 16597604 |
|  | 16597646 |
|  | 16598767 |
|  | 16608880 |
|  | 16611991 |
|  | 16616333 |
|  | 16624822 |
|  | 16651430 |
|  | 16651519 |
|  | 16670198 |
|  | 16675955 |
|  | 16678112 |
|  | 16687486 |
|  | 16697957 |
|  | 16702401 |
|  | 16705170 |
|  | 16707443 |
|  | 16714381 |
|  | 16717092 |
|  | 16728594 |
|  | 16729027 |
|  | 16729035 |
|  | 16738336 |
|  | 16757565 |
|  | 16757976 |
|  | 16767157 |
|  | 16777961 |
|  | 16778087 |
|  | 16790550 |
|  | 16816378 |
|  | 16818635 |
|  | 16822983 |
|  | 16829633 |
|  | 16835303 |
|  | 16839876 |
|  | 16843262 |
|  | 16849540 |
|  | 16849561 |
|  | 16857722 |
|  | 16861914 |
|  | 1686571 |
|  | 16869739 |
|  | 16869758 |
|  | 16869764 |
|  | 16870721 |
|  | 16885345 |
|  | 16885364 |
|  | 16891316 |
|  | 16892556 |
|  | 16897187 |
|  | 16904610 |
|  | 16912162 |
|  | 16912279 |
|  | 16931520 |
|  | 16931621 |
|  | 16943427 |
|  | 16946709 |
|  | 16951207 |
|  | 16957739 |
|  | 16971940 |
|  | 16978870 |
|  | 16982732 |
|  | 16982747 |
|  | 17006319 |
|  | 17008321 |
|  | 17012228 |
|  | 17056717 |
|  | 17070800 |
|  | 17080083 |
|  | 17081294 |
|  | 17086174 |
|  | 17090686 |
|  | 17097565 |
|  | 17101136 |
|  | 17118206 |
|  | 17122775 |
|  | 17129780 |
|  | 17138902 |
|  | 17142669 |
|  | 17149979 |
|  | 17157790 |
|  | 17167174 |
|  | 17182091 |
|  | 17182869 |
|  | 17214514 |
|  | 17251932 |
|  | 17251933 |
|  | 1765007 |
|  | 1766668 |
|  | 1766680 |
|  | 1821855 |
|  | 1923526 |
|  | 1981050 |
|  | 2142762 |
|  | 2565878 |
|  | 2567264 |
|  | 2832726 |
|  | 2851124 |
|  | 3023970 |
|  | 3116273 |
|  | 3547084 |
|  | 6092064 |
|  | 6095116 |
|  | 6296874 |
|  | 6315396 |
|  | 6366521 |
|  | 6379601 |
|  | 6387444 |
|  | 6400059 |
|  | 6646235 |
|  | 7477326 |
|  | 7477327 |
|  | 7478622 |
|  | 7479059 |
|  | 7504233 |
|  | 7507509 |
|  | 7514570 |
|  | 7525358 |
|  | 7534899 |
|  | 7539482 |
|  | 7540599 |
|  | 7546219 |
|  | 7554030 |
|  | 7557882 |
|  | 7557995 |
|  | 7568153 |
|  | 7575496 |
|  | 7583151 |
|  | 7586156 |
|  | 7600529 |
|  | 7601457 |
|  | 7605578 |
|  | 7605579 |
|  | 7612697 |
|  | 7613043 |
|  | 7624126 |
|  | 7624143 |
|  | 7641208 |
|  | 7643225 |
|  | 7645964 |
|  | 7651732 |
|  | 7657167 |
|  | 7663512 |
|  | 7663513 |
|  | 7664260 |
|  | 7667322 |
|  | 7677753 |
|  | 7705663 |
|  | 7722455 |
|  | 7728766 |
|  | 7731676 |
|  | 7743504 |
|  | 7753548 |
|  | 7761096 |
|  | 7767001 |
|  | 7767977 |
|  | 7773278 |
|  | 7773294 |
|  | 7774811 |
|  | 7784075 |
|  | 7791795 |
|  | 7818760 |
|  | 7818898 |
|  | 7824276 |
|  | 7828835 |
|  | 7838530 |
|  | 7845681 |
|  | 7862450 |
|  | 7863329 |
|  | 7867001 |
|  | 7880533 |
|  | 7880883 |
|  | 7891874 |
|  | 7893366 |
|  | 7909518 |
|  | 7912076 |
|  | 7922305 |
|  | 7936657 |
|  | 7947386 |
|  | 7951317 |
|  | 7954410 |
|  | 7957050 |
|  | 7957051 |
|  | 7958853 |
|  | 7961103 |
|  | 7969181 |
|  | 7970733 |
|  | 7982204 |
|  | 7986270 |
|  | 7987394 |
|  | 7999265 |
|  | 8012956 |
|  | 8026887 |
|  | 8041741 |
|  | 8048955 |
|  | 8065163 |
|  | 8069906 |
|  | 8069917 |
|  | 8072529 |
|  | 8077211 |
|  | 8091232 |
|  | 8096197 |
|  | 8099936 |
|  | 8103211 |
|  | 8114705 |
|  | 8123467 |
|  | 8142011 |
|  | 8152809 |
|  | 8168036 |
|  | 8168855 |
|  | 8183575 |
|  | 8183578 |
|  | 8183579 |
|  | 8239076 |
|  | 8242752 |
|  | 8247544 |
|  | 8251184 |
|  | 8274450 |
|  | 8275085 |
|  | 8280378 |
|  | 8290271 |
|  | 8344491 |
|  | 8374952 |
|  | 8382357 |
|  | 8415631 |
|  | 8415656 |
|  | 8439732 |
|  | 8440237 |
|  | 8449498 |
|  | 8479522 |
|  | 8479523 |
|  | 8507984 |
|  | 8521821 |
|  | 8524262 |
|  | 8526886 |
|  | 8536852 |
|  | 8552621 |
|  | 8567126 |
|  | 8598286 |
|  | 8603071 |
|  | 8612582 |
|  | 8626390 |
|  | 8632996 |
|  | 8635454 |
|  | 8642041 |
|  | 8642629 |
|  | 8644854 |
|  | 8660987 |
|  | 8661696 |
|  | 8666771 |
|  | 8666950 |
|  | 8667605 |
|  | 8674054 |
|  | 8674108 |
|  | 8679004 |
|  | 8683100 |
|  | 8698618 |
|  | 8700548 |
|  | 8702776 |
|  | 8714088 |
|  | 8742032 |
|  | 8744767 |
|  | 8747929 |
|  | 8769647 |
|  | 8769648 |
|  | 8778285 |
|  | 8786661 |
|  | 8788039 |
|  | 8792932 |
|  | 8804306 |
|  | 8808709 |
|  | 8813135 |
|  | 8815878 |
|  | 8824316 |
|  | 8833235 |
|  | 8833912 |
|  | 8843191 |
|  | 8846919 |
|  | 8846920 |
|  | 8887548 |
|  | 8903730 |
|  | 8910340 |
|  | 8910602 |
|  | 8910901 |
|  | 8912842 |
|  | 8917731 |
|  | 8918879 |
|  | 8940078 |
|  | 8943020 |
|  | 8943068 |
|  | 8945939 |
|  | 8947040 |
|  | 8957077 |
|  | 8957082 |
|  | 8982467 |
|  | 8982609 |
|  | 8994835 |
|  | 9000131 |
|  | 9001225 |
|  | 9001226 |
|  | 9006086 |
|  | 9006089 |
|  | 9024662 |
|  | 9039259 |
|  | 9044841 |
|  | 9051119 |
|  | 9060424 |
|  | 9079825 |
|  | 9087426 |
|  | 9098922 |
|  | 9110404 |
|  | 9119951 |
|  | 9120389 |
|  | 9120720 |
|  | 9142118 |
|  | 9153395 |
|  | 9153396 |
|  | 9160668 |
|  | 9163685 |
|  | 9169087 |
|  | 9171368 |
|  | 9171369 |
|  | 9190891 |
|  | 9207798 |
|  | 9214616 |
|  | 9216621 |
|  | 9218592 |
|  | 9230186 |
|  | 9241268 |
|  | 9241281 |
|  | 9244349 |
|  | 9256421 |
|  | 9269892 |
|  | 9271585 |
|  | 9276376 |
|  | 9279659 |
|  | 9285554 |
|  | 9288110 |
|  | 9294016 |
|  | 9294187 |
|  | 9294611 |
|  | 9298894 |
|  | 9305847 |
|  | 9307024 |
|  | 9315097 |
|  | 9326930 |
|  | 9330618 |
|  | 9337410 |
|  | 9352120 |
|  | 9354418 |
|  | 9360984 |
|  | 9364206 |
|  | 9380404 |
|  | 9390024 |
|  | 9398849 |
|  | 9398851 |
|  | 9400702 |
|  | 9405685 |
|  | 9416841 |
|  | 9417035 |
|  | 9417861 |
|  | 9418856 |
|  | 9419352 |
|  | 9424091 |
|  | 9425892 |
|  | 9425920 |
|  | 9434955 |
|  | 9453487 |
|  | 9454845 |
|  | 9460995 |
|  | 9462635 |
|  | 9466932 |
|  | 9472983 |
|  | 9485015 |
|  | 9485045 |
|  | 9485488 |
|  | 9486848 |
|  | 9486849 |
|  | 9488455 |
|  | 9493073 |
|  | 9496889 |
|  | 9500548 |
|  | 9519874 |
|  | 9524576 |
|  | 9537435 |
|  | 9553042 |
|  | 9556576 |
|  | 9560157 |
|  | 9584189 |
|  | 9600120 |
|  | 9600977 |
|  | 9608530 |
|  | 9610736 |
|  | 9632822 |
|  | 9651526 |
|  | 9652741 |
|  | 9652742 |
|  | 9659582 |
|  | 9671769 |
|  | 9674698 |
|  | 9679979 |
|  | 9685670 |
|  | 9694807 |
|  | 9699732 |
|  | 9707425 |
|  | 9715507 |
|  | 9715509 |
|  | 9731488 |
|  | 9746439 |
|  | 9755213 |
|  | 9761753 |
|  | 9771644 |
|  | 9771937 |
|  | 9772279 |
|  | 9792801 |
|  | 9794235 |
|  | 9794240 |
|  | 9824692 |
|  | 9839726 |
|  | 9843989 |
|  | 9851850 |
|  | 9851974 |
|  | 9861017 |
|  | 9872327 |
|  | 9875313 |
|  | 9892179 |
|  | 9917000 |
|  | 9950212 |
|  | 9988767 |
|  | 10065153 |
|  | 10078201 |
|  | 10198638 |
|  | 10214938 |
|  | 10347180 |
|  | 10348343 |
|  | 10358050 |
|  | 10380882 |
|  | 10415337 |
|  | 10468612 |
|  | 10470151 |
|  | 10477429 |
|  | 10484981 |
|  | 10597287 |
|  | 10606744 |
|  | 10608892 |
|  | 10629029 |
|  | 10634809 |
|  | 10644693 |
|  | 10644996 |
|  | 10666337 |
|  | 10673501 |
|  | 10713716 |
|  | 10721693 |
|  | 10736619 |
|  | 10744705 |
|  | 10747897 |
|  | 10749849 |
|  | 10766163 |
|  | 10794522 |
|  | 10823891 |
|  | 10825186 |
|  | 10837489 |
|  | 10842192 |
|  | 10848610 |
|  | 10853038 |
|  | 10876243 |
|  | 10882116 |
|  | 1088347 |
|  | 10884347 |
|  | 10891493 |
|  | 10913176 |
|  | 10913622 |
|  | 10930427 |
|  | 10942770 |
|  | 10951572 |
|  | 10958792 |
|  | 10961991 |
|  | 11002423 |
|  | 11025664 |
|  | 11046142 |
|  | 11050162 |
|  | 11058590 |
|  | 11080152 |
|  | 11080164 |
|  | 11094089 |
|  | 11099047 |
|  | 11106654 |
|  | 11118038 |
|  | 11146555 |
|  | 11172000 |
|  | 11175333 |
|  | 11196199 |
|  | 11238924 |
|  | 11245491 |
|  | 11251186 |
|  | 11258898 |
|  | 11278372 |
|  | 11278647 |
|  | 11283254 |
|  | 11285227 |
|  | 11297531 |
|  | 11311202 |
|  | 11327730 |
|  | 11328884 |
|  | 11340159 |
|  | 11359905 |
|  | 11360197 |
|  | 11403041 |
|  | 11409876 |
|  | 11416144 |
|  | 11420669 |
|  | 11427532 |
|  | 11430832 |
|  | 11447225 |
|  | 11481490 |
|  | 11483158 |
|  | 11486030 |
|  | 11500059 |
|  | 11507088 |
|  | 11511362 |
|  | 11518545 |
|  | 11527429 |
|  | 11546806 |
|  | 11551930 |
|  | 11554765 |
|  | 11559355 |
|  | 11562347 |
|  | 11583632 |
|  | 11600572 |
|  | 11602059 |
|  | 11672523 |
|  | 11684014 |
|  | 11687965 |
|  | 11692157 |
|  | 11704667 |
|  | 11704853 |
|  | 11706030 |
|  | 11707401 |
|  | 11707411 |
|  | 11708408 |
|  | 11709713 |
|  | 11718557 |
|  | 11733360 |
|  | 11737306 |
|  | 11739724 |
|  | 11744990 |
|  | 11748221 |
|  | 11748232 |
|  | 11751390 |
|  | 11751391 |
|  | 11751402 |
|  | 11751427 |
|  | 11751853 |
|  | 11753428 |
|  | 11755471 |
|  | 11756653 |
|  | 11763417 |
|  | 11764099 |
|  | 11776898 |
|  | 11779500 |
|  | 11779855 |
|  | 11780126 |
|  | 11781842 |
|  | 11782467 |
|  | 11783017 |
|  | 11783119 |
|  | 11786482 |
|  | 11788906 |
|  | 11792711 |
|  | 11793443 |
|  | 11793474 |
|  | 11804609 |
|  | 11804688 |
|  | 11805092 |
|  | 11805286 |
|  | 11807792 |
|  | 11809417 |
|  | 11818510 |
|  | 11818669 |
|  | 11821948 |
|  | 11838964 |
|  | 11838966 |
|  | 11839095 |
|  | 11840332 |
|  | 11842245 |
|  | 11844595 |
|  | 11844788 |
|  | 11844800 |
|  | 11844810 |
|  | 11852106 |
|  | 11854266 |
|  | 11857304 |
|  | 11859067 |
|  | 11859195 |
|  | 11859970 |
|  | 11861393 |
|  | 11861400 |
|  | 11861836 |
|  | 11865061 |
|  | 11867628 |
|  | 11867732 |
|  | 11872638 |
|  | 11875057 |
|  | 11875500 |
|  | 11877395 |
|  | 11880381 |
|  | 11881786 |
|  | 11883440 |
|  | 11883897 |
|  | 11887018 |
|  | 11890931 |
|  | 11892796 |
|  | 11893247 |
|  | 11893750 |
|  | 11895857 |
|  | 11896572 |
|  | 11900859 |
|  | 11911244 |
|  | 11915341 |
|  | 11917009 |
|  | 11919562 |
|  | 11920528 |
|  | 11923280 |
|  | 11923872 |
|  | 11925430 |
|  | 11925449 |
|  | 11925591 |
|  | 11934017 |
|  | 11935300 |
|  | 11936585 |
|  | 11940482 |
|  | 11948395 |
|  | 11948396 |
|  | 11948632 |
|  | 11949842 |
|  | 11949843 |
|  | 11950834 |
|  | 11953436 |
|  | 11953857 |
|  | 11953859 |
|  | 11956602 |
|  | 11956638 |
|  | 11957139 |
|  | 11960007 |
|  | 11960374 |
|  | 11960383 |
|  | 11960904 |
|  | 11960918 |
|  | 11961303 |
|  | 11964141 |
|  | 11978184 |
|  | 11979459 |
|  | 11980658 |
|  | 11988847 |
|  | 11992381 |
|  | 11992547 |
|  | 11992551 |
|  | 11992552 |
|  | 11997498 |
|  | 11999565 |
|  | 12007715 |
|  | 12011055 |
|  | 12011072 |
|  | 12011430 |
|  | 12014634 |
|  | 12032659 |
|  | 12032848 |
|  | 12034444 |
|  | 12034820 |
|  | 12034830 |
|  | 12036888 |
|  | 12036943 |
|  | 12039466 |
|  | 12045262 |
|  | 12046064 |
|  | 12046074 |
|  | 12048243 |
|  | 12048682 |
|  | 12049739 |
|  | 12051912 |
|  | 12060398 |
|  | 12062821 |
|  | 12064478 |
|  | 12065086 |
|  | 12071847 |
|  | 12073053 |
|  | 12077306 |
|  | 12077343 |
|  | 12080066 |
|  | 12080348 |
|  | 12082526 |
|  | 12082529 |
|  | 12082608 |
|  | 12082615 |
|  | 12082636 |
|  | 12084347 |
|  | 12085209 |
|  | 12089322 |
|  | 12091386 |
|  | 12093899 |
|  | 12096338 |
|  | 12099684 |
|  | 12101184 |
|  | 12105992 |
|  | 12107116 |
|  | 12110584 |
|  | 12110597 |
|  | 12112314 |
|  | 12119414 |
|  | 12124396 |
|  | 12127401 |
|  | 12127695 |
|  | 12136132 |
|  | 12136662 |
|  | 12138177 |
|  | 12138209 |
|  | 12145207 |
|  | 12145320 |
|  | 12151395 |
|  | 12154087 |
|  | 12160929 |
|  | 12161427 |
|  | 12164929 |
|  | 12165799 |
|  | 12167711 |
|  | 12167722 |
|  | 12168898 |
|  | 12168936 |
|  | 12169206 |
|  | 12171773 |
|  | 12172977 |
|  | 12174591 |
|  | 12174820 |
|  | 12174901 |
|  | 12174951 |
|  | 12175902 |
|  | 12186903 |
|  | 12190289 |
|  | 12194286 |
|  | 12198151 |
|  | 12208736 |
|  | 12209584 |
|  | 12209590 |
|  | 12209989 |
|  | 12217802 |
|  | 12221910 |
|  | 12226108 |
|  | 12234998 |
|  | 12235242 |
|  | 12242109 |
|  | 12242661 |
|  | 12270344 |
|  | 12324477 |
|  | 12351827 |
|  | 12355087 |
|  | 12367518 |
|  | 12370303 |
|  | 12370812 |
|  | 12372430 |
|  | 12372616 |
|  | 12374798 |
|  | 12379854 |
|  | 12380307 |
|  | 12381304 |
|  | 12383858 |
|  | 12384512 |
|  | 12384701 |
|  | 12386810 |
|  | 12386819 |
|  | 12388558 |
|  | 12392301 |
|  | 12393814 |
|  | 12393902 |
|  | 12403781 |
|  | 12411481 |
|  | 12419336 |
|  | 12419825 |
|  | 12420214 |
|  | 12420220 |
|  | 12421820 |
|  | 12426395 |
|  | 12427745 |
|  | 12427754 |
|  | 12429911 |
|  | 12432277 |
|  | 12433927 |
|  | 12434001 |
|  | 12434121 |
|  | 12438652 |
|  | 12439747 |
|  | 12444079 |
|  | 12445188 |
|  | 12446718 |
|  | 12446780 |
|  | 12447653 |
|  | 12451999 |
|  | 12452037 |
|  | 12452062 |
|  | 12459171 |
|  | 12459315 |
|  | 12459451 |
|  | 12459463 |
|  | 12459877 |
|  | 12468914 |
|  | 12471108 |
|  | 12474051 |
|  | 12479364 |
|  | 12479855 |
|  | 12480939 |
|  | 12482984 |
|  | 12492368 |
|  | 12492369 |
|  | 12492370 |
|  | 12496062 |
|  | 12496474 |
|  | 12496479 |
|  | 12496480 |
|  | 12499093 |
|  | 12501250 |
|  | 12503212 |
|  | 12505256 |
|  | 12507556 |
|  | 12508351 |
|  | 12514180 |
|  | 12515622 |
|  | 12518062 |
|  | 12519780 |
|  | 12524418 |
|  | 12524540 |
|  | 12526791 |
|  | 12527892 |
|  | 12527938 |
|  | 12529318 |
|  | 12532420 |
|  | 12534345 |
|  | 12535653 |
|  | 12538356 |
|  | 12543796 |
|  | 12548019 |
|  | 12550754 |
|  | 12552135 |
|  | 12553064 |
|  | 12556232 |
|  | 12556559 |
|  | 12562237 |
|  | 12563316 |
|  | 12565178 |
|  | 12567188 |
|  | 12568865 |
|  | 12569576 |
|  | 12573582 |
|  | 12574499 |
|  | 12575534 |
|  | 12584563 |
|  | 12591737 |
|  | 12592393 |
|  | 12600206 |
|  | 12606585 |
|  | 12607595 |
|  | 12607601 |
|  | 12607604 |
|  | 12609716 |
|  | 12610779 |
|  | 12612087 |
|  | 12615706 |
|  | 12615712 |
|  | 12620407 |
|  | 12620801 |
|  | 12628922 |
|  | 12628923 |
|  | 12634062 |
|  | 12637159 |
|  | 12640129 |
|  | 12642583 |
|  | 12642870 |
|  | 12642871 |
|  | 12646262 |
|  | 12654245 |
|  | 12660163 |
|  | 12665570 |
|  | 12668287 |
|  | 12669312 |
|  | 12670866 |
|  | 12670900 |
|  | 12673251 |
|  | 12679912 |
|  | 12687275 |
|  | 12687276 |
|  | 12690203 |
|  | 12692266 |
|  | 12694871 |
|  | 12698195 |
|  | 12698197 |
|  | 12700230 |
|  | 12702766 |
|  | 12704785 |
|  | 12706118 |
|  | 12712134 |
|  | 12713809 |
|  | 12716906 |
|  | 12724314 |
|  | 12725329 |
|  | 12725330 |
|  | 12725531 |
|  | 12725534 |
|  | 12727844 |
|  | 12731623 |
|  | 12732139 |
|  | 12735900 |
|  | 12738987 |
|  | 12743423 |
|  | 12743601 |
|  | 12748190 |
|  | 12749011 |
|  | 12750254 |
|  | 12750285 |
|  | 12750368 |
|  | 12756585 |
|  | 12759240 |
|  | 12759537 |
|  | 12761448 |
|  | 12766905 |
|  | 12767266 |
|  | 12768030 |
|  | 12771886 |
|  | 12772781 |
|  | 12773566 |
|  | 12779080 |
|  | 12782403 |
|  | 12782576 |
|  | 12782597 |
|  | 12788915 |
|  | 12789271 |
|  | 12789273 |
|  | 12794118 |
|  | 12794243 |
|  | 12800224 |
|  | 12804595 |
|  | 12808109 |
|  | 12809883 |
|  | 12811820 |
|  | 12818446 |
|  | 12820330 |
|  | 12820372 |
|  | 12821135 |
|  | 12821780 |
|  | 12821781 |
|  | 12821945 |
|  | 12823440 |
|  | 12823592 |
|  | 12824702 |
|  | 12824925 |
|  | 12826609 |
|  | 12829999 |
|  | 12839924 |
|  | 12839966 |
|  | 12841598 |
|  | 12841870 |
|  | 12851404 |
|  | 12854888 |
|  | 12857899 |
|  | 12860918 |
|  | 12871978 |
|  | 12874009 |
|  | 12875991 |
|  | 12879009 |
|  | 12885235 |
|  | 12888634 |
|  | 12890671 |
|  | 12893432 |
|  | 12894226 |
|  | 12898523 |
|  | 12901798 |
|  | 12901974 |
|  | 12905527 |
|  | 12907596 |
|  | 12907646 |
|  | 12911720 |
|  | 12915590 |
|  | 12920132 |
|  | 12923319 |
|  | 12926050 |
|  | 12926080 |
|  | 12926093 |
|  | 12926120 |
|  | 12926130 |
|  | 12926152 |
|  | 12931731 |
|  | 12939400 |
|  | 12942774 |
|  | 12947108 |
|  | 12954772 |
|  | 12955365 |
|  | 12958068 |
|  | 12959929 |
|  | 12963547 |
|  | 12969350 |
|  | 12970887 |
|  | 12972634 |
|  | 1349102 |
|  | 1349175 |
|  | 13678722 |
|  | 13679039 |
|  | 13679996 |
|  | 14499615 |
|  | 14500353 |
|  | 14500711 |
|  | 14500729 |
|  | 14504475 |
|  | 14511408 |
|  | 14517211 |
|  | 14517280 |
|  | 14519637 |
|  | 14519663 |
|  | 14523002 |
|  | 14527689 |
|  | 14529662 |
|  | 14532990 |
|  | 14534297 |
|  | 14534696 |
|  | 14534749 |
|  | 1454855 |
|  | 14551300 |
|  | 14557644 |
|  | 14557665 |
|  | 14559824 |
|  | 14560002 |
|  | 14566828 |
|  | 14572637 |
|  | 14576150 |
|  | 14576823 |
|  | 14582705 |
|  | 14583449 |
|  | 14583450 |
|  | 14583461 |
|  | 14584049 |
|  | 14586398 |
|  | 14586402 |
|  | 14592451 |
|  | 14595650 |
|  | 14607337 |
|  | 14608092 |
|  | 14612290 |
|  | 14612402 |
|  | 14612423 |
|  | 14612427 |
|  | 14612499 |
|  | 14612511 |
|  | 14612513 |
|  | 14618087 |
|  | 14623330 |
|  | 14623878 |
|  | 14627345 |
|  | 14628083 |
|  | 14637168 |
|  | 14640983 |
|  | 14644318 |
|  | 14645509 |
|  | 14645574 |
|  | 14647426 |
|  | 14647431 |
|  | 14648233 |
|  | 1465435 |
|  | 14654539 |
|  | 14654549 |
|  | 14654903 |
|  | 14654927 |
|  | 14656244 |
|  | 14660794 |
|  | 14663467 |
|  | 14669308 |
|  | 14670539 |
|  | 14671306 |
|  | 14675203 |
|  | 14675665 |
|  | 14676802 |
|  | 14676844 |
|  | 14678961 |
|  | 14680293 |
|  | 14680497 |
|  | 14682374 |
|  | 14689061 |
|  | 14690015 |
|  | 14693272 |
|  | 14699137 |
|  | 14704000 |
|  | 14707141 |
|  | 14710355 |
|  | 14714296 |
|  | 14716820 |
|  | 14719105 |
|  | 14719118 |
|  | 14722922 |
|  | 14724564 |
|  | 14729628 |
|  | 14730312 |
|  | 14735465 |
|  | 14741214 |
|  | 14743210 |
|  | 14744758 |
|  | 14744935 |
|  | 14745598 |
|  | 14749371 |
|  | 14755251 |
|  | 14756543 |
|  | 14756544 |
|  | 14757278 |
|  | 14757832 |
|  | 14759115 |
|  | 14761400 |
|  | 14960324 |
|  | 14960327 |
|  | 14961077 |
|  | 14964781 |
|  | 14965474 |
|  | 14966292 |
|  | 14967026 |
|  | 14968111 |
|  | 14981901 |
|  | 14982997 |
|  | 14986171 |
|  | 14990579 |
|  | 14991543 |
|  | 14992692 |
|  | 14994997 |
|  | 14995078 |
|  | 15004035 |
|  | 15015569 |
|  | 15016620 |
|  | 15016801 |
|  | 15018706 |
|  | 15026322 |
|  | 15033824 |
|  | 15033825 |
|  | 15033827 |
|  | 15037740 |
|  | 15039530 |
|  | 15041737 |
|  | 15042092 |
|  | 15047823 |
|  | 15052934 |
|  | 15052938 |
|  | 15058298 |
|  | 15059885 |
|  | 15060172 |
|  | 15061204 |
|  | 15064706 |
|  | 15064727 |
|  | 15064730 |
|  | 15064747 |
|  | 15067011 |
|  | 15067062 |
|  | 15069555 |
|  | 15069711 |
|  | 15073118 |
|  | 15077116 |
|  | 15077166 |
|  | 15077194 |
|  | 15081425 |
|  | 15082766 |
|  | 15087457 |
|  | 15088070 |
|  | 15094062 |
|  | 15095978 |
|  | 15099937 |
|  | 15102863 |
|  | 15103385 |
|  | 15104366 |
|  | 15111320 |
|  | 15116093 |
|  | 15116098 |
|  | 15116721 |
|  | 15120697 |
|  | 15121773 |
|  | 15123817 |
|  | 15131018 |
|  | 15131588 |
|  | 15133049 |
|  | 15136035 |
|  | 15145527 |
|  | 15149157 |
|  | 15149158 |
|  | 15149862 |
|  | 15153330 |
|  | 15159397 |
|  | 15161716 |
|  | 15168247 |
|  | 15170665 |
|  | 15178678 |
|  | 15186775 |
|  | 15187095 |
|  | 15192123 |
|  | 15195682 |
|  | 15199132 |
|  | 15199139 |
|  | 15199150 |
|  | 15201954 |
|  | 15201971 |
|  | 15201979 |
|  | 15202001 |
|  | 15208672 |
|  | 15216398 |
|  | 15218947 |
|  | 15220443 |
|  | 15222050 |
|  | 15225611 |
|  | 15226443 |
|  | 15231748 |
|  | 15234210 |
|  | 15247280 |
|  | 15254760 |
|  | 15256449 |
|  | 15262986 |
|  | 15273725 |
|  | 15273730 |
|  | 15279793 |
|  | 15280377 |
|  | 15282313 |
|  | 15284248 |
|  | 15284293 |
|  | 15284338 |
|  | 15291355 |
|  | 15292938 |
|  | 15295102 |
|  | 15297421 |
|  | 15297424 |
|  | 15299072 |
|  | 15304330 |
|  | 15308643 |
|  | 15310147 |
|  | 15310756 |
|  | 15310764 |
|  | 15314173 |
|  | 15314174 |
|  | 15314287 |
|  | 15320716 |
|  | 15321712 |
|  | 15324660 |
|  | 15327968 |
|  | 15331571 |
|  | 15331608 |
|  | 15334053 |
|  | 15334064 |
|  | 15334668 |
|  | 15337531 |
|  | 15339933 |
|  | 15340061 |
|  | 15341441 |
|  | 15342238 |
|  | 15342377 |
|  | 15350302 |
|  | 15351976 |
|  | 15358177 |
|  | 15358195 |
|  | 15358233 |
|  | 15358769 |
|  | 15358771 |
|  | 15359011 |
|  | 15361841 |
|  | 15367600 |
|  | 15367885 |
|  | 15371422 |
|  | 15371445 |
|  | 15373786 |
|  | 15377670 |
|  | 15378004 |
|  | 15378026 |
|  | 15381368 |
|  | 15383276 |
|  | 15386362 |
|  | 15386430 |
|  | 15388813 |
|  | 15390294 |
|  | 15448009 |
|  | 15448010 |
|  | 15450681 |
|  | 15456759 |
|  | 15456784 |
|  | 15466207 |
|  | 15467428 |
|  | 15469934 |
|  | 15469940 |
|  | 15471885 |
|  | 15472230 |
|  | 15474092 |
|  | 15485928 |
|  | 15489903 |
|  | 15492790 |
|  | 15497017 |
|  | 15498930 |
|  | 15500550 |
|  | 15509555 |
|  | 15509808 |
|  | 15510160 |
|  | 15525938 |
|  | 15531928 |
|  | 15532030 |
|  | 15534934 |
|  | 15537749 |
|  | 15539839 |
|  | 15539956 |
|  | 15542844 |
|  | 15545228 |
|  | 15545268 |
|  | 15548678 |
|  | 15548685 |
|  | 15548700 |
|  | 15550400 |
|  | 15554555 |
|  | 15557124 |
|  | 15557758 |
|  | 15563462 |
|  | 15569630 |
|  | 15574328 |
|  | 15574785 |
|  | 15577914 |
|  | 15578720 |
|  | 15580300 |
|  | 15580302 |
|  | 15582998 |
|  | 15583825 |
|  | 15601585 |
|  | 15606307 |
|  | 15608028 |
|  | 15608668 |
|  | 15609309 |
|  | 15611070 |
|  | 15611139 |
|  | 15613472 |
|  | 15629713 |
|  | 15632057 |
|  | 15633234 |
|  | 15634909 |
|  | 15638389 |
|  | 15642401 |
|  | 15644269 |
|  | 15645452 |
|  | 15650224 |
|  | 1565143 |
|  | 1565144 |
|  | 15657147 |
|  | 15657348 |
|  | 15657356 |
|  | 15659406 |
|  | 15659650 |
|  | 15660129 |
|  | 15661746 |
|  | 15664194 |
|  | 15670751 |
|  | 15671037 |
|  | 15671551 |
|  | 15674341 |
|  | 15674351 |
|  | 15680394 |
|  | 15683227 |
|  | 15688418 |
|  | 15696860 |
|  | 15701641 |
|  | 15701968 |
|  | 15701975 |
|  | 15702478 |
|  | 15703170 |
|  | 15705792 |
|  | 15705871 |
|  | 15707991 |
|  | 15709766 |
|  | 15710230 |
|  | 15710329 |
|  | 15713654 |
|  | 15713979 |
|  | 15719270 |
|  | 15720412 |
|  | 15721419 |
|  | 15723837 |
|  | 15731778 |
|  | 15734290 |
|  | 15734740 |
|  | 15735102 |
|  | 15741235 |
|  | 15750621 |
|  | 15752352 |
|  | 15752772 |
|  | 15753095 |
|  | 15764300 |
|  | 15764647 |
|  | 15765147 |
|  | 15770529 |
|  | 15772596 |
|  | 15777220 |
|  | 15777783 |
|  | 15778501 |
|  | 15781620 |
|  | 15782125 |
|  | 15782130 |
|  | 15790310 |
|  | 15791651 |
|  | 15793143 |
|  | 15795472 |
|  | 15796908 |
|  | 15798209 |
|  | 15800978 |
|  | 15802048 |
|  | 15806145 |
|  | 15808506 |
|  | 15811121 |
|  | 15814626 |
|  | 15816455 |
|  | 15816541 |
|  | 15819981 |
|  | 15824059 |
|  | 15824734 |
|  | 15824742 |
|  | 15830705 |
|  | 15832422 |
|  | 15832769 |
|  | 15833845 |
|  | 15838728 |
|  | 15841715 |
|  | 15843377 |
|  | 15844633 |
|  | 15846069 |
|  | 15846088 |
|  | 15848166 |
|  | 15848594 |
|  | 15849742 |
|  | 15850555 |
|  | 15851479 |
|  | 15851483 |
|  | 15855171 |
|  | 15856024 |
|  | 15865924 |
|  | 15865925 |
|  | 15865935 |
|  | 15866118 |
|  | 15876864 |
|  | 15878620 |
|  | 15882069 |
|  | 15882981 |
|  | 15888975 |
|  | 15900046 |
|  | 15902285 |
|  | 15902745 |
|  | 15905198 |
|  | 15905205 |
|  | 15906354 |
|  | 15906362 |
|  | 15907477 |
|  | 15908423 |
|  | 15911628 |
|  | 15913547 |
|  | 1591732 |
|  | 15917726 |
|  | 15925988 |
|  | 15927816 |
|  | 15937335 |
|  | 15941720 |
|  | 15942083 |
|  | 15942101 |
|  | 15943041 |
|  | 15943410 |
|  | 15945132 |
|  | 15950944 |
|  | 15958617 |
|  | 15961390 |
|  | 15964625 |
|  | 15964795 |
|  | 15967108 |
|  | 15981097 |
|  | 15993272 |
|  | 15993843 |
|  | 15998523 |
|  | 15998635 |
|  | 16007153 |
|  | 16007163 |
|  | 16009172 |
|  | 1600943 |
|  | 16012716 |
|  | 16033918 |
|  | 16037820 |
|  | 16038035 |
|  | 16039051 |
|  | 16043423 |
|  | 16046401 |
|  | 16051302 |
|  | 16054204 |
|  | 16055707 |
|  | 16061860 |
|  | 16077963 |
|  | 16082197 |
|  | 16082221 |
|  | 16082224 |
|  | 16082226 |
|  | 16088313 |
|  | 16091363 |
|  | 16094360 |
|  | 16094622 |
|  | 16101161 |
|  | 16103882 |
|  | 16107721 |
|  | 16107876 |
|  | 16109171 |
|  | 16112647 |
|  | 16122737 |
|  | 16125276 |
|  | 16125822 |
|  | 16129685 |
|  | 16131611 |
|  | 16140998 |
|  | 16142332 |
|  | 16142358 |
|  | 16142387 |
|  | 16151725 |
|  | 16158057 |
|  | 16159876 |
|  | 16159878 |
|  | 16163384 |
|  | 16168122 |
|  | 16169070 |
|  | 16169726 |
|  | 16170349 |
|  | 16174251 |
|  | 16177659 |
|  | 16189514 |
|  | 16199549 |
|  | 16202543 |
|  | 16203738 |
|  | 16203772 |
|  | 16203773 |
|  | 16212876 |
|  | 16226255 |
|  | 16227626 |
|  | 16228292 |
|  | 16229746 |
|  | 16230356 |
|  | 16243804 |
|  | 16243918 |
|  | 16246554 |
|  | 16246896 |
|  | 16247442 |
|  | 16249378 |
|  | 16251187 |
|  | 16255765 |
|  | 16256294 |
|  | 16258285 |
|  | 16260623 |
|  | 16264229 |
|  | 16264230 |
|  | 16268414 |
|  | 16270158 |
|  | 16272802 |
|  | 16273189 |
|  | 16273227 |
|  | 16273238 |
|  | 16273653 |
|  | 16283431 |
|  | 16286925 |
|  | 16287156 |
|  | 16289694 |
|  | 16291740 |
|  | 16293709 |
|  | 16297369 |
|  | 16299809 |
|  | 16301995 |
|  | 1631137 |
|  | 1631151 |
|  | 16313886 |
|  | 16314517 |
|  | 16318864 |
|  | 16322335 |
|  | 16325212 |
|  | 16327987 |
|  | 16328005 |
|  | 16343421 |
|  | 16343438 |
|  | 16344056 |
|  | 16344560 |
|  | 16351648 |
|  | 16352595 |
|  | 16354195 |
|  | 16354677 |
|  | 16358373 |
|  | 16360120 |
|  | 16364249 |
|  | 16376985 |
|  | 16377624 |
|  | 16380082 |
|  | 16380088 |
|  | 16412606 |
|  | 16416221 |
|  | 16418264 |
|  | 16418486 |
|  | 16419081 |
|  | 16430884 |
|  | 16432196 |
|  | 16432214 |
|  | 16432227 |
|  | 16436515 |
|  | 16438982 |
|  | 16440000 |
|  | 16442532 |
|  | 16443602 |
|  | 16446369 |
|  | 16446403 |
|  | 16447252 |
|  | 16448675 |
|  | 1644930 |
|  | 16449651 |
|  | 16455050 |
|  | 16455055 |
|  | 16455125 |
|  | 16458330 |
|  | 16458891 |
|  | 16461914 |
|  | 16461916 |
|  | 16462759 |
|  | 16462765 |
|  | 16469747 |
|  | 16474133 |
|  | 16474402 |
|  | 16477330 |
|  | 16479062 |
|  | 16481301 |
|  | 16482544 |
|  | 16485142 |
|  | 16487937 |
|  | 16489069 |
|  | 16489641 |
|  | 16492679 |
|  | 16499995 |
|  | 16504179 |
|  | 16506220 |
|  | 16507123 |
|  | 16507995 |
|  | 16516153 |
|  | 16518869 |
|  | 16521213 |
|  | 16525651 |
|  | 16525665 |
|  | 16525684 |
|  | 16529749 |
|  | 16530164 |
|  | 16531411 |
|  | 16533178 |
|  | 16541312 |
|  | 16546135 |
|  | 16547496 |
|  | 16547498 |
|  | 16548519 |
|  | 16551852 |
|  | 16552184 |
|  | 16552814 |
|  | 16569330 |
|  | 16569765 |
|  | 16579792 |
|  | 16581806 |
|  | 16582625 |
|  | 16595680 |
|  | 16600182 |
|  | 16601678 |
|  | 16601838 |
|  | 16609366 |
|  | 16611260 |
|  | 16611888 |
|  | 16616141 |
|  | 16616891 |
|  | 16619038 |
|  | 16619042 |
|  | 16620772 |
|  | 16620878 |
|  | 16621805 |
|  | 16624812 |
|  | 16625286 |
|  | 16636310 |
|  | 16636674 |
|  | 16646561 |
|  | 16652373 |
|  | 16677609 |
|  | 16678111 |
|  | 16681440 |
|  | 16682816 |
|  | 16682957 |
|  | 16684540 |
|  | 16686942 |
|  | 16687402 |
|  | 16690937 |
|  | 16698048 |
|  | 16704422 |
|  | 16705155 |
|  | 16705183 |
|  | 16708390 |
|  | 16713569 |
|  | 16714289 |
|  | 16717128 |
|  | 16721787 |
|  | 16723121 |
|  | 1672732 |
|  | 16728565 |
|  | 16729031 |
|  | 1673792 |
|  | 16737921 |
|  | 16740634 |
|  | 16741250 |
|  | 16741917 |
|  | 16741955 |
|  | 16741990 |
|  | 16751805 |
|  | 16753148 |
|  | 16754663 |
|  | 16773083 |
|  | 16773719 |
|  | 16777982 |
|  | 16777989 |
|  | 16777994 |
|  | 16778101 |
|  | 16778203 |
|  | 16778209 |
|  | 16782868 |
|  | 16784539 |
|  | 16785993 |
|  | 16786124 |
|  | 16786570 |
|  | 1679237 |
|  | 16797751 |
|  | 16798743 |
|  | 16799873 |
|  | 16803902 |
|  | 16810312 |
|  | 16810754 |
|  | 16814250 |
|  | 16815295 |
|  | 16820091 |
|  | 16821145 |
|  | 16825434 |
|  | 16827139 |
|  | 16827804 |
|  | 16830793 |
|  | 1683921 |
|  | 16839413 |
|  | 16844765 |
|  | 16847267 |
|  | 16847809 |
|  | 16847810 |
|  | 16855398 |
|  | 16861890 |
|  | 16865671 |
|  | 16867217 |
|  | 16869745 |
|  | 16870621 |
|  | 16872365 |
|  | 16875676 |
|  | 16880779 |
|  | 16893887 |
|  | 16894028 |
|  | 16905769 |
|  | 16909101 |
|  | 16912290 |
|  | 16912307 |
|  | 16916644 |
|  | 16916716 |
|  | 16917063 |
|  | 16918502 |
|  | 16918534 |
|  | 16919027 |
|  | 16920112 |
|  | 16930536 |
|  | 16937443 |
|  | 16941491 |
|  | 16943578 |
|  | 16948516 |
|  | 16951253 |
|  | 16951290 |
|  | 16959223 |
|  | 16959611 |
|  | 16964264 |
|  | 16969106 |
|  | 16969499 |
|  | 16972754 |
|  | 16980608 |
|  | 16982033 |
|  | 16983332 |
|  | 16984108 |
|  | 16984978 |
|  | 16990795 |
|  | 17000754 |
|  | 17001034 |
|  | 17009393 |
|  | 17010455 |
|  | 17013801 |
|  | 17014845 |
|  | 17014852 |
|  | 17028100 |
|  | 17030796 |
|  | 17043663 |
|  | 17050670 |
|  | 17050675 |
|  | 17050687 |
|  | 17054774 |
|  | 17054906 |
|  | 17055453 |
|  | 17056014 |
|  | 17060450 |
|  | 17060456 |
|  | 17062677 |
|  | 17065008 |
|  | 17069461 |
|  | 17070499 |
|  | 17072987 |
|  | 17077087 |
|  | 17077165 |
|  | 17082593 |
|  | 17085447 |
|  | 17091766 |
|  | 17093398 |
|  | 17101792 |
|  | 17108107 |
|  | 17110336 |
|  | 17110929 |
|  | 17132229 |
|  | 17136003 |
|  | 17139261 |
|  | 17143016 |
|  | 17149973 |
|  | 17150106 |
|  | 17161371 |
|  | 17172844 |
|  | 17172861 |
|  | 17178878 |
|  | 17188136 |
|  | 17189186 |
|  | 17189187 |
|  | 17198188 |
|  | 17213797 |
|  | 17214516 |
|  | 17214517 |
|  | 17214880 |
|  | 17227108 |
|  | 1762941 |
|  | 1849234 |
|  | 1915267 |
|  | 1933902 |
|  | 1939648 |
|  | 1978757 |
|  | 2047879 |
|  | 2259385 |
|  | 2531845 |
|  | 2905688 |
|  | 2946935 |
|  | 3001719 |
|  | 3025664 |
|  | 3894933 |
|  | 4006916 |
|  | 6396087 |
|  | 7587074 |
|  | 7663514 |
|  | 7686617 |
|  | 7720704 |
|  | 7753047 |
|  | 7761466 |
|  | 7777531 |
|  | 7809597 |
|  | 7811761 |
|  | 7818536 |
|  | 7935417 |
|  | 7935455 |
|  | 8016121 |
|  | 8023157 |
|  | 8028670 |
|  | 8058315 |
|  | 8118819 |
|  | 8134127 |
|  | 8183576 |
|  | 8207805 |
|  | 8316628 |
|  | 8364550 |
|  | 8389468 |
|  | 8401536 |
|  | 8423216 |
|  | 8513440 |
|  | 8617246 |
|  | 8623910 |
|  | 8649785 |
|  | 8668206 |
|  | 8675009 |
|  | 8816502 |
|  | 8825920 |
|  | 8875926 |
|  | 8875929 |
|  | 8895579 |
|  | 8921390 |
|  | 8943363 |
|  | 8961277 |
|  | 8986812 |
|  | 9010216 |
|  | 9119221 |
|  | 9129988 |
|  | 9143503 |
|  | 9183006 |
|  | 9194565 |
|  | 9226370 |
|  | 9288740 |
|  | 9312058 |
|  | 9349507 |
|  | 9369221 |
|  | 9372954 |
|  | 9373149 |
|  | 9380510 |
|  | 9393873 |
|  | 9440695 |
|  | 9444950 |
|  | 9472015 |
|  | 9482880 |
|  | 9492043 |
|  | 9537326 |
|  | 9565608 |
|  | 9569035 |
|  | 9571186 |
|  | 9620776 |
|  | 9628871 |
|  | 9685344 |
|  | 9732264 |
|  | 9744860 |
|  | 9765199 |
|  | 9809062 |
|  | 9811893 |
|  | 9840937 |
|  | 9843217 |
|  | 9891054 |
|  | 9891077 |
|  | 9926942 |
|  | 9935181 |
|  | 14588144 |
|  | 15064348 |
|  | 7626789 |
|  | 8535090 |
|  | 8807776 |
|  | 12477391 |
|  | 15630097 |
|  | 9200835 |
|  | 12015968 |
|  | 12443880 |
|  | 12859970 |
|  | 14659876 |
|  | 14667412 |
|  | 15529176 |
|  | 15590746 |
|  | 15827121 |
|  | 15914672 |
|  | 15984772 |
|  | 16096653 |
|  | 16109975 |
|  | 16273521 |
|  | 16322560 |
|  | 16364449 |
|  | 16408288 |
|  | 16464238 |
|  | 16601753 |
|  | 16616005 |
|  | 16799861 |
|  | 16888646 |
|  | 16904934 |
|  | 17081288 |
|  | 17510286 |
|  | 17530925 |
|  | 10656795 |
|  | 10850547 |
|  | 11007800 |
|  | 11023613 |
|  | 11239457 |
|  | 11554448 |
|  | 11740489 |
|  | 12413700 |
|  | 12507430 |
|  | 1303181 |
|  | 1327751 |
|  | 1394225 |
|  | 1437144 |
|  | 1459726 |
|  | 15053879 |
|  | 15240512 |
|  | 15489334 |
|  | 16204849 |
|  | 16308474 |
|  | 16402859 |
|  | 1647768 |
|  | 16482425 |
|  | 16494995 |
|  | 16685387 |
|  | 16799632 |
|  | 16860930 |
|  | 16888811 |
|  | 16909106 |
|  | 16917513 |
|  | 16924229 |
|  | 16924240 |
|  | 1694291 |
|  | 16963839 |
|  | 16969087 |
|  | 16973168 |
|  | 16983338 |
|  | 16988471 |
|  | 16988840 |
|  | 1699228 |
|  | 16996204 |
|  | 17001315 |
|  | 17012841 |
|  | 17041757 |
|  | 1705009 |
|  | 17084966 |
|  | 17085012 |
|  | 17097318 |
|  | 17098746 |
|  | 17102589 |
|  | 17105820 |
|  | 17106238 |
|  | 17106255 |
|  | 17106521 |
|  | 17106522 |
|  | 17107393 |
|  | 17113101 |
|  | 17113725 |
|  | 17116689 |
|  | 17119852 |
|  | 17121821 |
|  | 17130833 |
|  | 17130838 |
|  | 17135243 |
|  | 17146433 |
|  | 17151828 |
|  | 17151932 |
|  | 17158854 |
|  | 17158927 |
|  | 17170702 |
|  | 17170736 |
|  | 17172869 |
|  | 17174923 |
|  | 17177838 |
|  | 17186020 |
|  | 17198452 |
|  | 17200338 |
|  | 17203247 |
|  | 17216584 |
|  | 17230496 |
|  | 17234448 |
|  | 17234468 |
|  | 17234915 |
|  | 17237848 |
|  | 17242401 |
|  | 17259658 |
|  | 17264218 |
|  | 17264673 |
|  | 17268548 |
|  | 17269744 |
|  | 17270093 |
|  | 17272277 |
|  | 17276397 |
|  | 17280505 |
|  | 17284368 |
|  | 17289842 |
|  | 17289876 |
|  | 17290307 |
|  | 17294448 |
|  | 17298945 |
|  | 17301692 |
|  | 17302414 |
|  | 17308083 |
|  | 17310253 |
|  | 17310847 |
|  | 17310850 |
|  | 17317670 |
|  | 17317671 |
|  | 17317953 |
|  | 17319790 |
|  | 17328050 |
|  | 17332323 |
|  | 17332504 |
|  | 17336302 |
|  | 17339337 |
|  | 17349958 |
|  | 17349959 |
|  | 17350573 |
|  | 17350822 |
|  | 17351129 |
|  | 17353266 |
|  | 17354237 |
|  | 17360476 |
|  | 17360541 |
|  | 17361096 |
|  | 17363555 |
|  | 17364023 |
|  | 17371838 |
|  | 17372198 |
|  | 17374967 |
|  | 1737852 |
|  | 17383050 |
|  | 17387044 |
|  | 17387280 |
|  | 17387621 |
|  | 17393088 |
|  | 17395153 |
|  | 17397394 |
|  | 17397945 |
|  | 17401423 |
|  | 17401424 |
|  | 17401425 |
|  | 17401427 |
|  | 17401428 |
|  | 17401429 |
|  | 17401430 |
|  | 17401431 |
|  | 17401432 |
|  | 17403527 |
|  | 17406354 |
|  | 17409384 |
|  | 17409824 |
|  | 17417627 |
|  | 17426708 |
|  | 17428325 |
|  | 17434128 |
|  | 17436385 |
|  | 17438265 |
|  | 17447881 |
|  | 17460193 |
|  | 17460194 |
|  | 17461446 |
|  | 17474797 |
|  | 17478762 |
|  | 17482142 |
|  | 17488820 |
|  | 17490733 |
|  | 17499812 |
|  | 17504383 |
|  | 17510313 |
|  | 17511743 |
|  | 17516921 |
|  | 17518987 |
|  | 17534149 |
|  | 17536310 |
|  | 17538794 |
|  | 17540598 |
|  | 17540599 |
|  | 17544001 |
|  | 17557566 |
|  | 17564309 |
|  | 17564316 |
|  | 17567906 |
|  | 17575952 |
|  | 17595776 |
|  | 17612495 |
|  | 17616658 |
|  | 17616682 |
|  | 17642181 |
|  | 1848668 |
|  | 1868473 |
|  | 1905840 |
|  | 1999338 |
|  | 2141171 |
|  | 2156209 |
|  | 2263646 |
|  | 7682763 |
|  | 7773777 |
|  | 7878469 |
|  | 7887414 |
|  | 8013454 |
|  | 8023159 |
|  | 8125298 |
|  | 8266092 |
|  | 8336944 |
|  | 8402617 |
|  | 8619474 |
|  | 8632903 |
|  | 8632915 |
|  | 8829627 |
|  | 8829653 |
|  | 9101296 |
|  | 9110174 |
|  | 9321402 |
|  | 9419979 |
|  | 9450901 |
|  | 10373532 |
|  | 10377173 |
|  | 10894779 |
|  | 11106803 |
|  | 11152481 |
|  | 11298357 |
|  | 11551697 |
|  | 11672522 |
|  | 12101245 |
|  | 12810584 |
|  | 14585359 |
|  | 14991053 |
|  | 15289456 |
|  | 15674344 |
|  | 15989966 |
|  | 16021178 |
|  | 16177095 |
|  | 16785989 |
|  | 16862169 |
|  | 16964282 |
|  | 16990847 |
|  | 17000755 |
|  | 17000767 |
|  | 17032926 |
|  | 17051329 |
|  | 17072342 |
|  | 17079275 |
|  | 17093197 |
|  | 17109898 |
|  | 17116431 |
|  | 17123502 |
|  | 17130828 |
|  | 17130840 |
|  | 17139084 |
|  | 17145779 |
|  | 17158931 |
|  | 17158963 |
|  | 17160013 |
|  | 17160015 |
|  | 17170138 |
|  | 17170753 |
|  | 17170761 |
|  | 17208258 |
|  | 17210642 |
|  | 17212342 |
|  | 17216419 |
|  | 17218959 |
|  | 17220276 |
|  | 17222797 |
|  | 17227869 |
|  | 17234766 |
|  | 17244625 |
|  | 17257424 |
|  | 17257586 |
|  | 17283183 |
|  | 17289567 |
|  | 17290223 |
|  | 17292827 |
|  | 17299132 |
|  | 17306224 |
|  | 17312011 |
|  | 17325035 |
|  | 17326724 |
|  | 17327688 |
|  | 17332334 |
|  | 17334357 |
|  | 17346710 |
|  | 17363588 |
|  | 17363591 |
|  | 17363841 |
|  | 17369354 |
|  | 17374722 |
|  | 17420250 |
|  | 17426250 |
|  | 17441198 |
|  | 17470554 |
|  | 17470788 |
|  | 17476307 |
|  | 17483310 |
|  | 17483459 |
|  | 17510390 |
|  | 17523849 |
|  | 17525270 |
|  | 17525272 |
|  | 17533180 |
|  | 17553900 |
|  | 17554337 |
|  | 17575953 |
|  | 17576930 |
|  | 17660247 |
|  | 2145148 |
|  | 3006031 |
|  | 3329909 |
|  | 7566154 |
|  | 9054499 |
|  | 9811461 |
|  | 16817227 |
|  | 17234627 |
|  | 17368433 |
|  | 17418105 |
|  | 17540029 |
|  | 8663025 |
|  | 15756520 |
|  | 16448698 |
|  | 16690525 |
|  | 16696307 |
|  | 16897432 |
|  | 17059853 |
|  | 17065198 |
|  | 17096406 |
|  | 17180249 |
|  | 17186536 |
|  | 17208332 |
|  | 17224235 |
|  | 17291568 |
|  | 17310983 |
|  | 17339230 |
|  | 17374954 |
|  | 17380154 |
|  | 17383977 |
|  | 17388661 |
|  | 17390010 |
|  | 17390041 |
|  | 17404594 |
|  | 17406801 |
|  | 17417775 |
|  | 17426439 |
|  | 17426704 |
|  | 17429838 |
|  | 17437012 |
|  | 17452332 |
|  | 17465990 |
|  | 17488735 |
|  | 17490723 |
|  | 17494866 |
|  | 17495352 |
|  | 17498291 |
|  | 17500067 |
|  | 17504512 |
|  | 17504517 |
|  | 17513604 |
|  | 17513613 |
|  | 17515610 |
|  | 17523278 |
|  | 17535811 |
|  | 17537232 |
|  | 17544403 |
|  | 17545525 |
|  | 17545546 |
|  | 17548471 |
|  | 17549665 |
|  | 17554199 |
|  | 17555331 |
|  | 17567589 |
|  | 17576382 |
|  | 17581420 |
|  | 17581423 |
|  | 17584297 |
|  | 17586686 |
|  | 17598983 |
|  | 17599062 |
|  | 17606709 |
|  | 17607506 |
|  | 17609875 |
|  | 17613764 |
|  | 17616578 |
|  | 17620186 |
|  | 17620598 |
|  | 17628545 |
|  | 17630856 |
|  | 17631738 |
|  | 17634554 |
|  | 17634560 |
|  | 17638885 |
|  | 17640893 |
|  | 17652955 |
|  | 17659731 |
|  | 17671422 |
|  | 17695524 |
|  | 17699779 |
|  | 17701916 |
|  | 17707234 |
|  | 17709727 |
|  | 17713095 |
|  | 17786186 |
|  | 17805299 |
|  | 10435637 |
|  | 11420707 |
|  | 14985341 |
|  | 15665276 |
|  | 15888486 |
|  | 15902973 |
|  | 16118324 |
|  | 16801400 |
|  | 17068116 |
|  | 17215515 |
|  | 17219434 |
|  | 17388726 |
|  | 17443686 |
|  | 17476283 |
|  | 17526734 |
|  | 17535972 |
|  | 17555992 |
|  | 17557176 |
|  | 17606864 |
|  | 17607363 |
|  | 17611229 |
|  | 17626182 |
|  | 17637672 |
|  | 17671189 |
|  | 17675519 |
|  | 17676035 |
|  | 17699762 |
|  | 17707235 |
|  | 17726827 |
|  | 17804719 |
|  | 17875692 |
|  | 17431217 |
|  | 17442733 |

---

|  |  |
| --- | --- |
| CellType | glandular cells |
|  | squamous epithelial cells |
|  | cells in seminiferus ducts |
|  | epidermal cells |
|  | Leydig Cells |
|  | respiratory epithelial cells |
|  | cells in glomeruli |
|  | germinal center cells |
|  | myocytes |
|  | follicle cells |
|  | cells in tubules |
|  | non-germinal center cells |
|  | ovarian stroma cells |
|  | glial cells |
|  | lymphoid tissue |
|  | exocrine glandular cells |
|  | neuronal cells |
|  | hematopoietic cells |
|  | urothelial cells |
|  | smooth muscle cells |
|  | islets of Langerhans |
|  | bile duct cells |
|  | cells in endometrial stroma |
|  | cells in red pulp |
|  | Hepatocytes |
|  | cells in white pulp |
|  | decidual cells |
|  | Macrophages |
|  | cells in granular layer |
|  | trophoblastic cells |
|  | Pneumocytes |
|  | cells in molecular layer |
|  | Purkinje Cells |

---

|  |  |
| --- | --- |
| Tissue | uterus, pre-menopause |
|  | stomach, upper |
|  | vagina |
|  | testis |
|  | rectum |
|  | vulva/anal skin |
|  | Salivary gland |
|  | nasopharynx |
|  | thyroid gland |
|  | seminal vesicle |
|  | oral mucosa |
|  | Kidney |
|  | Adrenal gland |
|  | tonsil |
|  | skeletal muscle |
|  | Ovary |
|  | cervix, uterine |
|  | Appendix |
|  | Skin |
|  | lateral ventricle |
|  | colon |
|  | small intestine |
|  | Pancreas |
|  | duodenum |
|  | bone marrow |
|  | urinary bladder |
|  | smooth muscle |
|  | Liver |
|  | epididymis |
|  | breast |
|  | uterus, post-menopause |
|  | spleen |
|  | parathyroid gland |
|  | esophagus |
|  | bronchus |
|  | Placenta |
|  | Lung |
|  | fallopian tube |
|  | Cerebellum |
|  | stomach, lower |
|  | gall bladder |
|  | Prostate |
|  | lymph node |
|  | heart muscle |
|  | hippocampus |
|  | cerebral cortex |

---

|  |  |
| --- | --- |
| Organism | Homo sapiens {Organism urn:agi-taxid:9606} |
|  | Mus musculus {Organism urn:agi-taxid:10090} |
|  | Rattus norvegicus {Organism urn:agi-taxid:10116} |
|  | Mus musculus |
|  | Homo sapiens |
|  | Rattus norvegicus |
|  | Bos taurus |
|  | Danio rerio |
|  | murine |
|  | rat |
|  | mouse |
|  | Human |

---

|  |  |
| --- | --- |
| Mouse chromosome position | 11 42.83 cM |
|  | 11 39.0 cM |

---

|  |  |
| --- | --- |
| OMIM ID | 191170 |
|  | 202300 |
|  | 614740 |
|  | 618165 |
|  | 114500 |
|  | 260350 |
|  | 260500 |
|  | 114480 |
|  | 137800 |
|  | 114550 |
|  | 151623 |
|  | 607107 |
|  | 259500 |
|  | 161550 |
|  | OMIM:260350 |
|  | OMIM:114500 |
|  | OMIM:114550 |
|  | OMIM:161550 |
|  | OMIM:114480 |
|  | OMIM:151623 |
|  | OMIM:191170 |
|  | OMIM:202300 |

---

|  |  |
| --- | --- |
| Rat chromosome position | 10q24 |

---

|  |  |
| --- | --- |
| Hugo ID | 11998 |
|  | HGNC:11998 |

---

|  |  |
| --- | --- |
| Human chromosome position | 17p13.1 |

---

|  |  |
| --- | --- |
| Swiss-Prot Accession | K7PPA8 |
|  | P04637 |
|  | Q53GA5 |
|  | H2EHT1 |
|  | A0A087X1Q1 |
|  | A0A087WXZ1 |
|  | A0A087WT22 |
|  | P04637.4 |
|  | P02340 |
|  | Q80ZA1 |
|  | Q3UGQ1 |
|  | Q549C9 |
|  | P02340.4 |
|  | P10361 |
|  | P10361.1 |
|  | Q9QUP3 |
|  | O09168 |
|  | Q4KMA9 |
|  | Q66HM0 |
|  | Q15086 |
|  | Q15087 |
|  | Q15088 |
|  | Q16535 |
|  | Q16807 |
|  | Q16808 |
|  | Q16809 |
|  | Q16810 |
|  | Q16811 |
|  | Q16848 |
|  | Q2XN98 |
|  | Q3LRW1 |
|  | Q3LRW2 |
|  | Q3LRW3 |
|  | Q3LRW4 |
|  | Q3LRW5 |
|  | Q86UG1 |
|  | Q8J016 |
|  | Q99659 |
|  | Q9BTM4 |
|  | Q9HAQ8 |
|  | Q9NP68 |
|  | Q9NPJ2 |
|  | Q9NZD0 |
|  | Q9UBI2 |
|  | Q9UQ61 |
|  | P02340.3 |
|  | Q0PKT5 |
|  | Q0ZAJ9 |
|  | Q0ZAK0 |
|  | Q0ZAK1 |
|  | Q19RW5 |
|  | Q19RW6 |
|  | Q1HGV0 |
|  | Q1HGV1 |
|  | Q1HGV2 |
|  | Q1HGV3 |
|  | Q1MSW8 |
|  | Q1MSW9 |
|  | Q1MSX0 |
|  | Q27HK5 |
|  | Q2XSC7 |
|  | Q4FEW1 |
|  | Q5U0E4 |
|  | Q6IT77 |
|  | Q761V2 |
|  | O70366 |
|  | Q64451 |
|  | Q6TDG9 |
|  | Q8C526 |
|  | Q91XH8 |
|  | Q925E2 |
|  | Q9ER40 |
|  | Q9JLE0 |
|  | Q9JLE1 |
|  | Q9EPP9 |
|  | Q9EQL0 |
|  | Q9ERP8 |
|  | Q9ERP9 |
|  | Q9JLD8 |
|  | Q9JLD9 |
|  | P53\_HUMAN |
|  | P53\_MOUSE |
|  | P53\_RAT |
|  | Q3LRW1\_HUMAN |
|  | Q3LRW2\_HUMAN |
|  | Q3LRW3\_HUMAN |
|  | Q3LRW4\_HUMAN |
|  | Q549C9\_MOUSE |
|  | Q80ZA1\_MOUSE |
|  | Q5F217 |
|  | Q5F218 |
|  | Q6DUC3\_HDV |
|  | TALA\_SV40 |

---

|  |  |
| --- | --- |
| PIR ID | A22739 |
|  | S38824 |
|  | S02192 |
|  | A25224 |

---

|  |  |
| --- | --- |
| GenBank ID | NC\_000017 |
|  | NM\_001126112 |
|  | NP\_001119584 |
|  | NM\_000546 |
|  | NP\_000537 |
|  | NM\_001126113 |
|  | NP\_001119585 |
|  | NM\_001126114 |
|  | NP\_001119586 |
|  | NM\_001126118 |
|  | NP\_001119590 |
|  | NM\_001276761 |
|  | NP\_001263690 |
|  | NM\_001276760 |
|  | NP\_001263689 |
|  | NM\_001276695 |
|  | NP\_001263624 |
|  | NM\_001276696 |
|  | NP\_001263625 |
|  | NM\_001126115 |
|  | NP\_001119587 |
|  | NM\_001126117 |
|  | NP\_001119589 |
|  | NM\_001126116 |
|  | NP\_001119588 |
|  | NM\_001276697 |
|  | NP\_001263626 |
|  | NM\_001276699 |
|  | NP\_001263628 |
|  | NM\_001276698 |
|  | NP\_001263627 |
|  | NG\_017013 |
|  | AB118156 |
|  | BAD11806 |
|  | AB511810 |
|  | BAJ52715 |
|  | AB699004 |
|  | BAL63031 |
|  | AB699689 |
|  | BAL63213 |
|  | AB700556 |
|  | BAL63820 |
|  | AC007421 |
|  | AC087388 |
|  | AF020387 |
|  | AF066082 |
|  | AAC27931 |
|  | AF209128 |
|  | AAF36354 |
|  | AF209129 |
|  | AAF36355 |
|  | AF209130 |
|  | AAF36356 |
|  | AF209131 |
|  | AAF36357 |
|  | AF209132 |
|  | AAF36358 |
|  | AF209133 |
|  | AAF36359 |
|  | AF209134 |
|  | AAF36360 |
|  | AF209135 |
|  | AAF36361 |
|  | AF209136 |
|  | AAF36362 |
|  | AF209137 |
|  | AAF36363 |
|  | AF209138 |
|  | AAF36364 |
|  | AF209139 |
|  | AAF36365 |
|  | AF209140 |
|  | AAF36366 |
|  | AF209141 |
|  | AAF36367 |
|  | AF209142 |
|  | AAF36368 |
|  | AF209143 |
|  | AAF36369 |
|  | AF209144 |
|  | AAF36370 |
|  | AF209145 |
|  | AAF36371 |
|  | AF209146 |
|  | AAF36372 |
|  | AF209147 |
|  | AAF36373 |
|  | AF209148 |
|  | AAF36374 |
|  | AF209149 |
|  | AAF36375 |
|  | AF209150 |
|  | AAF36376 |
|  | AF209151 |
|  | AAF36377 |
|  | AF209152 |
|  | AAF36378 |
|  | AF209153 |
|  | AAF36379 |
|  | AF209154 |
|  | AAF36380 |
|  | AF209155 |
|  | AAF36381 |
|  | AF209156 |
|  | AAF36382 |
|  | AF240684 |
|  | AAK76358 |
|  | AF240685 |
|  | AAK76359 |
|  | AH002918 |
|  | AH002919 |
|  | AAA59988 |
|  | AH007665 |
|  | AAD28535 |
|  | AH007667 |
|  | AAD28628 |
|  | AH009228 |
|  | AAF63442 |
|  | AAF63443 |
|  | AM183782 |
|  | AY270155 |
|  | AAP30003 |
|  | AY359814 |
|  | AAR13239 |
|  | AY390341 |
|  | AAQ90158 |
|  | AY838896 |
|  | AAV80424 |
|  | CH471108 |
|  | EAW90140 |
|  | EAW90141 |
|  | EAW90142 |
|  | EAW90143 |
|  | EAW90144 |
|  | EAW90145 |
|  | DQ075362 |
|  | AAZ05887 |
|  | DQ485151 |
|  | ABF61799 |
|  | DQ486036 |
|  | ABF51616 |
|  | DQ486037 |
|  | DQ486038 |
|  | ABF51617 |
|  | DQ486039 |
|  | ABF51618 |
|  | DQ486040 |
|  | ABF51619 |
|  | DQ812984 |
|  | ABG67755 |
|  | EF178466 |
|  | ABM69022 |
|  | EF178467 |
|  | ABM69023 |
|  | EF178468 |
|  | ABM69024 |
|  | EF178469 |
|  | ABM69025 |
|  | EF178470 |
|  | ABM69026 |
|  | EF178471 |
|  | ABM69027 |
|  | EF445551 |
|  | ABO30992 |
|  | EF445552 |
|  | ABO30993 |
|  | EF445553 |
|  | ABO30994 |
|  | EF445554 |
|  | ABO30995 |
|  | EF445555 |
|  | ABO30996 |
|  | EF445556 |
|  | ABO30997 |
|  | EF445557 |
|  | ABO30998 |
|  | EF445558 |
|  | ABO30999 |
|  | EF445559 |
|  | ABO31000 |
|  | EF445560 |
|  | ABO31001 |
|  | EF445561 |
|  | ABO31002 |
|  | EF445562 |
|  | ABO31003 |
|  | EF445563 |
|  | ABO31004 |
|  | EF445564 |
|  | ABO31005 |
|  | EF445565 |
|  | ABO31006 |
|  | EF445566 |
|  | ABO31007 |
|  | EF445567 |
|  | ABO31008 |
|  | EF445568 |
|  | ABO31009 |
|  | EF445569 |
|  | ABO31010 |
|  | EF445570 |
|  | ABO31011 |
|  | EF445571 |
|  | ABO31012 |
|  | EF445572 |
|  | ABO28385 |
|  | EF445573 |
|  | ABO28386 |
|  | EF445574 |
|  | ABO28387 |
|  | EF445575 |
|  | ABO28388 |
|  | EF445576 |
|  | ABO28389 |
|  | EF445577 |
|  | ABO28390 |
|  | EF445578 |
|  | ABO28391 |
|  | EF445579 |
|  | ABO28392 |
|  | EF445580 |
|  | ABO28393 |
|  | EF445581 |
|  | ABO28394 |
|  | EF445582 |
|  | ABO28395 |
|  | EF445583 |
|  | ABO28396 |
|  | EF445584 |
|  | ABO28397 |
|  | EF445585 |
|  | ABO28398 |
|  | EF445586 |
|  | ABO28399 |
|  | EF445587 |
|  | ABO28400 |
|  | EF445588 |
|  | ABO28401 |
|  | EF445589 |
|  | ABO28402 |
|  | EF445590 |
|  | ABO28403 |
|  | EF445591 |
|  | ABO28404 |
|  | EF445592 |
|  | ABO28405 |
|  | EF445593 |
|  | ABO28406 |
|  | EF445594 |
|  | ABO28407 |
|  | EF445595 |
|  | ABO28408 |
|  | EF445596 |
|  | ABO28409 |
|  | EF445597 |
|  | ABO28410 |
|  | EF445598 |
|  | ABO28411 |
|  | EF445599 |
|  | ABO28412 |
|  | EF445600 |
|  | ABO28413 |
|  | EF445601 |
|  | ABO28414 |
|  | EF445602 |
|  | ABO28415 |
|  | EF445603 |
|  | ABO28416 |
|  | EF445604 |
|  | ABO28417 |
|  | EF445605 |
|  | ABO28418 |
|  | EF445606 |
|  | ABO28419 |
|  | EF445607 |
|  | ABO28420 |
|  | EF445608 |
|  | ABO28421 |
|  | EF445609 |
|  | ABO28422 |
|  | EF445610 |
|  | ABO28423 |
|  | EF445611 |
|  | ABO28424 |
|  | EF445612 |
|  | ABO28425 |
|  | EF445613 |
|  | ABO28426 |
|  | EF445614 |
|  | ABO28427 |
|  | EF445615 |
|  | ABO28428 |
|  | EF445616 |
|  | ABO28429 |
|  | EF445617 |
|  | ABO28430 |
|  | EF445618 |
|  | ABO28431 |
|  | EF445619 |
|  | ABO28432 |
|  | EF445620 |
|  | ABO28433 |
|  | EF445621 |
|  | ABO28434 |
|  | EF445622 |
|  | ABO28435 |
|  | EF445623 |
|  | ABO28436 |
|  | EF445624 |
|  | ABO28437 |
|  | EF445625 |
|  | ABO28438 |
|  | EF445626 |
|  | ABO28439 |
|  | EF680843 |
|  | ABV21350 |
|  | EF680844 |
|  | ABV21351 |
|  | EF680845 |
|  | ABV21352 |
|  | EU514493 |
|  | ACA79923 |
|  | EU523098 |
|  | ACA63978 |
|  | EU625581 |
|  | ACD01218 |
|  | EU625584 |
|  | ACD01219 |
|  | EU625589 |
|  | ACD01220 |
|  | EU625590 |
|  | ACD01221 |
|  | EU856039 |
|  | ACF94998 |
|  | EU856040 |
|  | ACF94999 |
|  | EU856041 |
|  | ACF95000 |
|  | EU856042 |
|  | ACF95001 |
|  | EU876946 |
|  | EU876947 |
|  | EU876948 |
|  | EU876949 |
|  | EU876950 |
|  | EU876951 |
|  | EU876952 |
|  | EU876953 |
|  | EU876954 |
|  | EU876955 |
|  | EU876956 |
|  | EU876957 |
|  | EU876958 |
|  | EU876959 |
|  | EU876960 |
|  | EU876961 |
|  | EU876962 |
|  | EU876963 |
|  | EU876964 |
|  | EU876965 |
|  | EU876966 |
|  | EU876967 |
|  | EU876968 |
|  | EU876969 |
|  | EU876970 |
|  | EU876971 |
|  | EU876972 |
|  | EU876973 |
|  | EU876974 |
|  | EU876975 |
|  | EU876976 |
|  | EU876977 |
|  | EU876978 |
|  | EU876979 |
|  | EU876980 |
|  | EU876981 |
|  | EU876982 |
|  | EU876983 |
|  | EU876984 |
|  | EU876985 |
|  | EU876986 |
|  | EU876987 |
|  | EU876988 |
|  | EU876989 |
|  | EU876990 |
|  | EU876991 |
|  | EU876992 |
|  | EU876993 |
|  | EU876994 |
|  | EU876995 |
|  | EU876996 |
|  | EU876997 |
|  | EU876998 |
|  | EU876999 |
|  | EU877000 |
|  | EU877001 |
|  | EU877002 |
|  | EU877003 |
|  | EU877004 |
|  | EU877005 |
|  | EU877006 |
|  | EU877007 |
|  | EU877008 |
|  | EU877009 |
|  | EU877010 |
|  | EU877011 |
|  | EU877012 |
|  | EU877013 |
|  | EU877014 |
|  | EU877015 |
|  | EU877016 |
|  | EU877017 |
|  | EU877018 |
|  | EU877019 |
|  | EU877020 |
|  | EU877021 |
|  | EU877022 |
|  | EU877023 |
|  | EU877024 |
|  | EU877025 |
|  | EU877026 |
|  | EU877027 |
|  | EU877028 |
|  | EU877029 |
|  | EU877030 |
|  | EU877031 |
|  | EU877032 |
|  | EU877033 |
|  | EU877034 |
|  | EU877035 |
|  | EU877036 |
|  | EU877037 |
|  | EU877038 |
|  | EU877039 |
|  | EU877040 |
|  | EU877041 |
|  | EU877042 |
|  | EU877043 |
|  | EU877044 |
|  | EU877045 |
|  | EU877046 |
|  | EU877047 |
|  | EU877048 |
|  | EU877049 |
|  | EU877050 |
|  | EU877051 |
|  | EU877052 |
|  | EU877053 |
|  | EU877054 |
|  | EU877055 |
|  | EU877056 |
|  | EU877057 |
|  | EU877058 |
|  | EU877059 |
|  | EU877060 |
|  | EU877061 |
|  | GU573755 |
|  | ADE43214 |
|  | GU573756 |
|  | ADE43215 |
|  | GU573757 |
|  | ADE43216 |
|  | GU573758 |
|  | ADE43217 |
|  | GU573759 |
|  | ADE43218 |
|  | HM236157 |
|  | ADN42962 |
|  | HQ457168 |
|  | J04238 |
|  | JC337793 |
|  | CDM63359 |
|  | JF923567 |
|  | AEQ16481 |
|  | JF923568 |
|  | JF923569 |
|  | AEQ16482 |
|  | JF923570 |
|  | AEQ16483 |
|  | JF923571 |
|  | AEQ16484 |
|  | JF923572 |
|  | AEQ16485 |
|  | JF923573 |
|  | AEQ16486 |
|  | JQ751320 |
|  | AGA62529 |
|  | JQ751321 |
|  | AGA62530 |
|  | JQ751322 |
|  | AGA62531 |
|  | JQ751323 |
|  | AGA62532 |
|  | JQ751324 |
|  | AGA62533 |
|  | JQ751325 |
|  | AGA62534 |
|  | JQ751326 |
|  | AGA62535 |
|  | JQ751327 |
|  | AGA62536 |
|  | JQ751328 |
|  | AGA62537 |
|  | JQ751329 |
|  | AGA62538 |
|  | JQ751330 |
|  | AGA62539 |
|  | JQ751331 |
|  | AGA62540 |
|  | JQ751332 |
|  | AGA62541 |
|  | JQ751333 |
|  | AGA62542 |
|  | JQ751334 |
|  | AGA62543 |
|  | JQ751335 |
|  | AGA62544 |
|  | JQ751336 |
|  | AGA62545 |
|  | JQ751337 |
|  | AGA62546 |
|  | JQ751338 |
|  | AGA62547 |
|  | JQ751339 |
|  | AGA62548 |
|  | JQ751340 |
|  | AGA62549 |
|  | JQ751341 |
|  | AGA62550 |
|  | JQ751342 |
|  | AGA62551 |
|  | JQ751343 |
|  | AGA62552 |
|  | JQ751344 |
|  | AGA62553 |
|  | JQ751345 |
|  | AGA62554 |
|  | JQ751346 |
|  | AGA62555 |
|  | JQ751347 |
|  | AGA62556 |
|  | JQ751348 |
|  | AGA62557 |
|  | JQ751349 |
|  | AGA62558 |
|  | JQ751350 |
|  | AGA62559 |
|  | JQ751351 |
|  | AGA62560 |
|  | JQ751352 |
|  | AGA62561 |
|  | JQ751353 |
|  | AGA62562 |
|  | JQ751354 |
|  | AGA62563 |
|  | JQ751355 |
|  | AGA62564 |
|  | JQ751356 |
|  | AGA62565 |
|  | JQ751357 |
|  | AGA62566 |
|  | JQ751358 |
|  | AGA62567 |
|  | JQ751359 |
|  | AGA62568 |
|  | JQ751360 |
|  | AGA62569 |
|  | JQ751361 |
|  | AGA62570 |
|  | JQ751362 |
|  | AGA62571 |
|  | JQ751363 |
|  | AGA62572 |
|  | JQ751364 |
|  | AGA62573 |
|  | JQ751365 |
|  | AGA62574 |
|  | JQ751366 |
|  | AGA62575 |
|  | JQ751367 |
|  | AGA62576 |
|  | JQ751368 |
|  | AGA62577 |
|  | JQ751369 |
|  | AGA62578 |
|  | JQ751370 |
|  | AGA62579 |
|  | JQ751371 |
|  | AGA62580 |
|  | JQ751372 |
|  | AGA62581 |
|  | JQ751373 |
|  | AGA62582 |
|  | JQ751374 |
|  | AGA62583 |
|  | JQ751375 |
|  | AGA62584 |
|  | JQ751376 |
|  | AGA62585 |
|  | JQ751377 |
|  | AGA62586 |
|  | JQ751378 |
|  | AGA62587 |
|  | JQ751379 |
|  | AGA62588 |
|  | JQ751380 |
|  | AGA62589 |
|  | JQ751381 |
|  | AGA62590 |
|  | JQ751382 |
|  | AGA62591 |
|  | JQ751383 |
|  | AGA62592 |
|  | JQ751384 |
|  | AGA62593 |
|  | JQ751385 |
|  | AGA62594 |
|  | JQ751386 |
|  | AGA62595 |
|  | JQ751387 |
|  | AGA62596 |
|  | JQ751388 |
|  | AGA62597 |
|  | JQ751389 |
|  | AGA62598 |
|  | JQ751390 |
|  | AGA62599 |
|  | JQ751391 |
|  | AGA62600 |
|  | JQ751392 |
|  | AGA62601 |
|  | JQ751393 |
|  | AGA62602 |
|  | JQ751394 |
|  | AGA62603 |
|  | JQ751395 |
|  | AGA62604 |
|  | JQ751396 |
|  | AGA62605 |
|  | JQ751397 |
|  | AGA62606 |
|  | JQ751398 |
|  | AGA62607 |
|  | JQ751399 |
|  | AGA62608 |
|  | JQ751400 |
|  | AGA62609 |
|  | JQ751401 |
|  | AGA62610 |
|  | JQ751402 |
|  | AGA62611 |
|  | JQ751403 |
|  | AGA62612 |
|  | JQ751404 |
|  | AGA62613 |
|  | JQ751405 |
|  | AGA62614 |
|  | JQ751406 |
|  | AGA62615 |
|  | JQ751407 |
|  | AGA62616 |
|  | JQ751408 |
|  | AGA62617 |
|  | JQ751409 |
|  | AGA62618 |
|  | JQ751410 |
|  | AGA62619 |
|  | JQ751411 |
|  | AGA62620 |
|  | JQ751412 |
|  | AGA62621 |
|  | JQ751413 |
|  | AGA62622 |
|  | JQ751414 |
|  | AGA62623 |
|  | JQ751415 |
|  | AGA62624 |
|  | JQ751416 |
|  | AGA62625 |
|  | JQ751417 |
|  | AGA62626 |
|  | JQ751418 |
|  | AGA62627 |
|  | JQ751419 |
|  | AGA62628 |
|  | JQ751420 |
|  | AGA62629 |
|  | JQ751421 |
|  | AGA62630 |
|  | JQ751422 |
|  | AGA62631 |
|  | JQ751423 |
|  | AGA62632 |
|  | JQ751424 |
|  | AGA62633 |
|  | JQ751425 |
|  | AGA62634 |
|  | JQ751426 |
|  | AGA62635 |
|  | JQ751427 |
|  | AGA62636 |
|  | JQ751428 |
|  | AGA62637 |
|  | JQ751429 |
|  | AGA62638 |
|  | JQ751430 |
|  | AGA62639 |
|  | JQ751431 |
|  | AGA62640 |
|  | JQ751432 |
|  | AGA62641 |
|  | JQ751433 |
|  | AGA62642 |
|  | JQ751434 |
|  | AGA62643 |
|  | JQ751435 |
|  | AGA62644 |
|  | JQ751436 |
|  | AGA62645 |
|  | JQ751437 |
|  | AGA62646 |
|  | JQ751438 |
|  | AGA62647 |
|  | JQ751439 |
|  | AGA62648 |
|  | JQ751440 |
|  | AGA62649 |
|  | JQ751441 |
|  | AGA62650 |
|  | JQ751442 |
|  | AGA62651 |
|  | JQ751443 |
|  | AGA62652 |
|  | JQ751444 |
|  | AGA62653 |
|  | JQ751445 |
|  | AGA62654 |
|  | JQ751446 |
|  | AGA62655 |
|  | JQ751447 |
|  | AGA62656 |
|  | JQ751448 |
|  | AGA62657 |
|  | JQ751449 |
|  | AGA62658 |
|  | JQ751450 |
|  | AGA62659 |
|  | JQ751451 |
|  | AGA62660 |
|  | JQ751452 |
|  | AGA62661 |
|  | JQ751453 |
|  | AGA62662 |
|  | JQ751454 |
|  | AGA62663 |
|  | JQ751455 |
|  | AGA62664 |
|  | JQ751456 |
|  | AGA62665 |
|  | JQ751457 |
|  | AGA62666 |
|  | JQ751458 |
|  | AGA62667 |
|  | JQ751459 |
|  | AGA62668 |
|  | JQ751460 |
|  | AGA62669 |
|  | JQ751461 |
|  | AGA62670 |
|  | JQ751462 |
|  | AGA62671 |
|  | JQ751463 |
|  | AGA62672 |
|  | JQ751464 |
|  | AGA62673 |
|  | JQ751465 |
|  | AGA62674 |
|  | JQ751466 |
|  | AGA62675 |
|  | JQ751467 |
|  | AGA62676 |
|  | JQ751468 |
|  | AGA62677 |
|  | JQ751469 |
|  | AGA62678 |
|  | JQ751470 |
|  | AGA62679 |
|  | JQ751471 |
|  | AGA62680 |
|  | JQ751472 |
|  | AGA62681 |
|  | JQ751473 |
|  | AGA62682 |
|  | JQ751474 |
|  | AGA62683 |
|  | JQ751475 |
|  | AGA62684 |
|  | JQ751476 |
|  | AGA62685 |
|  | JQ751477 |
|  | AGA62686 |
|  | JQ751478 |
|  | AGA62687 |
|  | JQ751479 |
|  | AGA62688 |
|  | JQ751480 |
|  | AGA62689 |
|  | JQ751481 |
|  | AGA62690 |
|  | JQ751482 |
|  | AGA62691 |
|  | JQ751483 |
|  | AGA62692 |
|  | JQ751484 |
|  | AGA62693 |
|  | JQ751485 |
|  | AGA62694 |
|  | JQ751486 |
|  | AGA62695 |
|  | JQ751487 |
|  | AGA62696 |
|  | JQ751488 |
|  | AGA62697 |
|  | JQ751489 |
|  | AGA62698 |
|  | JQ751490 |
|  | AGA62699 |
|  | JQ751491 |
|  | AGA62700 |
|  | JQ751492 |
|  | AGA62701 |
|  | JQ751493 |
|  | AGA62702 |
|  | JQ751494 |
|  | JQ751495 |
|  | AGA62703 |
|  | JQ751496 |
|  | AGA62704 |
|  | JQ751497 |
|  | AGA62705 |
|  | JQ751498 |
|  | AGA62706 |
|  | JQ751499 |
|  | AGA62707 |
|  | JQ751500 |
|  | AGA62708 |
|  | JQ751501 |
|  | AGA62709 |
|  | JQ751502 |
|  | AGA62710 |
|  | JQ751503 |
|  | AGA62711 |
|  | JQ751504 |
|  | AGA62712 |
|  | JQ751505 |
|  | AGA62713 |
|  | JQ751506 |
|  | AGA62714 |
|  | JQ751507 |
|  | AGA62715 |
|  | JQ751508 |
|  | AGA62716 |
|  | JQ751509 |
|  | AGA62717 |
|  | JQ751510 |
|  | AGA62718 |
|  | JQ751511 |
|  | AGA62719 |
|  | JQ751512 |
|  | AGA62720 |
|  | JQ751513 |
|  | AGA62721 |
|  | JQ751514 |
|  | AGA62722 |
|  | JQ751515 |
|  | AGA62723 |
|  | JQ751516 |
|  | AGA62724 |
|  | JQ751517 |
|  | AGA62725 |
|  | JQ751518 |
|  | AGA62726 |
|  | JQ751519 |
|  | AGA62727 |
|  | JQ751520 |
|  | AGA62728 |
|  | JQ751521 |
|  | AGA62729 |
|  | JQ751522 |
|  | AGA62730 |
|  | JQ751523 |
|  | AGA62731 |
|  | JQ751524 |
|  | AGA62732 |
|  | JQ751525 |
|  | AGA62733 |
|  | JQ751526 |
|  | AGA62734 |
|  | JQ751527 |
|  | AGA62735 |
|  | JQ751528 |
|  | AGA62736 |
|  | JQ751529 |
|  | AGA62737 |
|  | JQ751530 |
|  | AGA62738 |
|  | JQ751531 |
|  | AGA62739 |
|  | JQ751532 |
|  | AGA62740 |
|  | JQ751533 |
|  | AGA62741 |
|  | JQ751534 |
|  | AGA62742 |
|  | JQ751535 |
|  | AGA62743 |
|  | JQ751536 |
|  | AGA62744 |
|  | JQ751537 |
|  | AGA62745 |
|  | JQ751538 |
|  | AGA62746 |
|  | JQ751539 |
|  | AGA62747 |
|  | JQ751540 |
|  | AGA62748 |
|  | JQ751541 |
|  | AGA62749 |
|  | JQ751542 |
|  | AGA62750 |
|  | JQ751543 |
|  | AGA62751 |
|  | JQ751544 |
|  | JQ751545 |
|  | AGA62752 |
|  | JQ751546 |
|  | AGA62753 |
|  | JQ751547 |
|  | AGA62754 |
|  | JQ751548 |
|  | AGA62755 |
|  | JQ751549 |
|  | AGA62756 |
|  | JQ751550 |
|  | AGA62757 |
|  | JQ751551 |
|  | AGA62758 |
|  | JQ751552 |
|  | AGA62759 |
|  | JQ751553 |
|  | AGA62760 |
|  | JQ751554 |
|  | AGA62761 |
|  | JQ751555 |
|  | AGA62762 |
|  | JQ751556 |
|  | AGA62763 |
|  | JQ751557 |
|  | AGA62764 |
|  | JQ751558 |
|  | AGA62765 |
|  | JQ751559 |
|  | AGA62766 |
|  | JQ751560 |
|  | AGA62767 |
|  | JQ751561 |
|  | AGA62768 |
|  | JQ751562 |
|  | AGA62769 |
|  | JQ751563 |
|  | AGA62770 |
|  | JQ751564 |
|  | AGA62771 |
|  | JQ751565 |
|  | AGA62772 |
|  | JQ751566 |
|  | AGA62773 |
|  | JQ751567 |
|  | AGA62774 |
|  | JQ751568 |
|  | AGA62775 |
|  | JQ751569 |
|  | AGA62776 |
|  | JQ751570 |
|  | AGA62777 |
|  | JQ751571 |
|  | AGA62778 |
|  | JQ751572 |
|  | AGA62779 |
|  | JQ751573 |
|  | AGA62780 |
|  | JQ751574 |
|  | AGA62781 |
|  | JQ751575 |
|  | AGA62782 |
|  | JQ751576 |
|  | AGA62783 |
|  | JQ751577 |
|  | AGA62784 |
|  | JQ751578 |
|  | AGA62785 |
|  | JQ751579 |
|  | AGA62786 |
|  | JQ751580 |
|  | AGA62787 |
|  | JQ751581 |
|  | AGA62788 |
|  | JQ751582 |
|  | AGA62789 |
|  | JQ751583 |
|  | AGA62790 |
|  | JQ751584 |
|  | AGA62791 |
|  | JQ751585 |
|  | AGA62792 |
|  | JQ751586 |
|  | AGA62793 |
|  | JQ751587 |
|  | AGA62794 |
|  | JQ751588 |
|  | AGA62795 |
|  | JQ751589 |
|  | AGA62796 |
|  | JQ751590 |
|  | AGA62797 |
|  | JQ751591 |
|  | AGA62798 |
|  | JQ751592 |
|  | AGA62799 |
|  | JQ751593 |
|  | AGA62800 |
|  | JQ751594 |
|  | AGA62801 |
|  | JQ751595 |
|  | AGA62802 |
|  | JQ751596 |
|  | AGA62803 |
|  | JQ751597 |
|  | AGA62804 |
|  | JQ751598 |
|  | AGA62805 |
|  | JQ751599 |
|  | AGA62806 |
|  | JQ751600 |
|  | AGA62807 |
|  | JQ751601 |
|  | AGA62808 |
|  | JQ751602 |
|  | AGA62809 |
|  | JQ751603 |
|  | AGA62810 |
|  | JQ751604 |
|  | AGA62811 |
|  | JQ751605 |
|  | AGA62812 |
|  | JQ751606 |
|  | AGA62813 |
|  | JQ751607 |
|  | AGA62814 |
|  | JQ751608 |
|  | AGA62815 |
|  | JQ751609 |
|  | AGA62816 |
|  | JQ751610 |
|  | AGA62817 |
|  | JQ751611 |
|  | AGA62818 |
|  | JQ751612 |
|  | AGA62819 |
|  | JQ751613 |
|  | AGA62820 |
|  | JQ751614 |
|  | AGA62821 |
|  | JQ751615 |
|  | AGA62822 |
|  | JQ751616 |
|  | AGA62823 |
|  | JQ751617 |
|  | AGA62824 |
|  | JQ751618 |
|  | AGA62825 |
|  | JQ751619 |
|  | AGA62826 |
|  | JQ751620 |
|  | AGA62827 |
|  | JQ751621 |
|  | AGA62828 |
|  | JQ751622 |
|  | AGA62829 |
|  | JQ751623 |
|  | AGA62830 |
|  | JQ751624 |
|  | AGA62831 |
|  | JQ751625 |
|  | AGA62832 |
|  | JQ751626 |
|  | AGA62833 |
|  | JQ751627 |
|  | AGA62834 |
|  | JQ751628 |
|  | AGA62835 |
|  | JQ751629 |
|  | AGA62836 |
|  | JQ751630 |
|  | AGA62837 |
|  | JQ751631 |
|  | AGA62838 |
|  | JQ751632 |
|  | AGA62839 |
|  | JQ751633 |
|  | AGA62840 |
|  | JQ751634 |
|  | AGA62841 |
|  | JQ751635 |
|  | AGA62842 |
|  | JQ751636 |
|  | AGA62843 |
|  | JQ751637 |
|  | AGA62844 |
|  | JQ751638 |
|  | AGA62845 |
|  | JQ751639 |
|  | AGA62846 |
|  | JQ751640 |
|  | AGA62847 |
|  | JQ751641 |
|  | AGA62848 |
|  | JQ751642 |
|  | AGA62849 |
|  | JQ751643 |
|  | AGA62850 |
|  | JQ751644 |
|  | AGA62851 |
|  | JQ751645 |
|  | AGA62852 |
|  | JQ751646 |
|  | AGA62853 |
|  | JQ751647 |
|  | AGA62854 |
|  | JQ751648 |
|  | AGA62855 |
|  | JQ751649 |
|  | AGA62856 |
|  | JQ751650 |
|  | AGA62857 |
|  | JQ751651 |
|  | AGA62858 |
|  | JQ751652 |
|  | AGA62859 |
|  | JQ751653 |
|  | AGA62860 |
|  | JQ751654 |
|  | AGA62861 |
|  | JQ751655 |
|  | AGA62862 |
|  | JQ751656 |
|  | AGA62863 |
|  | JQ751657 |
|  | AGA62864 |
|  | JQ751658 |
|  | AGA62865 |
|  | JQ751659 |
|  | AGA62866 |
|  | JQ751660 |
|  | AGA62867 |
|  | JQ751661 |
|  | AGA62868 |
|  | JQ751662 |
|  | AGA62869 |
|  | JQ751663 |
|  | AGA62870 |
|  | JQ751664 |
|  | AGA62871 |
|  | JQ751665 |
|  | AGA62872 |
|  | JQ751666 |
|  | AGA62873 |
|  | JQ751667 |
|  | AGA62874 |
|  | JQ751668 |
|  | AGA62875 |
|  | JQ751669 |
|  | AGA62876 |
|  | JQ751670 |
|  | AGA62877 |
|  | JQ751671 |
|  | AGA62878 |
|  | JQ751672 |
|  | AGA62879 |
|  | JQ751673 |
|  | AGA62880 |
|  | JQ751674 |
|  | AGA62881 |
|  | JQ751675 |
|  | AGA62882 |
|  | JQ751676 |
|  | AGA62883 |
|  | JQ751677 |
|  | AGA62884 |
|  | JQ751678 |
|  | AGA62885 |
|  | JQ751679 |
|  | AGA62886 |
|  | JQ751680 |
|  | AGA62887 |
|  | JQ751681 |
|  | AGA62888 |
|  | JQ751682 |
|  | AGA62889 |
|  | JQ751683 |
|  | AGA62890 |
|  | JQ751684 |
|  | AGA62891 |
|  | JQ751685 |
|  | AGA62892 |
|  | JQ751686 |
|  | AGA62893 |
|  | JQ751687 |
|  | AGA62894 |
|  | JQ751688 |
|  | AGA62895 |
|  | JQ751689 |
|  | AGA62896 |
|  | JQ751690 |
|  | AGA62897 |
|  | JQ751691 |
|  | AGA62898 |
|  | JQ751692 |
|  | AGA62899 |
|  | JQ751693 |
|  | AGA62900 |
|  | JQ751694 |
|  | AGA62901 |
|  | JQ751695 |
|  | AGA62902 |
|  | JQ751696 |
|  | AGA62903 |
|  | JQ751697 |
|  | AGA62904 |
|  | JQ751698 |
|  | AGA62905 |
|  | JQ751699 |
|  | AGA62906 |
|  | JQ751700 |
|  | AGA62907 |
|  | JQ751701 |
|  | AGA62908 |
|  | JQ751702 |
|  | AGA62909 |
|  | JQ751703 |
|  | AGA62910 |
|  | JQ751704 |
|  | AGA62911 |
|  | JQ751705 |
|  | AGA62912 |
|  | JQ751706 |
|  | AGA62913 |
|  | JQ751707 |
|  | AGA62914 |
|  | JQ751708 |
|  | AGA62915 |
|  | JQ751709 |
|  | AGA62916 |
|  | JQ751710 |
|  | AGA62917 |
|  | JQ751711 |
|  | AGA62918 |
|  | JQ751712 |
|  | AGA62919 |
|  | JQ751713 |
|  | AGA62920 |
|  | JQ751714 |
|  | AGA62921 |
|  | JQ751715 |
|  | AGA62922 |
|  | JQ751716 |
|  | AGA62923 |
|  | JQ751717 |
|  | AGA62924 |
|  | JQ751718 |
|  | AGA62925 |
|  | JQ751719 |
|  | AGA62926 |
|  | JQ751720 |
|  | AGA62927 |
|  | JQ751721 |
|  | AGA62928 |
|  | JQ751722 |
|  | AGA62929 |
|  | JQ751723 |
|  | AGA62930 |
|  | JQ751724 |
|  | AGA62931 |
|  | JQ751725 |
|  | AGA62932 |
|  | JQ751726 |
|  | AGA62933 |
|  | JQ751727 |
|  | AGA62934 |
|  | JQ751728 |
|  | AGA62935 |
|  | JQ751729 |
|  | AGA62936 |
|  | JQ751730 |
|  | AGA62937 |
|  | JQ751731 |
|  | AGA62938 |
|  | JQ751732 |
|  | AGA62939 |
|  | JQ751733 |
|  | AGA62940 |
|  | JQ751734 |
|  | AGA62941 |
|  | JQ751735 |
|  | AGA62942 |
|  | JQ751736 |
|  | AGA62943 |
|  | JQ751737 |
|  | AGA62944 |
|  | JQ751738 |
|  | AGA62945 |
|  | JQ751739 |
|  | AGA62946 |
|  | JQ751740 |
|  | AGA62947 |
|  | JQ751741 |
|  | AGA62948 |
|  | JQ751742 |
|  | AGA62949 |
|  | JQ751743 |
|  | AGA62950 |
|  | JQ751744 |
|  | AGA62951 |
|  | JQ751745 |
|  | AGA62952 |
|  | JQ751746 |
|  | AGA62953 |
|  | JQ751747 |
|  | AGA62954 |
|  | JQ751748 |
|  | AGA62955 |
|  | JQ751749 |
|  | AGA62956 |
|  | JQ751750 |
|  | AGA62957 |
|  | JQ751751 |
|  | AGA62958 |
|  | JQ751752 |
|  | AGA62959 |
|  | JQ751753 |
|  | AGA62960 |
|  | JQ751754 |
|  | AGA62961 |
|  | JQ751755 |
|  | AGA62962 |
|  | JQ751756 |
|  | AGA62963 |
|  | JQ751757 |
|  | AGA62964 |
|  | JQ751758 |
|  | AGA62965 |
|  | JQ751759 |
|  | AGA62966 |
|  | JQ751760 |
|  | AGA62967 |
|  | JQ751761 |
|  | AGA62968 |
|  | JQ751762 |
|  | AGA62969 |
|  | JQ751763 |
|  | AGA62970 |
|  | JQ751764 |
|  | AGA62971 |
|  | JQ751765 |
|  | AGA62972 |
|  | JQ751766 |
|  | AGA62973 |
|  | JQ751767 |
|  | AGA62974 |
|  | JQ751768 |
|  | AGA62975 |
|  | JQ751769 |
|  | AGA62976 |
|  | JQ751770 |
|  | AGA62977 |
|  | JQ751771 |
|  | AGA62978 |
|  | JQ751772 |
|  | AGA62979 |
|  | JQ751773 |
|  | AGA62980 |
|  | JQ751774 |
|  | AGA62981 |
|  | JQ751775 |
|  | AGA62982 |
|  | JQ751776 |
|  | AGA62983 |
|  | JQ751777 |
|  | AGA62984 |
|  | JQ751778 |
|  | AGA62985 |
|  | JQ751779 |
|  | AGA62986 |
|  | JQ751780 |
|  | AGA62987 |
|  | JQ751781 |
|  | AGA62988 |
|  | JQ751782 |
|  | AGA62989 |
|  | JQ751783 |
|  | AGA62990 |
|  | JQ751784 |
|  | AGA62991 |
|  | JQ751785 |
|  | AGA62992 |
|  | JQ751786 |
|  | AGA62993 |
|  | JQ751787 |
|  | AGA62994 |
|  | JQ751788 |
|  | AGA62995 |
|  | JQ751789 |
|  | AGA62996 |
|  | JQ751790 |
|  | AGA62997 |
|  | JQ751791 |
|  | AGA62998 |
|  | JQ751792 |
|  | AGA62999 |
|  | JQ751793 |
|  | AGA63000 |
|  | JQ751794 |
|  | AGA63001 |
|  | JQ751795 |
|  | AGA63002 |
|  | JQ751796 |
|  | AGA63003 |
|  | JQ751797 |
|  | AGA63004 |
|  | JQ751798 |
|  | AGA63005 |
|  | JQ751799 |
|  | AGA63006 |
|  | JQ751800 |
|  | AGA63007 |
|  | JQ751801 |
|  | JQ751802 |
|  | AGA63008 |
|  | JQ751803 |
|  | AGA63009 |
|  | JQ751804 |
|  | AGA63010 |
|  | JQ751805 |
|  | AGA63011 |
|  | JQ751806 |
|  | AGA63012 |
|  | JQ751807 |
|  | AGA63013 |
|  | JQ751808 |
|  | AGA63014 |
|  | JQ751809 |
|  | AGA63015 |
|  | JQ751810 |
|  | AGA63016 |
|  | JQ751811 |
|  | AGA63017 |
|  | JQ751812 |
|  | AGA63018 |
|  | JQ751813 |
|  | AGA63019 |
|  | JQ751814 |
|  | AGA63020 |
|  | JQ751815 |
|  | AGA63021 |
|  | JQ751816 |
|  | AGA63022 |
|  | JQ751817 |
|  | AGA63023 |
|  | JQ751818 |
|  | AGA63024 |
|  | JQ751819 |
|  | AGA63025 |
|  | JQ751820 |
|  | AGA63026 |
|  | JQ751821 |
|  | AGA63027 |
|  | JQ751822 |
|  | AGA63028 |
|  | JQ751823 |
|  | AGA63029 |
|  | JQ751824 |
|  | AGA63030 |
|  | JQ751825 |
|  | AGA63031 |
|  | JQ751826 |
|  | AGA63032 |
|  | JQ751827 |
|  | AGA63033 |
|  | JQ751828 |
|  | AGA63034 |
|  | JQ751829 |
|  | AGA63035 |
|  | JQ751830 |
|  | AGA63036 |
|  | JQ751831 |
|  | AGA63037 |
|  | JQ751832 |
|  | AGA63038 |
|  | JQ751833 |
|  | AGA63039 |
|  | JQ751834 |
|  | AGA63040 |
|  | JQ751835 |
|  | AGA63041 |
|  | JQ751836 |
|  | AGA63042 |
|  | JQ751837 |
|  | AGA63043 |
|  | JQ751838 |
|  | AGA63044 |
|  | JQ751839 |
|  | AGA63045 |
|  | JQ751840 |
|  | AGA63046 |
|  | JQ751841 |
|  | AGA63047 |
|  | JQ751842 |
|  | AGA63048 |
|  | JQ751843 |
|  | AGA63049 |
|  | JQ751844 |
|  | AGA63050 |
|  | JQ751845 |
|  | AGA63051 |
|  | JQ751846 |
|  | AGA63052 |
|  | JQ751847 |
|  | AGA63053 |
|  | JQ751848 |
|  | AGA63054 |
|  | JQ751849 |
|  | AGA63055 |
|  | JQ751850 |
|  | AGA63056 |
|  | JQ751851 |
|  | AGA63057 |
|  | JQ751852 |
|  | AGA63058 |
|  | JQ751853 |
|  | AGA63059 |
|  | JQ751854 |
|  | AGA63060 |
|  | JQ751855 |
|  | AGA63061 |
|  | JQ751856 |
|  | AGA63062 |
|  | JQ751857 |
|  | AGA63063 |
|  | JQ751858 |
|  | AGA63064 |
|  | JQ751859 |
|  | AGA63065 |
|  | JQ751860 |
|  | AGA63066 |
|  | JQ751861 |
|  | AGA63067 |
|  | JQ751862 |
|  | AGA63068 |
|  | JQ751863 |
|  | AGA63069 |
|  | JQ751864 |
|  | AGA63070 |
|  | JQ751865 |
|  | AGA63071 |
|  | JQ751866 |
|  | AGA63072 |
|  | JQ751867 |
|  | AGA63073 |
|  | JQ751868 |
|  | AGA63074 |
|  | JQ751869 |
|  | AGA63075 |
|  | JQ751870 |
|  | AGA63076 |
|  | JQ751871 |
|  | AGA63077 |
|  | JQ751872 |
|  | AGA63078 |
|  | JQ751873 |
|  | AGA63079 |
|  | JQ751874 |
|  | AGA63080 |
|  | JQ751875 |
|  | AGA63081 |
|  | JQ751876 |
|  | AGA63082 |
|  | JQ751877 |
|  | AGA63083 |
|  | JQ751878 |
|  | AGA63084 |
|  | JQ751879 |
|  | AGA63085 |
|  | JQ751880 |
|  | AGA63086 |
|  | JQ751881 |
|  | AGA63087 |
|  | JQ751882 |
|  | AGA63088 |
|  | JQ751883 |
|  | AGA63089 |
|  | JQ751884 |
|  | AGA63090 |
|  | JQ751885 |
|  | AGA63091 |
|  | JQ751886 |
|  | AGA63092 |
|  | JQ751887 |
|  | AGA63093 |
|  | JQ751888 |
|  | AGA63094 |
|  | JQ751889 |
|  | AGA63095 |
|  | JQ751890 |
|  | AGA63096 |
|  | JQ751891 |
|  | AGA63097 |
|  | JQ751892 |
|  | AGA63098 |
|  | JQ751893 |
|  | AGA63099 |
|  | JQ751894 |
|  | AGA63100 |
|  | JQ751895 |
|  | AGA63101 |
|  | JQ751896 |
|  | AGA63102 |
|  | JQ751897 |
|  | AGA63103 |
|  | JQ751898 |
|  | AGA63104 |
|  | JQ751899 |
|  | AGA63105 |
|  | JQ751900 |
|  | AGA63106 |
|  | JQ751901 |
|  | AGA63107 |
|  | JQ751902 |
|  | AGA63108 |
|  | JQ751903 |
|  | AGA63109 |
|  | JQ751904 |
|  | AGA63110 |
|  | JQ751905 |
|  | AGA63111 |
|  | JQ751906 |
|  | AGA63112 |
|  | JQ751907 |
|  | AGA63113 |
|  | JQ751908 |
|  | AGA63114 |
|  | JQ751909 |
|  | AGA63115 |
|  | JQ751910 |
|  | AGA63116 |
|  | JQ751911 |
|  | AGA63117 |
|  | JQ751912 |
|  | AGA63118 |
|  | JQ751913 |
|  | AGA63119 |
|  | JQ751914 |
|  | AGA63120 |
|  | JQ751915 |
|  | AGA63121 |
|  | JQ751916 |
|  | AGA63122 |
|  | JQ751917 |
|  | AGA63123 |
|  | JQ751918 |
|  | AGA63124 |
|  | JQ751919 |
|  | AGA63125 |
|  | JQ751920 |
|  | AGA63126 |
|  | JQ751921 |
|  | AGA63127 |
|  | JQ751922 |
|  | AGA63128 |
|  | JQ751923 |
|  | AGA63129 |
|  | JQ751924 |
|  | AGA63130 |
|  | JQ751925 |
|  | AGA63131 |
|  | JQ751926 |
|  | AGA63132 |
|  | JQ751927 |
|  | AGA63133 |
|  | JQ751928 |
|  | AGA63134 |
|  | JQ751929 |
|  | AGA63135 |
|  | JQ751930 |
|  | AGA63136 |
|  | JQ751931 |
|  | AGA63137 |
|  | JQ751932 |
|  | AGA63138 |
|  | JQ751933 |
|  | AGA63139 |
|  | JQ751934 |
|  | AGA63140 |
|  | JQ751935 |
|  | AGA63141 |
|  | JQ751936 |
|  | AGA63142 |
|  | JQ751937 |
|  | AGA63143 |
|  | JQ751938 |
|  | AGA63144 |
|  | JQ751939 |
|  | AGA63145 |
|  | JQ751940 |
|  | AGA63146 |
|  | JQ751941 |
|  | AGA63147 |
|  | JQ751942 |
|  | AGA63148 |
|  | JQ751943 |
|  | AGA63149 |
|  | JQ751944 |
|  | AGA63150 |
|  | JQ751945 |
|  | AGA63151 |
|  | JQ751946 |
|  | AGA63152 |
|  | JQ751947 |
|  | AGA63153 |
|  | JQ751948 |
|  | AGA63154 |
|  | JQ751949 |
|  | AGA63155 |
|  | JQ751950 |
|  | AGA63156 |
|  | JQ751951 |
|  | AGA63157 |
|  | JQ751952 |
|  | AGA63158 |
|  | JQ751953 |
|  | AGA63159 |
|  | JQ751954 |
|  | AGA63160 |
|  | JQ751955 |
|  | AGA63161 |
|  | JQ751956 |
|  | AGA63162 |
|  | JQ751957 |
|  | AGA63163 |
|  | JQ751958 |
|  | AGA63164 |
|  | JQ751959 |
|  | AGA63165 |
|  | JQ751960 |
|  | AGA63166 |
|  | JQ751961 |
|  | AGA63167 |
|  | JQ751962 |
|  | AGA63168 |
|  | JQ751963 |
|  | AGA63169 |
|  | JQ751964 |
|  | AGA63170 |
|  | JQ751965 |
|  | AGA63171 |
|  | JQ751966 |
|  | AGA63172 |
|  | JQ751967 |
|  | AGA63173 |
|  | JQ751968 |
|  | JQ751969 |
|  | AGA63174 |
|  | JQ751970 |
|  | AGA63175 |
|  | JQ751971 |
|  | AGA63176 |
|  | JQ751972 |
|  | AGA63177 |
|  | JQ751973 |
|  | AGA63178 |
|  | JQ751974 |
|  | AGA63179 |
|  | JQ751975 |
|  | AGA63180 |
|  | JQ751976 |
|  | AGA63181 |
|  | JQ751977 |
|  | AGA63182 |
|  | JQ751978 |
|  | AGA63183 |
|  | JQ751979 |
|  | AGA63184 |
|  | JQ751980 |
|  | AGA63185 |
|  | JQ751981 |
|  | AGA63186 |
|  | JQ751982 |
|  | AGA63187 |
|  | JQ751983 |
|  | AGA63188 |
|  | JQ751984 |
|  | AGA63189 |
|  | JQ751985 |
|  | AGA63190 |
|  | JQ751986 |
|  | AGA63191 |
|  | JQ751987 |
|  | AGA63192 |
|  | JQ751988 |
|  | AGA63193 |
|  | JQ751989 |
|  | AGA63194 |
|  | JQ751990 |
|  | AGA63195 |
|  | JQ751991 |
|  | AGA63196 |
|  | JQ751992 |
|  | AGA63197 |
|  | JQ751993 |
|  | AGA63198 |
|  | JQ751994 |
|  | AGA63199 |
|  | JQ751995 |
|  | AGA63200 |
|  | JQ751996 |
|  | AGA63201 |
|  | JQ751997 |
|  | AGA63202 |
|  | JQ751998 |
|  | AGA63203 |
|  | JQ751999 |
|  | AGA63204 |
|  | JQ752000 |
|  | AGA63205 |
|  | JQ752001 |
|  | AGA63206 |
|  | JQ752002 |
|  | AGA63207 |
|  | JQ752003 |
|  | AGA63208 |
|  | JQ752004 |
|  | AGA63209 |
|  | JQ752005 |
|  | AGA63210 |
|  | JQ752006 |
|  | AGA63211 |
|  | JQ752007 |
|  | AGA63212 |
|  | JQ752008 |
|  | AGA63213 |
|  | JQ752009 |
|  | AGA63214 |
|  | JQ752010 |
|  | AGA63215 |
|  | JQ752011 |
|  | AGA63216 |
|  | JQ752012 |
|  | AGA63217 |
|  | JQ752013 |
|  | AGA63218 |
|  | JQ752014 |
|  | AGA63219 |
|  | JQ752015 |
|  | AGA63220 |
|  | JQ752016 |
|  | AGA63221 |
|  | JQ752017 |
|  | AGA63222 |
|  | JQ752018 |
|  | AGA63223 |
|  | JQ752019 |
|  | AGA63224 |
|  | JQ752020 |
|  | AGA63225 |
|  | JQ752021 |
|  | AGA63226 |
|  | JQ752022 |
|  | AGA63227 |
|  | JQ752023 |
|  | AGA63228 |
|  | JQ752024 |
|  | AGA63229 |
|  | JQ752025 |
|  | AGA63230 |
|  | JQ752026 |
|  | AGA63231 |
|  | JQ752027 |
|  | AGA63232 |
|  | JQ752028 |
|  | AGA63233 |
|  | JQ752029 |
|  | AGA63234 |
|  | JQ752030 |
|  | AGA63235 |
|  | JQ752031 |
|  | AGA63236 |
|  | JQ752032 |
|  | AGA63237 |
|  | JQ752033 |
|  | AGA63238 |
|  | JQ752034 |
|  | AGA63239 |
|  | JQ752035 |
|  | AGA63240 |
|  | JQ752036 |
|  | AGA63241 |
|  | JQ752037 |
|  | AGA63242 |
|  | JQ752038 |
|  | AGA63243 |
|  | JQ752039 |
|  | AGA63244 |
|  | JQ752040 |
|  | AGA63245 |
|  | JQ752041 |
|  | AGA63246 |
|  | JQ752042 |
|  | AGA63247 |
|  | JQ752043 |
|  | AGA63248 |
|  | JQ752044 |
|  | AGA63249 |
|  | JQ752045 |
|  | AGA63250 |
|  | JQ752046 |
|  | AGA63251 |
|  | JQ752047 |
|  | AGA63252 |
|  | JQ752048 |
|  | AGA63253 |
|  | JQ752049 |
|  | AGA63254 |
|  | JQ752050 |
|  | AGA63255 |
|  | JQ752051 |
|  | AGA63256 |
|  | JQ752052 |
|  | AGA63257 |
|  | JQ752053 |
|  | AGA63258 |
|  | JQ752054 |
|  | AGA63259 |
|  | JQ752055 |
|  | AGA63260 |
|  | JQ752056 |
|  | AGA63261 |
|  | JQ752057 |
|  | AGA63262 |
|  | JQ752058 |
|  | AGA63263 |
|  | JQ752059 |
|  | AGA63264 |
|  | JQ752060 |
|  | AGA63265 |
|  | JQ752061 |
|  | AGA63266 |
|  | JQ752062 |
|  | AGA63267 |
|  | JQ752063 |
|  | AGA63268 |
|  | JQ752064 |
|  | AGA63269 |
|  | JQ752065 |
|  | AGA63270 |
|  | JQ752066 |
|  | AGA63271 |
|  | JQ752067 |
|  | AGA63272 |
|  | JQ752068 |
|  | AGA63273 |
|  | JQ752069 |
|  | AGA63274 |
|  | JQ752070 |
|  | AGA63275 |
|  | JQ752071 |
|  | AGA63276 |
|  | JQ752072 |
|  | AGA63277 |
|  | JQ752073 |
|  | AGA63278 |
|  | JQ752074 |
|  | AGA63279 |
|  | JQ752075 |
|  | AGA63280 |
|  | JQ752076 |
|  | AGA63281 |
|  | JQ752077 |
|  | AGA63282 |
|  | JQ752078 |
|  | AGA63283 |
|  | JQ752079 |
|  | AGA63284 |
|  | JQ752080 |
|  | AGA63285 |
|  | JQ752081 |
|  | AGA63286 |
|  | JQ752082 |
|  | AGA63287 |
|  | JQ752083 |
|  | AGA63288 |
|  | JQ752084 |
|  | AGA63289 |
|  | JQ752085 |
|  | AGA63290 |
|  | JQ752086 |
|  | AGA63291 |
|  | JQ752087 |
|  | AGA63292 |
|  | JQ752088 |
|  | AGA63293 |
|  | JQ752089 |
|  | AGA63294 |
|  | JQ752090 |
|  | AGA63295 |
|  | JQ752091 |
|  | AGA63296 |
|  | JQ752092 |
|  | AGA63297 |
|  | JQ752093 |
|  | AGA63298 |
|  | JQ752094 |
|  | AGA63299 |
|  | JQ752095 |
|  | AGA63300 |
|  | JQ752096 |
|  | AGA63301 |
|  | JQ752097 |
|  | AGA63302 |
|  | JQ752098 |
|  | AGA63303 |
|  | JQ752099 |
|  | AGA63304 |
|  | JQ752100 |
|  | AGA63305 |
|  | JQ752101 |
|  | AGA63306 |
|  | JQ752102 |
|  | AGA63307 |
|  | JQ752103 |
|  | AGA63308 |
|  | JQ752104 |
|  | AGA63309 |
|  | JQ752105 |
|  | AGA63310 |
|  | JQ752106 |
|  | AGA63311 |
|  | JQ752107 |
|  | AGA63312 |
|  | JQ752108 |
|  | AGA63313 |
|  | JQ752109 |
|  | AGA63314 |
|  | JQ752110 |
|  | AGA63315 |
|  | JQ752111 |
|  | AGA63316 |
|  | JQ752112 |
|  | AGA63317 |
|  | JQ752113 |
|  | AGA63318 |
|  | JQ752114 |
|  | AGA63319 |
|  | JQ752115 |
|  | AGA63320 |
|  | JQ752116 |
|  | AGA63321 |
|  | JQ752117 |
|  | AGA63322 |
|  | JQ752118 |
|  | AGA63323 |
|  | JQ752119 |
|  | AGA63324 |
|  | JQ752120 |
|  | AGA63325 |
|  | JQ752121 |
|  | AGA63326 |
|  | JQ752122 |
|  | AGA63327 |
|  | JQ752123 |
|  | AGA63328 |
|  | JQ752124 |
|  | AGA63329 |
|  | JQ752125 |
|  | AGA63330 |
|  | JQ752126 |
|  | AGA63331 |
|  | JQ752127 |
|  | AGA63332 |
|  | JQ752128 |
|  | AGA63333 |
|  | JQ752129 |
|  | AGA63334 |
|  | JQ752130 |
|  | AGA63335 |
|  | JQ752131 |
|  | AGA63336 |
|  | JQ752132 |
|  | AGA63337 |
|  | JQ752133 |
|  | AGA63338 |
|  | JQ752134 |
|  | AGA63339 |
|  | JQ752135 |
|  | AGA63340 |
|  | JQ752136 |
|  | AGA63341 |
|  | JQ752137 |
|  | AGA63342 |
|  | JQ752138 |
|  | AGA63343 |
|  | JQ752139 |
|  | AGA63344 |
|  | JQ752140 |
|  | AGA63345 |
|  | JQ752141 |
|  | AGA63346 |
|  | JQ752142 |
|  | AGA63347 |
|  | JQ752143 |
|  | AGA63348 |
|  | JQ752144 |
|  | AGA63349 |
|  | JQ752145 |
|  | AGA63350 |
|  | JQ752146 |
|  | AGA63351 |
|  | JQ752147 |
|  | AGA63352 |
|  | JQ752148 |
|  | AGA63353 |
|  | JQ752149 |
|  | AGA63354 |
|  | JQ752150 |
|  | AGA63355 |
|  | JQ752151 |
|  | AGA63356 |
|  | JQ752152 |
|  | AGA63357 |
|  | JQ752153 |
|  | AGA63358 |
|  | JQ752154 |
|  | AGA63359 |
|  | JQ752155 |
|  | AGA63360 |
|  | JQ752156 |
|  | AGA63361 |
|  | JQ752157 |
|  | AGA63362 |
|  | JQ752158 |
|  | AGA63363 |
|  | JQ752159 |
|  | AGA63364 |
|  | JQ752160 |
|  | AGA63365 |
|  | JQ752161 |
|  | AGA63366 |
|  | JQ752162 |
|  | AGA63367 |
|  | JQ752163 |
|  | AGA63368 |
|  | JQ752164 |
|  | AGA63369 |
|  | JQ752165 |
|  | AGA63370 |
|  | JQ752166 |
|  | AGA63371 |
|  | JQ752167 |
|  | AGA63372 |
|  | JQ752168 |
|  | AGA63373 |
|  | JQ752169 |
|  | AGA63374 |
|  | JQ752170 |
|  | AGA63375 |
|  | JQ752171 |
|  | AGA63376 |
|  | JQ752172 |
|  | AGA63377 |
|  | JQ752173 |
|  | AGA63378 |
|  | JQ752174 |
|  | AGA63379 |
|  | JQ752175 |
|  | AGA63380 |
|  | JQ752176 |
|  | AGA63381 |
|  | JQ752177 |
|  | AGA63382 |
|  | JQ752178 |
|  | AGA63383 |
|  | JQ752179 |
|  | AGA63384 |
|  | JQ752180 |
|  | AGA63385 |
|  | JQ752181 |
|  | AGA63386 |
|  | JQ752182 |
|  | AGA63387 |
|  | JQ752183 |
|  | AGA63388 |
|  | JQ752184 |
|  | AGA63389 |
|  | JQ752185 |
|  | AGA63390 |
|  | JQ752186 |
|  | AGA63391 |
|  | JQ752187 |
|  | AGA63392 |
|  | JQ752188 |
|  | AGA63393 |
|  | JQ752189 |
|  | AGA63394 |
|  | JQ752190 |
|  | AGA63395 |
|  | JQ752191 |
|  | AGA63396 |
|  | JQ752192 |
|  | AGA63397 |
|  | JQ752193 |
|  | AGA63398 |
|  | JQ752194 |
|  | AGA63399 |
|  | JQ752195 |
|  | AGA63400 |
|  | JQ752196 |
|  | AGA63401 |
|  | JQ752197 |
|  | AGA63402 |
|  | JQ752198 |
|  | AGA63403 |
|  | JQ752199 |
|  | AGA63404 |
|  | JQ752200 |
|  | AGA63405 |
|  | JQ752201 |
|  | AGA63406 |
|  | JQ752202 |
|  | AGA63407 |
|  | JQ752203 |
|  | AGA63408 |
|  | JQ752204 |
|  | AGA63409 |
|  | JQ752205 |
|  | AGA63410 |
|  | JQ752206 |
|  | AGA63411 |
|  | JQ752207 |
|  | AGA63412 |
|  | JQ752208 |
|  | AGA63413 |
|  | JQ752209 |
|  | AGA63414 |
|  | JQ752210 |
|  | AGA63415 |
|  | JQ752211 |
|  | AGA63416 |
|  | JQ752212 |
|  | AGA63417 |
|  | JQ752213 |
|  | AGA63418 |
|  | JQ752214 |
|  | AGA63419 |
|  | JQ752215 |
|  | AGA63420 |
|  | JQ752216 |
|  | AGA63421 |
|  | JQ752217 |
|  | AGA63422 |
|  | JQ752218 |
|  | AGA63423 |
|  | JQ752219 |
|  | AGA63424 |
|  | JQ752220 |
|  | AGA63425 |
|  | JQ752221 |
|  | AGA63426 |
|  | JQ752222 |
|  | AGA63427 |
|  | JQ752223 |
|  | AGA63428 |
|  | JQ752224 |
|  | AGA63429 |
|  | JQ752225 |
|  | AGA63430 |
|  | JQ752226 |
|  | AGA63431 |
|  | JQ752227 |
|  | AGA63432 |
|  | JQ752228 |
|  | AGA63433 |
|  | JQ752229 |
|  | AGA63434 |
|  | JQ752230 |
|  | AGA63435 |
|  | JQ752231 |
|  | AGA63436 |
|  | JQ752232 |
|  | AGA63437 |
|  | JQ752233 |
|  | AGA63438 |
|  | JQ752234 |
|  | JQ752235 |
|  | JQ752236 |
|  | JQ752237 |
|  | JQ752238 |
|  | JQ752239 |
|  | JQ752240 |
|  | JQ752241 |
|  | JQ752242 |
|  | JQ752243 |
|  | KF572425 |
|  | AHA34178 |
|  | KF572426 |
|  | AHA34179 |
|  | KF572427 |
|  | AHA34180 |
|  | KF572428 |
|  | AHA34181 |
|  | KF572429 |
|  | AHA34182 |
|  | KF572430 |
|  | AHA34183 |
|  | KJ526215 |
|  | AHZ21039 |
|  | KJ526216 |
|  | AHZ21040 |
|  | KJ526217 |
|  | AHZ21041 |
|  | KM488331 |
|  | AIW39812 |
|  | KM597769 |
|  | AIW39813 |
|  | KP987800 |
|  | ALB38992 |
|  | KT584075 |
|  | KT585267 |
|  | KU569918 |
|  | ANT46062 |
|  | KU569919 |
|  | ANT46063 |
|  | KU569920 |
|  | ANT46064 |
|  | KU569921 |
|  | ANT46065 |
|  | KU569922 |
|  | ANT46054 |
|  | KU569923 |
|  | ANT46055 |
|  | KU569924 |
|  | ANT46056 |
|  | KU569925 |
|  | ANT46057 |
|  | KU569926 |
|  | ANT46058 |
|  | KU569927 |
|  | ANT46059 |
|  | KU569928 |
|  | ANT46060 |
|  | KU569929 |
|  | ANT46061 |
|  | KU902450 |
|  | AOX22942 |
|  | KX710182 |
|  | ASU47379 |
|  | M26864 |
|  | MH366303 |
|  | AXU92249 |
|  | MH366304 |
|  | AXU92250 |
|  | MH366305 |
|  | AXU92251 |
|  | MH366306 |
|  | AXU92252 |
|  | MH366307 |
|  | AXU92253 |
|  | MH366308 |
|  | AXU92254 |
|  | MH366309 |
|  | AXU92255 |
|  | MH366310 |
|  | AXU92256 |
|  | MH366311 |
|  | AXU92257 |
|  | MH366312 |
|  | AXU92258 |
|  | MH366313 |
|  | AXU92259 |
|  | MH366314 |
|  | AXU92260 |
|  | MH366315 |
|  | AXU92261 |
|  | MH366316 |
|  | AXU92262 |
|  | MH366317 |
|  | AXU92263 |
|  | MH366318 |
|  | AXU92264 |
|  | MH366319 |
|  | AXU92265 |
|  | MH366320 |
|  | AXU92266 |
|  | MH366321 |
|  | AXU92267 |
|  | MH366322 |
|  | AXU92268 |
|  | MH366323 |
|  | AXU92269 |
|  | MH366324 |
|  | AXU92270 |
|  | MH366325 |
|  | AXU92271 |
|  | MH366326 |
|  | AXU92272 |
|  | MH366327 |
|  | AXU92273 |
|  | MH366328 |
|  | AXU92274 |
|  | MH366329 |
|  | AXU92275 |
|  | MH366330 |
|  | AXU92276 |
|  | MH366331 |
|  | AXU92277 |
|  | MH366332 |
|  | AXU92278 |
|  | MH366333 |
|  | AXU92279 |
|  | MH366334 |
|  | AXU92280 |
|  | MH366335 |
|  | AXU92281 |
|  | MH366336 |
|  | AXU92282 |
|  | MH366337 |
|  | AXU92283 |
|  | MH366338 |
|  | AXU92284 |
|  | MH366339 |
|  | AXU92285 |
|  | MH366340 |
|  | AXU92286 |
|  | MH366341 |
|  | AXU92287 |
|  | MH366342 |
|  | AXU92288 |
|  | MH366343 |
|  | AXU92289 |
|  | MH366344 |
|  | AXU92290 |
|  | MH366345 |
|  | AXU92291 |
|  | MH366346 |
|  | AXU92292 |
|  | MH366347 |
|  | AXU92293 |
|  | MH366348 |
|  | AXU92294 |
|  | MH366349 |
|  | AXU92295 |
|  | MH366350 |
|  | AXU92296 |
|  | MH366351 |
|  | AXU92297 |
|  | MH366352 |
|  | AXU92298 |
|  | MH366353 |
|  | AXU92299 |
|  | MH366354 |
|  | AXU92300 |
|  | MH366355 |
|  | AXU92301 |
|  | MH366356 |
|  | AXU92302 |
|  | MH366357 |
|  | AXU92303 |
|  | MH366358 |
|  | AXU92304 |
|  | MH366359 |
|  | AXU92305 |
|  | MH366360 |
|  | AXU92306 |
|  | MH366361 |
|  | AXU92307 |
|  | MH366362 |
|  | AXU92308 |
|  | MH366363 |
|  | AXU92309 |
|  | MH366364 |
|  | AXU92310 |
|  | MH366365 |
|  | AXU92311 |
|  | MH366366 |
|  | AXU92312 |
|  | MH366367 |
|  | AXU92313 |
|  | MH366368 |
|  | AXU92314 |
|  | MH366369 |
|  | AXU92315 |
|  | MH366370 |
|  | AXU92316 |
|  | MH366371 |
|  | AXU92317 |
|  | MH366372 |
|  | AXU92318 |
|  | MH366373 |
|  | AXU92319 |
|  | MH366374 |
|  | AXU92320 |
|  | MH366375 |
|  | AXU92321 |
|  | MH366376 |
|  | AXU92322 |
|  | MH366377 |
|  | AXU92323 |
|  | MH366378 |
|  | AXU92324 |
|  | MH366379 |
|  | AXU92325 |
|  | MH366380 |
|  | AXU92326 |
|  | MH366381 |
|  | AXU92327 |
|  | MH366382 |
|  | AXU92328 |
|  | MH366383 |
|  | AXU92329 |
|  | MH366384 |
|  | AXU92330 |
|  | MH366385 |
|  | AXU92331 |
|  | MH366386 |
|  | AXU92332 |
|  | MH366387 |
|  | AXU92333 |
|  | MH366388 |
|  | AXU92334 |
|  | MH366389 |
|  | AXU92335 |
|  | MH366390 |
|  | AXU92336 |
|  | MH366391 |
|  | AXU92337 |
|  | MH366392 |
|  | AXU92338 |
|  | MH366393 |
|  | AXU92339 |
|  | MH366394 |
|  | AXU92340 |
|  | MH366395 |
|  | AXU92341 |
|  | MH366396 |
|  | AXU92342 |
|  | MH366397 |
|  | AXU92343 |
|  | MH366398 |
|  | AXU92344 |
|  | MH366399 |
|  | AXU92345 |
|  | MH366400 |
|  | AXU92346 |
|  | MH366404 |
|  | AXU92350 |
|  | MH366405 |
|  | AXU92351 |
|  | MH366420 |
|  | AXU92366 |
|  | MH366421 |
|  | AXU92367 |
|  | MH366426 |
|  | AXU92372 |
|  | MH366428 |
|  | AXU92374 |
|  | MH366438 |
|  | AXU92384 |
|  | MH366445 |
|  | AXU92391 |
|  | MH366446 |
|  | AXU92392 |
|  | MH366447 |
|  | AXU92393 |
|  | MH366448 |
|  | AXU92394 |
|  | MH366449 |
|  | AXU92395 |
|  | MH366450 |
|  | AXU92396 |
|  | MH366451 |
|  | AXU92397 |
|  | MH366452 |
|  | AXU92398 |
|  | MH366453 |
|  | AXU92399 |
|  | MH366454 |
|  | AXU92400 |
|  | MH366455 |
|  | AXU92401 |
|  | MH366456 |
|  | AXU92402 |
|  | MH366457 |
|  | AXU92403 |
|  | MH366458 |
|  | AXU92404 |
|  | MH366459 |
|  | AXU92405 |
|  | MH366460 |
|  | AXU92406 |
|  | MH366461 |
|  | AXU92407 |
|  | MH366462 |
|  | AXU92408 |
|  | MH366463 |
|  | AXU92409 |
|  | MH366464 |
|  | AXU92410 |
|  | MH366465 |
|  | AXU92411 |
|  | MH366466 |
|  | AXU92412 |
|  | MH366467 |
|  | AXU92413 |
|  | MH366468 |
|  | AXU92414 |
|  | MH366469 |
|  | AXU92415 |
|  | MH366470 |
|  | AXU92416 |
|  | MH366471 |
|  | AXU92417 |
|  | MH366472 |
|  | AXU92418 |
|  | MH366473 |
|  | AXU92419 |
|  | MH366475 |
|  | AXU92421 |
|  | MH366476 |
|  | AXU92422 |
|  | MH366477 |
|  | AXU92423 |
|  | MH366478 |
|  | AXU92424 |
|  | MH366479 |
|  | AXU92425 |
|  | MH366480 |
|  | AXU92426 |
|  | MH366481 |
|  | AXU92427 |
|  | MH366482 |
|  | AXU92428 |
|  | MH366483 |
|  | AXU92429 |
|  | S63610 |
|  | S80917 |
|  | S81486 |
|  | U40428 |
|  | AAA83560 |
|  | U63714 |
|  | AAB39322 |
|  | U94788 |
|  | AAC12971 |
|  | X54156 |
|  | CAA38095 |
|  | X92659 |
|  | AB082923 |
|  | BAC16799 |
|  | AF052180 |
|  | AF307851 |
|  | AAG28785 |
|  | AK223026 |
|  | BAD96746 |
|  | AK225838 |
|  | AK297462 |
|  | BAG59884 |
|  | AK297927 |
|  | BAG60244 |
|  | AK303277 |
|  | BAG64357 |
|  | AK312568 |
|  | BAG35463 |
|  | AM076970 |
|  | CAJ28922 |
|  | AM076971 |
|  | CAJ28924 |
|  | AM076972 |
|  | CAJ28923 |
|  | AU131823 |
|  | AY429684 |
|  | AAR10356 |
|  | AY627884 |
|  | AAT40418 |
|  | BC003596 |
|  | AAH03596 |
|  | BT019622 |
|  | AAV38428 |
|  | DA308036 |
|  | DA453049 |
|  | DA932053 |
|  | DC347346 |
|  | DQ186648 |
|  | ABA29753 |
|  | DQ186649 |
|  | ABA29754 |
|  | DQ186650 |
|  | ABA29755 |
|  | DQ186651 |
|  | ABA29756 |
|  | DQ186652 |
|  | ABA29757 |
|  | DQ191317 |
|  | ABB80262 |
|  | DQ263704 |
|  | ABB72446 |
|  | DQ286964 |
|  | ABB80266 |
|  | DQ401704 |
|  | ABD62752 |
|  | DQ485152 |
|  | ABF61800 |
|  | DQ648883 |
|  | DQ648884 |
|  | ABG49480 |
|  | DQ648885 |
|  | ABG49481 |
|  | DQ648886 |
|  | DQ648887 |
|  | ABG49482 |
|  | EF101867 |
|  | ABL09846 |
|  | EF101868 |
|  | ABL09847 |
|  | EF101869 |
|  | ABL09848 |
|  | FJ207420 |
|  | ACI25593 |
|  | HG975427 |
|  | JN214348 |
|  | JN601513 |
|  | AEY81366 |
|  | JN601514 |
|  | AEY81367 |
|  | JN601515 |
|  | AEY81368 |
|  | JN601516 |
|  | AEY81369 |
|  | JN900492 |
|  | AEX20383 |
|  | JQ026207 |
|  | JQ026208 |
|  | JQ026209 |
|  | JQ026210 |
|  | JQ694049 |
|  | AFN61604 |
|  | JQ694050 |
|  | AFN61605 |
|  | JQ694051 |
|  | AFN61606 |
|  | KC820708 |
|  | AGR27803 |
|  | KC820709 |
|  | AGR27804 |
|  | KC820710 |
|  | AGR27805 |
|  | KC820711 |
|  | AGR27806 |
|  | KC820712 |
|  | AGR27807 |
|  | KC820713 |
|  | AGR27808 |
|  | KC820714 |
|  | AGR27809 |
|  | KC820715 |
|  | AGR27810 |
|  | KC820716 |
|  | AGR27811 |
|  | KC820717 |
|  | AGR27812 |
|  | KC820718 |
|  | AGR27813 |
|  | KC820719 |
|  | AGR27814 |
|  | KC820720 |
|  | AGR27815 |
|  | KC820721 |
|  | AGR27816 |
|  | KC820722 |
|  | AGR27817 |
|  | KC820723 |
|  | AGR27818 |
|  | KC820724 |
|  | AGR27819 |
|  | KC820725 |
|  | AGR27820 |
|  | KC820726 |
|  | AGR27821 |
|  | KC820727 |
|  | AGR27822 |
|  | KC820728 |
|  | AGR27823 |
|  | KC820729 |
|  | AGR27824 |
|  | KC820730 |
|  | AGR27825 |
|  | KC820731 |
|  | AGR27826 |
|  | KC820732 |
|  | AGR27827 |
|  | KC820733 |
|  | AGR27828 |
|  | KC820734 |
|  | AGR27829 |
|  | KC820735 |
|  | AGR27830 |
|  | KC820736 |
|  | AGR27831 |
|  | KC820737 |
|  | AGR27832 |
|  | KC820738 |
|  | AGR27833 |
|  | KC820739 |
|  | AGR27834 |
|  | KC820740 |
|  | AGR27835 |
|  | KC820741 |
|  | AGR27836 |
|  | KC820742 |
|  | AGR27837 |
|  | KC820743 |
|  | AGR27838 |
|  | KC820744 |
|  | AGR27839 |
|  | KC820745 |
|  | AGR27840 |
|  | KC820746 |
|  | AGR27841 |
|  | KC820747 |
|  | AGR27842 |
|  | KC820748 |
|  | AGR27843 |
|  | KC820749 |
|  | AGR27844 |
|  | KC820750 |
|  | AGR27845 |
|  | KC820751 |
|  | AGR27846 |
|  | KC820752 |
|  | AGR27847 |
|  | KC820753 |
|  | AGR27848 |
|  | KC820754 |
|  | AGR27849 |
|  | KC820755 |
|  | AGR27850 |
|  | KC820756 |
|  | AGR27851 |
|  | KC820757 |
|  | AGR27852 |
|  | KC820758 |
|  | AGR27853 |
|  | KC820759 |
|  | AGR27854 |
|  | KC820760 |
|  | AGR27855 |
|  | KC820761 |
|  | AGR27856 |
|  | KC820762 |
|  | AGR27857 |
|  | KC820763 |
|  | AGR27858 |
|  | KC820764 |
|  | AGR27859 |
|  | KC820765 |
|  | AGR27860 |
|  | KC820766 |
|  | AGR27861 |
|  | KC820767 |
|  | AGR27862 |
|  | KC820768 |
|  | AGR27863 |
|  | KC820769 |
|  | AGR27864 |
|  | KC820770 |
|  | AGR27865 |
|  | KC820771 |
|  | AGR27866 |
|  | KC820772 |
|  | AGR27867 |
|  | KC820773 |
|  | AGR27868 |
|  | KC820774 |
|  | AGR27869 |
|  | KC820775 |
|  | AGR27870 |
|  | KC820776 |
|  | AGR27871 |
|  | KC820777 |
|  | AGR27872 |
|  | KC820778 |
|  | AGR27873 |
|  | KC820779 |
|  | AGR27874 |
|  | KC820780 |
|  | AGR27875 |
|  | KC820781 |
|  | AGR27876 |
|  | KC820782 |
|  | AGR27877 |
|  | KC820783 |
|  | AGR27878 |
|  | KC820784 |
|  | AGR27879 |
|  | KC820785 |
|  | AGR27880 |
|  | KC820786 |
|  | AGR27881 |
|  | KY907121 |
|  | ASE05873 |
|  | KY907122 |
|  | ASE05874 |
|  | KY907123 |
|  | ASE05875 |
|  | KY907124 |
|  | ASE05876 |
|  | KY907125 |
|  | ASE05877 |
|  | KY907126 |
|  | ASE05878 |
|  | KY907127 |
|  | ASE05879 |
|  | KY907128 |
|  | ASE05880 |
|  | KY907129 |
|  | ASE05881 |
|  | KY907130 |
|  | ASE05882 |
|  | KY907131 |
|  | ASE05883 |
|  | KY907132 |
|  | ASE05884 |
|  | KY907133 |
|  | ASE05885 |
|  | KY907134 |
|  | ASE05886 |
|  | KY907135 |
|  | ASE05887 |
|  | KY907136 |
|  | ASE05888 |
|  | KY907137 |
|  | ASE05889 |
|  | KY907138 |
|  | ASE05890 |
|  | KY907139 |
|  | ASE05891 |
|  | KY907140 |
|  | ASE05892 |
|  | KY907141 |
|  | ASE05893 |
|  | KY907142 |
|  | ASE05894 |
|  | KY907143 |
|  | ASE05895 |
|  | KY907144 |
|  | ASE05896 |
|  | KY907145 |
|  | ASE05897 |
|  | KY907146 |
|  | ASE05898 |
|  | M14694 |
|  | AAA61211 |
|  | M14695 |
|  | AAA61212 |
|  | S66666 |
|  | AAB28601 |
|  | X01405 |
|  | CAA25652 |
|  | X02469 |
|  | CAA26306 |
|  | X60010 |
|  | CAA42625 |
|  | X60011 |
|  | CAA42626 |
|  | X60012 |
|  | CAA42627 |
|  | X60013 |
|  | CAA42628 |
|  | X60014 |
|  | CAA42629 |
|  | X60015 |
|  | CAA42630 |
|  | X60016 |
|  | CAA42631 |
|  | X60017 |
|  | CAA42632 |
|  | X60018 |
|  | CAA42633 |
|  | X60019 |
|  | CAA42634 |
|  | X60020 |
|  | CAA42635 |
|  | P04637 |
|  | NC\_000077 |
|  | NM\_001127233 |
|  | NP\_001120705 |
|  | NM\_011640 |
|  | NP\_035770 |
|  | XM\_006533157 |
|  | XP\_006533220 |
|  | AF074575 |
|  | AAC71763 |
|  | AF074576 |
|  | AAC71764 |
|  | AF190269 |
|  | AAF43275 |
|  | AAF43276 |
|  | AF287146 |
|  | AF367373 |
|  | AAK53397 |
|  | AH004687 |
|  | AL731687 |
|  | CAI52015 |
|  | CAI52016 |
|  | CH466601 |
|  | EDL10505 |
|  | EDL10506 |
|  | EDL10507 |
|  | EF570972 |
|  | ABQ44583 |
|  | EF634061 |
|  | ABR29561 |
|  | JC337791 |
|  | CDM63358 |
|  | M26862 |
|  | S77930 |
|  | AAB21108 |
|  | X00875 |
|  | X00876 |
|  | CAA25420 |
|  | X00877 |
|  | X00878 |
|  | X00879 |
|  | X00880 |
|  | X00881 |
|  | X00882 |
|  | X00883 |
|  | X00884 |
|  | X00885 |
|  | AB017815 |
|  | BAA82339 |
|  | AB017816 |
|  | BAA82340 |
|  | AB020317 |
|  | BAA82343 |
|  | AB021961 |
|  | BAA82344 |
|  | AB221571 |
|  | AF051368 |
|  | AAC05704 |
|  | AF151353 |
|  | AAD39535 |
|  | AF161020 |
|  | AAD44340 |
|  | AI323249 |
|  | AJ297973 |
|  | CAC17147 |
|  | AK079711 |
|  | BAC37729 |
|  | AK083750 |
|  | AK086845 |
|  | AK087834 |
|  | AK138777 |
|  | BAE23775 |
|  | AK147815 |
|  | BAE28156 |
|  | AK190460 |
|  | AK191352 |
|  | AK194492 |
|  | AV450598 |
|  | AY044188 |
|  | AAK94783 |
|  | AY212017 |
|  | AAO60156 |
|  | AY423848 |
|  | AAR00340 |
|  | BC005448 |
|  | AAH05448 |
|  | D63399 |
|  | EU031806 |
|  | ABS81351 |
|  | GU808447 |
|  | KF766124 |
|  | AHH81944 |
|  | M13872 |
|  | AAA39881 |
|  | M13873 |
|  | AAA39882 |
|  | M13874 |
|  | AAA39883 |
|  | U59757 |
|  | AAB03323 |
|  | X00741 |
|  | CAA25323 |
|  | X01237 |
|  | CAA25625 |
|  | P02340 |
|  | NC\_005109 |
|  | XM\_006246594 |
|  | XP\_006246656 |
|  | XM\_006246595 |
|  | XP\_006246657 |
|  | XM\_008767773 |
|  | XP\_008765995 |
|  | NM\_030989 |
|  | NP\_112251 |
|  | XM\_017597018 |
|  | XP\_017452507 |
|  | AC\_000078 |
|  | AAHX01064583 |
|  | AC136563 |
|  | AF190270 |
|  | AAF43277 |
|  | AAF43278 |
|  | AH002222 |
|  | AAA41788 |
|  | AH010014 |
|  | AAG34052 |
|  | CH473948 |
|  | EDM04871 |
|  | EDM04872 |
|  | EDM04873 |
|  | M26863 |
|  | Z93723 |
|  | AF209191 |
|  | AAG35765 |
|  | AY009504 |
|  | AAG35178 |
|  | BC081788 |
|  | AAH81788 |
|  | BC098663 |
|  | AAH98663 |
|  | CV126174 |
|  | FQ232659 |
|  | U90328 |
|  | AAB80959 |
|  | X13058 |
|  | CAA31457 |
|  | P10361 |
|  | XP\_006533221 |
|  | XR\_243566 |
|  | XR\_243565 |
|  | AF136270 |
|  | AF136271 |
|  | DQ895704 |
|  | ABBA01054880 |
|  | XM\_006533158 |
|  | ABM83418 |
|  | S74427 |
|  | S74429 |
|  | S74428 |
|  | S74430 |
|  | S74431 |
|  | AC\_000149 |
|  | XM\_005256778 |
|  | AABR06064390 |
|  | AMYH02032910 |
|  | NC\_018928 |
|  | AF210309 |
|  | AF210308 |
|  | AF210310 |
|  | M13121 |
|  | M13120 |
|  | M13119 |
|  | M13118 |
|  | M13117 |
|  | M13116 |
|  | M13115 |
|  | M13114 |
|  | M13112 |
|  | M13111 |
|  | AAA59987 |
|  | M22897 |
|  | M22896 |
|  | M22898 |
|  | M22895 |
|  | M22894 |
|  | M22888 |
|  | M22887 |
|  | M22882 |
|  | M22881 |
|  | M22884 |
|  | M22883 |
|  | AC\_000033 |
|  | AAHY01094181 |
|  | L07910 |
|  | L07908 |
|  | L07909 |
|  | L07906 |
|  | L07907 |
|  | L07904 |
|  | L07905 |
|  | L07903 |
|  | DQ892492 |
|  | XP\_005256835 |
|  | AAG34053 |
|  | ABM86630 |
|  | AF135120 |
|  | AF135121 |
|  | AF294442 |
|  | AF294441 |
|  | NT\_010718 |
|  | NW\_926584 |
|  | NW\_001838403 |
|  | AC\_000060 |
|  | CR608294 |
|  | CR624912 |
|  | Q0PKT5 |
|  | Q0ZAJ9 |
|  | Q0ZAK0 |
|  | Q0ZAK1 |
|  | Q19RW5 |
|  | Q19RW6 |
|  | Q1HGV0 |
|  | Q1HGV1 |
|  | Q1HGV2 |
|  | Q1HGV3 |
|  | Q1MSW8 |
|  | Q1MSW9 |
|  | Q1MSX0 |
|  | Q27HK5 |
|  | Q2XSC7 |
|  | Q4FEW1 |
|  | Q53GA5 |
|  | Q5U0E4 |
|  | Q6IT77 |
|  | Q761V2 |
|  | NT\_096135 |
|  | NW\_001030477 |
|  | O70366 |
|  | Q3UGQ1 |
|  | Q549C9 |
|  | Q64451 |
|  | Q6TDG9 |
|  | Q80ZA1 |
|  | Q8C526 |
|  | Q91XH8 |
|  | Q925E2 |
|  | Q9ER40 |
|  | Q9JLE0 |
|  | Q9JLE1 |
|  | NW\_047334 |
|  | NW\_001084656 |
|  | Q9EPP9 |
|  | Q9EQL0 |
|  | Q9ERP8 |
|  | Q9ERP9 |
|  | Q9JLD8 |
|  | Q9JLD9 |
|  | Q2XN98 |
|  | Q3LRW1 |
|  | Q3LRW2 |
|  | Q3LRW3 |
|  | Q3LRW4 |
|  | Q3LRW5 |
|  | Q5F217 |
|  | Q5F218 |
|  | Q8BNG5 |
|  | BB828014 |
|  | K03199 |
|  | AAA59989 |
|  | NW\_000039 |
|  | Q66HM0 |
|  | 11321107 |
|  | 11321108 |
|  | 11494110 |
|  | 11528486 |
|  | 123785760 |
|  | 129372 |
|  | 1938365 |
|  | 205952 |
|  | 51858594 |
|  | 56829 |
|  | 68534537 |
|  | 7243330 |
|  | 7243331 |
|  | 81868156 |
|  | 81868322 |
|  | 81868744 |
|  | 81868745 |
|  | 81872498 |
|  | 81890534 |
|  | 81906247 |
|  | Q4KMA9 |
|  | 11342599 |
|  | 123784585 |
|  | 123797709 |
|  | 129371 |
|  | 13242903 |
|  | 13529428 |
|  | 1399878 |
|  | 14039818 |
|  | 15375072 |
|  | 200199 |
|  | 200201 |
|  | 200203 |
|  | 28975327 |
|  | 2961247 |
|  | 37695617 |
|  | 3808323 |
|  | 3808325 |
|  | 5081783 |
|  | 53571 |
|  | 53576 |
|  | 5421808 |
|  | 5421813 |
|  | 5421828 |
|  | 5421850 |
|  | 5524686 |
|  | 58864944 |
|  | 58864945 |
|  | 7243327 |
|  | 7243328 |
|  | 74190719 |
|  | 74213717 |
|  | 74218305 |
|  | 81868746 |
|  | 81868747 |
|  | 81886803 |
|  | 81887965 |
|  | 81890455 |
|  | 81892903 |
|  | 81895466 |
|  | 81896837 |
|  | 81902402 |
|  | 81902710 |
|  | 81906263 |
|  | 81909612 |
|  | 81909613 |
|  | 871421 |
|  | 110164962 |
|  | 110164964 |
|  | 110164967 |
|  | 110338983 |
|  | 11066970 |
|  | 118640554 |
|  | 118640556 |
|  | 118640558 |
|  | 121940396 |
|  | 121940520 |
|  | 121945574 |
|  | 121945611 |
|  | 121945886 |
|  | 121946766 |
|  | 121946897 |
|  | 121946898 |
|  | 121947422 |
|  | 121947423 |
|  | 121947538 |
|  | 121947564 |
|  | 121947565 |
|  | 121947569 |
|  | 121947570 |
|  | 121947714 |
|  | 121947777 |
|  | 121948290 |
|  | 121948291 |
|  | 121948361 |
|  | 121948414 |
|  | 121948988 |
|  | 123998057 |
|  | 129369 |
|  | 13097807 |
|  | 14993572 |
|  | 14993574 |
|  | 1753089 |
|  | 189476 |
|  | 189479 |
|  | 23491729 |
|  | 3041867 |
|  | 30523242 |
|  | 339814 |
|  | 339816 |
|  | 35210 |
|  | 35214 |
|  | 37223061 |
|  | 38049097 |
|  | 38194463 |
|  | 386994 |
|  | 42821410 |
|  | 4731632 |
|  | 4732147 |
|  | 48094187 |
|  | 506433 |
|  | 506435 |
|  | 506437 |
|  | 506439 |
|  | 506441 |
|  | 506443 |
|  | 506445 |
|  | 506447 |
|  | 506449 |
|  | 506451 |
|  | 506453 |
|  | 54696112 |
|  | 56157803 |
|  | 62897613 |
|  | 642241 |
|  | 70611298 |
|  | 7107285 |
|  | 7107287 |
|  | 7107289 |
|  | 7107291 |
|  | 7107293 |
|  | 7107295 |
|  | 7107297 |
|  | 7107299 |
|  | 7107301 |
|  | 7107325 |
|  | 7107327 |
|  | 7107329 |
|  | 7107331 |
|  | 7107333 |
|  | 7107335 |
|  | 7107337 |
|  | 7107339 |
|  | 7107341 |
|  | 74709541 |
|  | 74723180 |
|  | 74740465 |
|  | 74746725 |
|  | 7542422 |
|  | 7542423 |
|  | 75914681 |
|  | 75914683 |
|  | 75914685 |
|  | 75914687 |
|  | 75914689 |
|  | 82395020 |
|  | 82502896 |
|  | 82502904 |
|  | 89147794 |
|  | 94442885 |
|  | 94442887 |
|  | 94442889 |
|  | 95104686 |
|  | 95104689 |
|  | 95104691 |
|  | 95104693 |
|  | 99031288 |
|  | 99031290 |
|  | NT\_086857 |
|  | 1916676 |
|  | 21930138 |
|  | AAB51214 |
|  | AAM82164 |
|  | AF525301 |
|  | U74486 |
|  | 109690031 |
|  | 109690033 |
|  | 122145633 |
|  | 54038799 |
|  | 57791992 |
|  | 602333 |
|  | 73587287 |
|  | 75047349 |
|  | 75059268 |
|  | 75061131 |
|  | 995908 |
|  | AAA96000 |
|  | AAI02441 |
|  | AAW56713 |
|  | ABG37911 |
|  | ABG37912 |
|  | AY859499 |
|  | BC102440 |
|  | CAA57348 |
|  | DQ656490 |
|  | DQ656491 |
|  | NC\_007317 |
|  | NM\_174201 |
|  | NP\_776626 |
|  | NW\_001493663 |
|  | P67939 |
|  | Q0Z9Z4 |
|  | Q29446 |
|  | Q5I2N4 |
|  | Q8MJ41 |
|  | U22145 |
|  | X81704 |
|  | 1778019 |
|  | 2829677 |
|  | AAB40617 |
|  | P79734 |
|  | U60804 |
|  | 1184935 |
|  | 29468129 |
|  | 63100839 |
|  | 82228782 |
|  | AAA97408 |
|  | AAH95597 |
|  | AAO85406 |
|  | AF365873 |
|  | AL928875 |
|  | BC095597 |
|  | NC\_007116 |
|  | NM\_131327 |
|  | NP\_571402 |
|  | NW\_001513378 |
|  | Q502Q9 |
|  | U46693 |
|  | AI437325 |
|  | AI461301 |
|  | 119610546 |
|  | 119610547 |
|  | 119610548 |
|  | 119610549 |
|  | 119610550 |
|  | 119610551 |
|  | 123983354 |
|  | 148678558 |
|  | 148678559 |
|  | 148678560 |
|  | 154369444 |
|  | 127799258 |
|  | 149053054 |
|  | 149053055 |
|  | 149053056 |
|  | 200198 |
|  | 7243329 |
|  | 4732145 |
|  | 1753088 |
|  | 13529427 |
|  | 1938364 |
|  | 871420 |
|  | 202925 |
|  | 34872015 |
|  | 37223060 |
|  | 11428461 |
|  | NW\_042919 |
|  | 30523241 |
|  | 14993573 |
|  | 11494109 |
|  | 205950 |
|  | 14993571 |
|  | NW\_044164 |
|  | 5081782 |
|  | XM\_128695 |
|  | 53575 |
|  | 53570 |
|  | 7107300 |
|  | 51828859 |
|  | NT\_039515 |
|  | 51474229 |
|  | M92424 |
|  | NT\_039520 |
|  | 2961246 |
|  | 7107338 |
|  | 7107336 |
|  | 7107334 |
|  | 7107332 |
|  | 56828 |
|  | 7107330 |
|  | 4731630 |
|  | 26348178 |
|  | 13097806 |
|  | 7107340 |
|  | NT\_039656 |
|  | 51474476 |
|  | 48094186 |
|  | 26348179 |
|  | 7107324 |
|  | 7107326 |
|  | 56157802 |
|  | 7107328 |
|  | XM\_237009 |
|  | XM\_223595 |
|  | XP\_237009 |
|  | 8400737 |
|  | 54696111 |
|  | 38194462 |
|  | 189460 |
|  | 243254 |
|  | 49979 |
|  | 11066969 |
|  | 49977 |
|  | 49976 |
|  | 49973 |
|  | NW\_042660 |
|  | 51858593 |
|  | K01700 |
|  | 8400738 |
|  | 49991 |
|  | 189478 |
|  | 28975326 |
|  | 189474 |
|  | 49981 |
|  | 49983 |
|  | 49985 |
|  | 11342598 |
|  | 49987 |
|  | 49989 |
|  | 189464 |
|  | 38049096 |
|  | 7107290 |
|  | 7107292 |
|  | 7107294 |
|  | 7107296 |
|  | 7107298 |
|  | 13591877 |
|  | 13591878 |
|  | 7107286 |
|  | 7107284 |
|  | NT\_035416.2 |
|  | 7107288 |
|  | 42821409 |
|  | XP\_223595 |
|  | 6755881 |
|  | 200202 |
|  | 200200 |
|  | 6755880 |
|  | 506432 |
|  | 506434 |
|  | 506436 |
|  | 506438 |
|  | 23491728 |
|  | 5421849 |
|  | AAA39884 |
|  | 35215 |
|  | 3041866 |
|  | 35213 |
|  | 506450 |
|  | 506452 |
|  | 35209 |
|  | 506442 |
|  | 5421827 |
|  | 506440 |
|  | 7542420 |
|  | 7542421 |
|  | 506448 |
|  | 506446 |
|  | 506444 |
|  | 5421807 |
|  | 11528485 |
|  | 191973 |
|  | 5421812 |
|  | 5524685 |
|  | 15375071 |
|  | 339815 |
|  | 339813 |
|  | XP\_128695 |
|  | MK673760 |
|  | XM\_030245924 |
|  | XM\_030245923 |
|  | XM\_030245922 |
|  | XP\_030101783 |
|  | XP\_030101784 |
|  | XP\_030101782 |
|  | QFZ95563 |

---

|  |  |
| --- | --- |
| Swiss-Prot ID | P53\_MOUSE |
|  | P53\_RAT |
|  | P53\_HUMAN |
|  | Q3UGQ1\_MOUSE |
|  | Q549C9\_MOUSE |
|  | Q53GA5\_HUMAN |
|  | Q80ZA1\_MOUSE |
|  | Q3LRW1\_HUMAN |
|  | Q3LRW2\_HUMAN |
|  | Q3LRW3\_HUMAN |
|  | Q3LRW4\_HUMAN |
|  | Q3LRW5\_HUMAN |

---

|  |  |
| --- | --- |
| Cell Localization | Cytoplasm |
|  | Nucleus |
|  | PML body |
|  | Endoplasmic reticulum |
|  | Mitochondrion matrix |
|  | Nucleus, PML body |
|  | Intracellular |
|  | Microtubule organizing center |
|  | Cytoskeleton |
|  | Centrosome |

---

|  |  |
| --- | --- |
| Ensembl ID | ENSG00000141510 |
|  | ENSP00000269305.4 |
|  | ENST00000269305.8 |
|  | ENSP00000391478.2 |
|  | ENST00000445888.6 |
|  | ENSP00000398846.2 |
|  | ENST00000455263.6 |
|  | ENSP00000482258.1 |
|  | ENST00000617185.4 |
|  | ENSP00000481179.1 |
|  | ENST00000504937.5 |
|  | ENSP00000478499.1 |
|  | ENST00000510385.5 |
|  | ENSP00000484409.1 |
|  | ENST00000504290.5 |
|  | ENSP00000478219.1 |
|  | ENST00000610292.4 |
|  | ENSP00000480868.1 |
|  | ENST00000610538.4 |
|  | ENSP00000482222.1 |
|  | ENST00000622645.4 |
|  | ENSP00000484375.1 |
|  | ENST00000619186.4 |
|  | ENSP00000481401.1 |
|  | ENST00000618944.4 |
|  | ENSP00000477531.1 |
|  | ENST00000610623.4 |
|  | ENSP00000481638.1 |
|  | ENST00000620739.4 |
|  | ENSP00000482537.1 |
|  | ENST00000619485.4 |
|  | ENSMUSG00000059552 |
|  | ENSMUSP00000127130.1 |
|  | ENSMUST00000171247.7 |
|  | ENSMUSP00000104298.3 |
|  | ENSMUST00000108658.9 |
|  | ENSRNOG00000010756 |
|  | ENSRNOP00000047840.2 |
|  | ENSRNOT00000046490.3 |
|  | ENSRNOP00000074031.1 |
|  | ENSRNOT00000085115.1 |
|  | ENSP00000391478 |
|  | ENST00000445888 |
|  | ENSP00000480868 |
|  | ENSMUSP00000104298 |
|  | ENSP00000481638 |
|  | ENSMUSP00000127130 |
|  | ENSRNOT00000046490 |
|  | ENST00000510385 |
|  | ENSP00000477531 |
|  | ENSP00000391127 |
|  | ENSP00000484375 |
|  | ENST00000610292 |
|  | ENST00000619186 |
|  | ENSP00000478499 |
|  | ENSP00000478219 |
|  | ENST00000509690 |
|  | ENSMUST00000108658 |
|  | ENST00000610538 |
|  | ENSRNOT00000085115 |
|  | ENST00000420246 |
|  | ENST00000269305 |
|  | ENSP00000484409 |
|  | ENSP00000398846 |
|  | ENSP00000481179 |
|  | ENSP00000482222 |
|  | ENST00000620739 |
|  | ENSRNOP00000074031 |
|  | ENST00000455263 |
|  | ENST00000504290 |
|  | ENSRNOP00000047840 |
|  | ENST00000610623 |
|  | ENSP00000425104 |
|  | ENST00000504937 |
|  | ENSMUST00000171247 |
|  | ENSP00000482537 |
|  | ENSP00000482258 |
|  | ENST00000618944 |
|  | ENSP00000481401 |
|  | ENST00000617185 |
|  | ENSP00000269305 |
|  | ENST00000619485 |
|  | ENST00000622645 |

---

|  |  |
| --- | --- |
| MGI ID | MGI:98834 |
|  | 98834 |

---

|  |  |
| --- | --- |
| RGD ID | 3889 |

---

|  |  |
| --- | --- |
| TCDB ID | 1.C.110.1.1 |

---

|  |  |
| --- | --- |
| Unigene ID | Hs.437460 |
|  | Rn.54443 |
|  | Hs.740601 |
|  | Mm.222 |
|  | Hs.654481 |
|  | Hs.408312 |
|  | Bt.49712 |
|  | Dr.75100 |
|  | Hs.1846 |

---

|  |  |
| --- | --- |
| Homologene ID | 460 |
|  | 7157 |
|  | 22059 |
|  | 24842 |

---

|  |  |
| --- | --- |
| Shape | O-vertex |

---

|  |  |
| --- | --- |
| IPI ID | IPI01009016 |
|  | IPI00895905 |
|  | IPI00973801 |
|  | IPI00375319 |
|  | IPI00894418 |
|  | IPI00796188 |
|  | IPI00406306 |
|  | IPI01008713 |
|  | IPI00025087 |
|  | IPI00734784 |
|  | IPI01009039 |
|  | IPI00648350 |
|  | IPI00816804 |
|  | IPI00902894 |
|  | IPI00654847 |
|  | IPI00190116 |
|  | IPI00203524 |
|  | IPI00189385 |

---

|  |  |
| --- | --- |
| Proteome Biochemical Function | Hydrolase |
|  | DNA-binding protein |
|  | Nuclease [endo, exo, ribo, deoxyribo] |
|  | Inhibitor or repressor |

---

|  |  |
| --- | --- |
| Chromosome position | 10q24 |
|  | 11 39.0 cM |
|  | 17p13.1 |

---

|  |  |
| --- | --- |
| Microarray ID | 104154\_at |
|  | 104275\_g\_at |
|  | L0249H08-3 |
|  | 1880\_AT |
|  | 1367831\_at |
|  | 201746\_at |
|  | 1426538\_a\_at |
|  | OH7728 |
|  | NM\_011640 |
|  | H3026D07-5 |
|  | 134478\_r\_at |
|  | 38 |
|  | 211300\_s\_at |
|  | 326846\_Rn |
|  | 1367830\_a\_at |
|  | A\_14\_P100302 |
|  | X01405 |
|  | A\_42\_P619917 |
|  | 1974\_s\_at |
|  | A\_23\_P26810 |
|  | 1370752\_a\_at |
|  | 1939\_at |
|  | Msa.1049.0\_at |
|  | k01700-2\_s\_at |
|  | 1438808\_at |
|  | A\_65\_P10093 |
|  | 136996\_r\_at |
|  | R553 |
|  | M22898\_at |
|  | 168990\_r\_at |
|  | A\_65\_P14116 |
|  | 31618\_AT |
|  | OR0723 |
|  | 1457623\_x\_at |
|  | K03199 |
|  | M241 |
|  | 168098\_r\_at |
|  | A\_51\_P184738 |
|  | A\_14\_P122951 |
|  | 1427739\_a\_at |
|  | x13058\_at |
|  | A\_14\_P104532 |
|  | A\_23\_P26807 |
|  | I\_958219 |

---

|  |  |
| --- | --- |
| Homo sapiens Chromosome position | 17p13.1 |

---

|  |  |
| --- | --- |
| Ensembl Transcript ID | ENST00000269305 |
|  | ENST00000359597 |

---

|  |  |
| --- | --- |
| ENSEMBL | ENSG00000141510 |

---

|  |  |
| --- | --- |
| Proteome Cellular Role | DNA repair |

---

|  |  |
| --- | --- |
| FunctionalClass | ATP binding |
|  | DNA binding |
|  | DNA strand annealing |
|  | RNA polymerase II transcription factor |
|  | copper ion binding |
|  | enzyme binding |
|  | nuclease |
|  | protein N-terminus binding |
|  | protein binding |
|  | protein heterodimerization |
|  | transcription factor |
|  | zinc ion binding |
|  | metal ion binding |
|  | transcription activator |
|  | transcription regulator |

---

|  |  |
| --- | --- |
| Molecular Function | Transcription Factor |

---

|  |  |
| --- | --- |
| Hugo Symbol | TP53 |

---

|  |  |
| --- | --- |
| Bos taurus Chromosome position | 19q15 |

---

|  |  |
| --- | --- |
| Mus musculus Chromosome position | 11 39.0 cM |

---

|  |  |
| --- | --- |
| Proteome Subcellular Localization | Nuclear |

---

|  |  |
| --- | --- |
| Compartment | void |
|  | nucleus |
|  | DNA |

---

|  |  |
| --- | --- |
| KEGG pathway | Amyotrophic lateral sclerosis (ALS) |
|  | Apoptosis |
|  | Basal cell carcinoma |
|  | Cell cycle |
|  | Chronic myeloid leukemia |
|  | Colorectal cancer |
|  | Endometrial cancer |
|  | Glioma |
|  | Huntington's disease |
|  | MAPK signaling pathway |
|  | Melanoma |
|  | Non-small cell lung cancer |
|  | Pancreatic cancer |
|  | Small cell lung cancer |
|  | Thyroid cancer |
|  | Wnt signaling pathway |

---

|  |  |
| --- | --- |
| RefSeq ACCNUM | NP\_000537 |

---

|  |  |
| --- | --- |
| State | unphosphorylated |
|  | p |
|  | u |

---

|  |  |
| --- | --- |
| Rattus norvegicus Chromosome position | 10q24 |

---

|  |  |
| --- | --- |
| Ensembl Gene ID | ENSG00000141510 |

---

|  |  |
| --- | --- |
| Keyword | telomerase |
|  | telomerase activity |
|  | cell cycle regulator |
|  | anti-oncogene |
|  | nuclei |
|  | RAS small monomeric GTPase |
|  | tumor suppressor |
|  | ubiquitin |
|  | nuclear body |
|  | antioncogene |
|  | transcription factor |
|  | RNA |
|  | nitroreductase |
|  | 14-3-3 |
|  | cytoplasm |
|  | deacetylation |
|  | DNA |
|  | transcription activating factor |
|  | exonuclease |
|  | oncogene |
|  | DNA binding activity |
|  | nucleus |

---

|  |  |
| --- | --- |
| ProteinAtlas NormalTissue | Tissue: adrenal gland; Cell type: glandular cells; Level: None; Expression type: APE; Reliability: High |
|  | Tissue: appendix; Cell type: glandular cells; Level: None; Expression type: APE; Reliability: High |
|  | Tissue: appendix; Cell type: lymphoid tissue; Level: None; Expression type: APE; Reliability: High |
|  | Tissue: bone marrow; Cell type: hematopoietic cells; Level: None; Expression type: APE; Reliability: High |
|  | Tissue: breast; Cell type: glandular cells; Level: None; Expression type: APE; Reliability: High |
|  | Tissue: bronchus; Cell type: respiratory epithelial cells; Level: None; Expression type: APE; Reliability: High |
|  | Tissue: cerebellum; Cell type: cells in granular layer; Level: None; Expression type: APE; Reliability: High |
|  | Tissue: cerebellum; Cell type: cells in molecular layer; Level: None; Expression type: APE; Reliability: High |
|  | Tissue: cerebellum; Cell type: Purkinje cells; Level: None; Expression type: APE; Reliability: High |
|  | Tissue: cerebral cortex; Cell type: glial cells; Level: None; Expression type: APE; Reliability: High |
|  | Tissue: cerebral cortex; Cell type: neuronal cells; Level: None; Expression type: APE; Reliability: High |
|  | Tissue: cervix, uterine; Cell type: glandular cells; Level: None; Expression type: APE; Reliability: High |
|  | Tissue: cervix, uterine; Cell type: squamous epithelial cells; Level: None; Expression type: APE; Reliability: High |
|  | Tissue: colon; Cell type: glandular cells; Level: None; Expression type: APE; Reliability: High |
|  | Tissue: duodenum; Cell type: glandular cells; Level: None; Expression type: APE; Reliability: High |
|  | Tissue: epididymis; Cell type: glandular cells; Level: None; Expression type: APE; Reliability: High |
|  | Tissue: esophagus; Cell type: squamous epithelial cells; Level: Medium; Expression type: APE; Reliability: High |
|  | Tissue: fallopian tube; Cell type: glandular cells; Level: None; Expression type: APE; Reliability: High |
|  | Tissue: gall bladder; Cell type: glandular cells; Level: None; Expression type: APE; Reliability: High |
|  | Tissue: heart muscle; Cell type: myocytes; Level: None; Expression type: APE; Reliability: High |
|  | Tissue: hippocampus; Cell type: glial cells; Level: None; Expression type: APE; Reliability: High |
|  | Tissue: hippocampus; Cell type: neuronal cells; Level: None; Expression type: APE; Reliability: High |
|  | Tissue: kidney; Cell type: cells in glomeruli; Level: None; Expression type: APE; Reliability: High |
|  | Tissue: kidney; Cell type: cells in tubules; Level: None; Expression type: APE; Reliability: High |
|  | Tissue: lateral ventricle; Cell type: glial cells; Level: None; Expression type: APE; Reliability: High |
|  | Tissue: lateral ventricle; Cell type: neuronal cells; Level: None; Expression type: APE; Reliability: High |
|  | Tissue: liver; Cell type: bile duct cells; Level: None; Expression type: APE; Reliability: High |
|  | Tissue: liver; Cell type: hepatocytes; Level: None; Expression type: APE; Reliability: High |
|  | Tissue: lung; Cell type: macrophages; Level: None; Expression type: APE; Reliability: High |
|  | Tissue: lung; Cell type: pneumocytes; Level: None; Expression type: APE; Reliability: High |
|  | Tissue: lymph node; Cell type: germinal center cells; Level: None; Expression type: APE; Reliability: High |
|  | Tissue: lymph node; Cell type: non-germinal center cells; Level: None; Expression type: APE; Reliability: High |
|  | Tissue: nasopharynx; Cell type: respiratory epithelial cells; Level: None; Expression type: APE; Reliability: High |
|  | Tissue: oral mucosa; Cell type: squamous epithelial cells; Level: Medium; Expression type: APE; Reliability: High |
|  | Tissue: ovary; Cell type: follicle cells; Level: None; Expression type: APE; Reliability: High |
|  | Tissue: ovary; Cell type: ovarian stroma cells; Level: None; Expression type: APE; Reliability: High |
|  | Tissue: pancreas; Cell type: exocrine glandular cells; Level: None; Expression type: APE; Reliability: High |
|  | Tissue: pancreas; Cell type: islets of Langerhans; Level: None; Expression type: APE; Reliability: High |
|  | Tissue: parathyroid gland; Cell type: glandular cells; Level: None; Expression type: APE; Reliability: High |
|  | Tissue: placenta; Cell type: decidual cells; Level: None; Expression type: APE; Reliability: High |
|  | Tissue: placenta; Cell type: trophoblastic cells; Level: None; Expression type: APE; Reliability: High |
|  | Tissue: prostate; Cell type: glandular cells; Level: None; Expression type: APE; Reliability: High |
|  | Tissue: rectum; Cell type: glandular cells; Level: None; Expression type: APE; Reliability: High |
|  | Tissue: salivary gland; Cell type: glandular cells; Level: None; Expression type: APE; Reliability: High |
|  | Tissue: seminal vesicle; Cell type: glandular cells; Level: None; Expression type: APE; Reliability: High |
|  | Tissue: skeletal muscle; Cell type: myocytes; Level: None; Expression type: APE; Reliability: High |
|  | Tissue: skin; Cell type: epidermal cells; Level: Low; Expression type: APE; Reliability: High |
|  | Tissue: small intestine; Cell type: glandular cells; Level: None; Expression type: APE; Reliability: High |
|  | Tissue: smooth muscle; Cell type: smooth muscle cells; Level: None; Expression type: APE; Reliability: High |
|  | Tissue: spleen; Cell type: cells in red pulp; Level: None; Expression type: APE; Reliability: High |
|  | Tissue: spleen; Cell type: cells in white pulp; Level: None; Expression type: APE; Reliability: High |
|  | Tissue: stomach, lower; Cell type: glandular cells; Level: None; Expression type: APE; Reliability: High |
|  | Tissue: stomach, upper; Cell type: glandular cells; Level: None; Expression type: APE; Reliability: High |
|  | Tissue: testis; Cell type: cells in seminiferus ducts; Level: None; Expression type: APE; Reliability: High |
|  | Tissue: testis; Cell type: Leydig cells; Level: None; Expression type: APE; Reliability: High |
|  | Tissue: thyroid gland; Cell type: glandular cells; Level: None; Expression type: APE; Reliability: High |
|  | Tissue: tonsil; Cell type: germinal center cells; Level: None; Expression type: APE; Reliability: High |
|  | Tissue: tonsil; Cell type: non-germinal center cells; Level: None; Expression type: APE; Reliability: High |
|  | Tissue: tonsil; Cell type: squamous epithelial cells; Level: Low; Expression type: APE; Reliability: High |
|  | Tissue: urinary bladder; Cell type: urothelial cells; Level: Low; Expression type: APE; Reliability: High |
|  | Tissue: uterus, post-menopause; Cell type: cells in endometrial stroma; Level: None; Expression type: APE; Reliability: High |
|  | Tissue: uterus, post-menopause; Cell type: glandular cells; Level: None; Expression type: APE; Reliability: High |
|  | Tissue: uterus, pre-menopause; Cell type: cells in endometrial stroma; Level: None; Expression type: APE; Reliability: High |
|  | Tissue: uterus, pre-menopause; Cell type: glandular cells; Level: None; Expression type: APE; Reliability: High |
|  | Tissue: vagina; Cell type: squamous epithelial cells; Level: None; Expression type: APE; Reliability: High |
|  | Tissue: vulva/anal skin; Cell type: epidermal cells; Level: Low; Expression type: APE; Reliability: High |

---

|  |  |
| --- | --- |
| LocatorString | gscquery:curcumin+prostate+cancer |
|  | pmquery:curcumin+prostate+cancer |
|  | pmquery:curcumin prostate cancer |
|  | pmquery:invasive+breast+cancer |

---

|  |  |
| --- | --- |
| Proteome Organismal Role | Cell death/Apoptosis |

---
